# Smoke-charged vortex doubles hemispheric aerosol in the middle stratosphere and buffers ozone depletion

**DOI:** 10.1126/sciadv.adn3657

**Published:** 2024-07-12

**Authors:** Chaoqun Ma, Hang Su, Jos Lelieveld, William Randel, Pengfei Yu, Meinrat O. Andreae, Yafang Cheng

**Affiliations:** ^1^ Minerva Research Group, Max Planck Institute for Chemistry, 55128 Mainz, Germany.; ^2^ Key Laboratory of Atmospheric Environment and Extreme Meteorology, Institute for Atmospheric Physics, Chinese Academy of Science, Beijing 100029, China.; ^3^ Atmospheric Chemistry Department, Max Planck Institute for Chemistry, 55128 Mainz, Germany.; ^4^ Atmospheric Chemistry Observations and Modeling, National Center for Atmospheric Research, Boulder, CO 80307, USA.; ^5^ Institute for Environmental and Climate Research, Jinan University, Guangzhou 510630, China.; ^6^ Biogeochemistry Department, Max Planck Institute for Chemistry, 55128 Mainz, Germany.; ^7^ Scripps Institution of Oceanography, University of California San Diego, La Jolla, CA 92037, USA.

## Abstract

Australian mega-wildfires in the summer of 2019-2020 injected smoke into the stratosphere, causing strong ozone depletion in the lower stratosphere. Here, we model the smoke plume and reproduce its unexpected trajectory toward the middle stratosphere at ~35-kilometer altitude. We show that a smoke-charged vortex (SCV) induced and maintained by absorbing aerosols played a key role in lofting pollutants from the lower stratosphere and nearly doubled the southern hemispheric aerosol burden in the middle stratosphere. The SCV caused a redistribution of stratospheric aerosols, which boosted heterogeneous chemistry in the middle stratosphere and enhanced ozone production, compensating for up to 70% of the ozone depletion in the lower stratosphere. As global warming continues, we expect a growing frequency and importance of SCVs in promoting the impacts of wildfires on stratospheric aerosols and chemistry.

## Teaser

Wildfires can induce vast vortices that carry large amounts of aerosol to the middle stratosphere and affect the ozone layer.

## INTRODUCTION

Aerosols in the middle stratosphere (above 20 to 25 km and below 35 to 40 km) have important impacts on the ozone layer, Earth’s energy balance, and climate (*1*–*5*). Intense volcanic eruptions and transport by the Brewer-Dobson circulation have been considered to be the main drivers of aerosol abundance in the middle stratosphere (*6*, *7*), adding to the sulfate formed from carbonyl sulfide transported from the troposphere (*8*), interplanetary particle debris, and meteoric dust (*7*, *9*, *10*). Recently, the 2019-2020 mega-bushfires in Australia have resulted in an enormous, long-lived (~13 weeks) “aerosol bubble” with a dimension of thousands of kilometers, rising deeply into the stratosphere (~35 km) (*11*, *12*). It rivaled major volcanic perturbations and is suggestive of an unexpected but important contribution of wildfires to aerosols in the middle stratosphere.

Pyro-convection and self-lofting of smoke plumes have been recognized as key processes in the transport of wildfire emissions to high altitudes (*13*–*16*). However, they generally do not provide sufficient buoyancy to loft plumes into the middle stratosphere (*17*, *18*). It has been suggested that the observed long-lived “smoke-charged vortex (SCV),” an anticyclonic vortex that confined the aerosol bubble, has played a critical role in transporting the 2019-2020 Australian bushfire smoke plumes to the middle stratosphere (*11*, *12*). However, reproducing the observed SCV from genesis to dissipation has been a major challenge for model simulations, which hinders our understanding of their impacts on smoke transport and stratospheric chemistry (section S2.1 and fig. S1) (*14*, *18*–*21*).

By combining satellite observations with numerical model calculations, we investigated the transport process of the 2019-2020 Australian mega-bushfire to the stratosphere and its impact on aerosol burden and chemistry in the middle stratosphere, focusing on the role of SCVs that have been largely overlooked in the past. To model the major SCV event during the 2019-2020 Australian mega-bushfire (see Materials and Methods), we assimilated the satellite observed aerosol index (AI) and aerosol optical depth (AOD) during the period with intensive pyro-convection in a regional data assimilation system with high spatial and time resolution (see Materials and Methods). This enabled us to assign accurate initial conditions to a global chemistry-climate model about the position and amount of smoke aerosols released by the pyro-convection to simulate the transport of pollutants and impacts on stratospheric aerosols and ozone. In the global simulations, we did not apply nudging (data assimilation) to the reanalysis of meteorological fields of the investigated SCV, allowing free interactions between the model dynamics with smoke aerosols and the heating effect of the smoke, e.g., from black carbon (BC) particles (see Materials and Methods and fig. S2).

## RESULTS

### Model versus observation

**Fig. 1.**
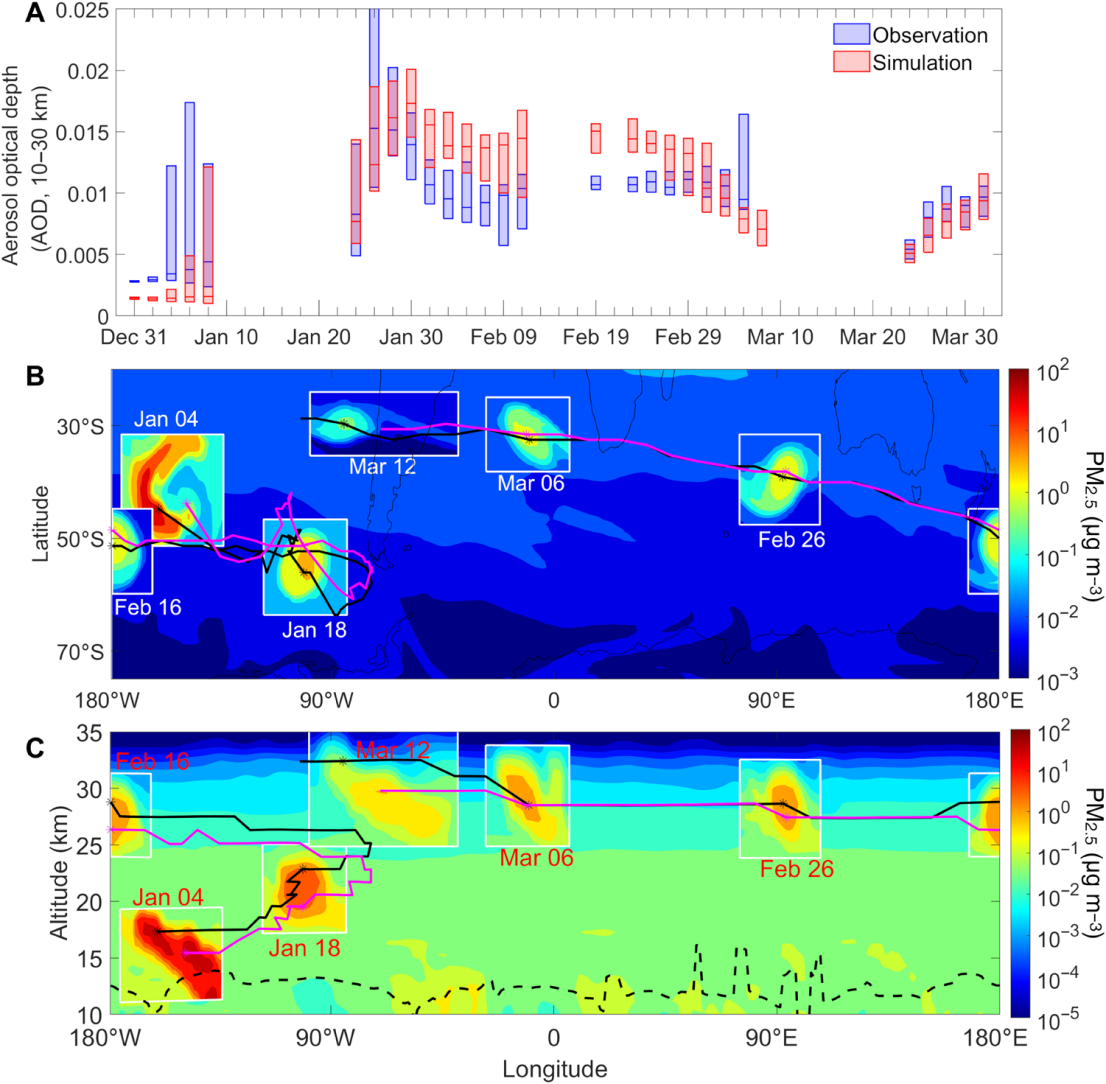
Evolution of biomass burning (BB) smoke and SCV. (**A**) Comparison of AOD at 1020-nm wavelength between SAGE III-ISS satellite observations and model simulations (scenario Base) in the extratropical Southern Hemisphere (ESH; between 25°S and 90°S). “SAGE observations” AODs were calculated by integrating available measurement data of aerosol extinction from 10- to 30-km altitude during a 48-hour period, and “CAM-Chem simulation” AODs were modeled by applying the same sampling criteria (time and location) to the model output. The upper, middle, and lower lines in the whisker plots represent the 25th, 50th, and 75th percentiles of the sampled 48-hour AOD data. (**B**) Horizontal view of PM_2.5_. The boxes show the evolution of PM_2.5_ concentrations across the SCV center (grid with maximum Lait potential vorticity, LPV); the magenta (or black) thick lines and asterisks mark the trajectory and positions of the SCV center from Modern-Era Retrospective analysis for Research and Applications Version 2 (MERRA2) reanalysis (or model simulations). The background (areas out of boxes) represents PM_2.5_ concentrations at 00 UTC on 15 February 2020 at 15 hPa (~29 km) from the simulation without wildfire events. (**C**) Vertical section of PM_2.5_. The boxes show the vertical sections across the aerosol bubble center; black (or magenta) solid lines, and asterisks coincide with (B). The background slice is taken at the same time as (B) but at latitude 45°S. The dashed line marks the tropopause.

Overall, our model simulations reproduce the total aerosol loading and plume rise well, based on comparison with the observed AOD from Stratospheric Aerosol and Gas Experiment III (SAGE III)–International Space Station (ISS) satellite observation and the vertical profile of aerosol extinction coefficients from ground-based Lidar and the Cloud-Aerosol Lidar with Orthogonal Polarization (CALIOP) on the CALIPSO satellite (Fig. 1A, sections S2.2 and S2.3, and figs. S3 to S6). We retrieved an increase of 0.8 Tg in stratospheric aerosol mass in the extratropical Southern Hemisphere (ESH; 25°S to 90°S) from the Australian New Year Super Outbreak [ANYSO; (*5*)] (29 to 31 December 2019 and 04 January 2020) pyro-convections, which is consistent with the previous estimate of 0.3 to 1.1 Tg based on satellite observations (*5*).

In this outbreak, three SCVs (*11*, *12*, *22*) were identified from the ANYSO event. Here, we only focused on the strongest one that rose to 35 km with a substantial impact on the chemistry of the middle stratosphere (see Materials and Methods). The observed evolution of the investigated SCV is also well reproduced by the model. In Fig. 1 (B and C), we calculated the potential vorticity (PV) as Lait PV (LPV) (section S2.4) based on Modern-Era Retrospective analysis for Research and Applications Version 2 (MERRA2) reanalysis data (*23*) and track positions of the vortex center from the maximum LPV among a compact LPV anomaly. The trajectory of the modeled SCV (black lines) agrees well with that of the maximum LPV from MERRA2 reanalysis (magenta lines). The vertical evolution of temperature perturbations within the SCV simulated by our model is also in agreement with observations (fig. S7). After genesis in early January, the vortex traveled along the westerlies from the South Pacific to the Drake Passage with an ascent velocity of ~0.3 km day^−1^. Subsequently, the SCV entered the easterlies and traveled westward (fig. S8B). After circumnavigating Earth for 40 days, the SCV gradually dissipated over western Chile. During this period, the ascent of the SCV dropped to ~0.15 km day^−1^ as a result of the gradually decreasing BC concentration in the plume. The ascent velocities simulated are very close to the observed 0.28 and 0.16 km day^−1^ (*24*).

### Evolution of the SCV

**Fig. 2.**
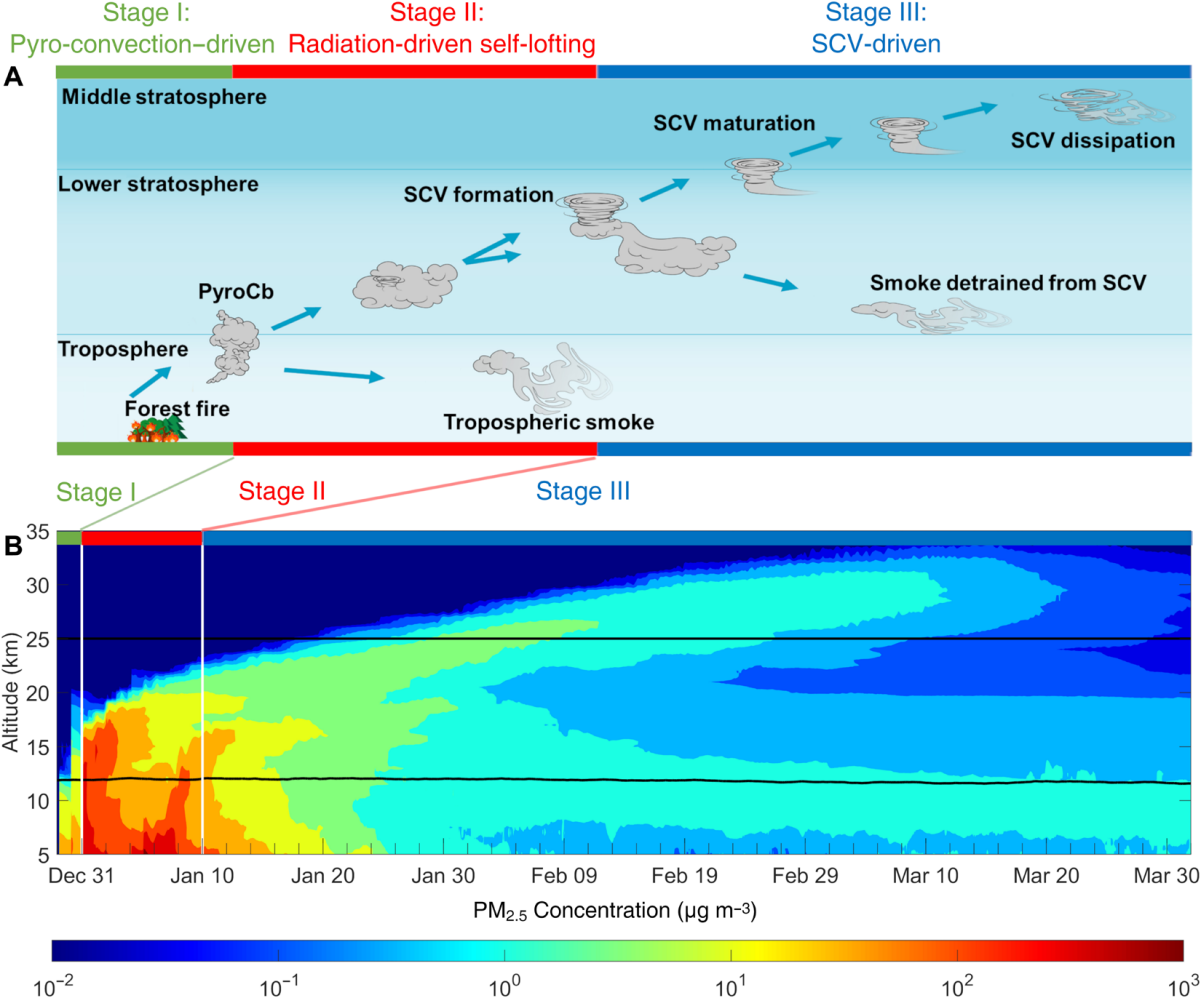
Schematic illustration of wildfire plume rising from the ground to the middle stratosphere. (**A**) Schematic plume rise in three stages. In stage I, BB aerosol was injected into the upper troposphere and lowermost stratosphere by pyro-convection. In stage II, the stratospheric part continued rising by black carbon self-lofting, and a vortex was forming inside the plume. The tropospheric part decayed. Stage III began when the SCV matured and departed from the main plume. Aerosol inside the SCV rose into the middle stratosphere until the dissipation of the SCV, while aerosol outside the vortex lost updraft velocity while diluting. (**B**) Maximum PM_2.5_ concentrations in ESH from the Base simulation. Black lines mark the averaged tropopause and the bottom of the middle stratosphere. White lines mark the border between different stages corresponding to those shown in (B) (see fig. S9 for details).

As shown in Fig. 2, the transport of the 2019-2020 Australian bushfire smoke to the middle stratosphere underwent three stages. Stage I was driven by pyro-convection (29 to 31 December 2019), where strong wildfires triggered deep convective transport and transferred the smoke aerosol up to ~16 km, reaching the upper troposphere and lowermost stratosphere, as also observed from satellite (*5*). Stage II was a radiation-driven lofting process (31 December 2019 to 10 January 2020), where heating by light-absorbing aerosols such as BC became the main driver [so-called “self-lofting” (*14*)] and carrying the plume top from ~16 to ~21 km. Stage III was SCV-driven (from 10 January onward), where the vortex was formed and together with the smoke within rose to ~35 km. The SCV formation represents a key mechanism for transporting the smoke aerosols from the lower to the middle stratosphere. Without the help of SCV in stage III, the smoke plume would have gradually lost its buoyancy due to dilution and reached only ~25 km (fig. S9D). In the following, we will elucidate the full life cycle of the SCV from genesis through maturation to dissipation.

Aerosol heating effects have been suggested to play an important role in the evolution of SCVs (*11*, *12*, *20*, *22*, *24*). Their impact on the vortex development can be described by the quasi-geostrophic PV equation (*25*) with an additional aerosol heating term

$$\frac{D_{g}}{\mathrm{Dt}}q=-f_{0}\frac{\partial }{\partial p}\left[\frac{\kappa \left(J+J_{\mathrm{BC}}\right)}{\sigma p}\right]$$ (1)

Here, *q* represents the quasi-geostrophic PV, *p* is the air pressure, κ is the ratio of the gas constant to the specific heat at constant pressure, σ is the standard atmosphere static stability parameter; and *f*_0_ is a linearized approximation of the Coriolis parameter or planetary vorticity. *J* is the original diabatic heating term due to the heating effect, e.g., of water vapor in the quasi-geostrophic PV equation; and *J*_*BC*_ represents the additional heating from absorbing aerosols such as BC. According to Eq. 1, $-f_{0}\frac{\partial }{\partial p}\left[\frac{\kappa \left(J+J_{\mathrm{BC}}\right)}{\sigma p}\right]$ will change the PV and thus affect the vortex development through the diabatic heating effect (DHE; dominated by *J_BC_* here; section S2.4).

**Fig. 3.**
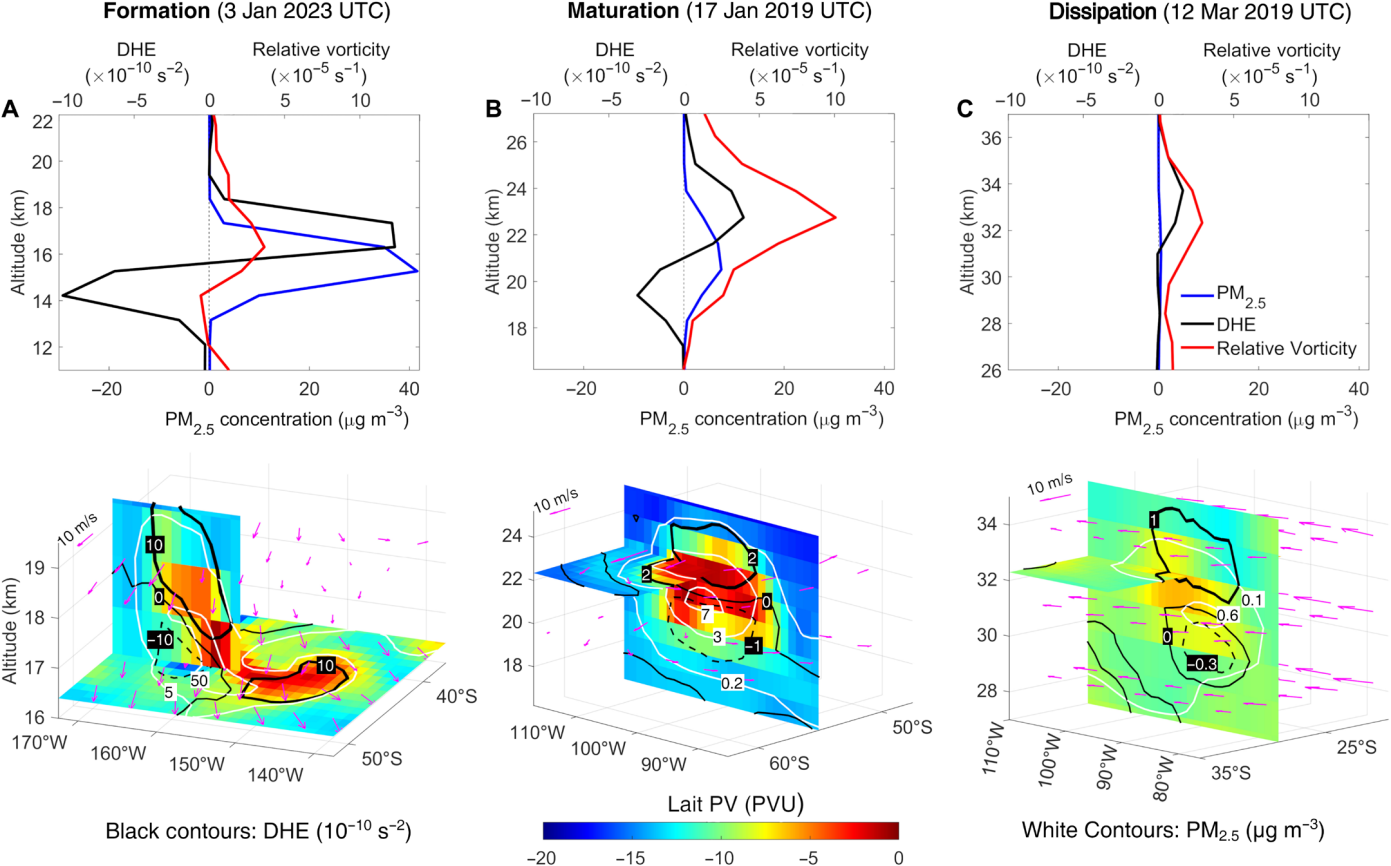
Evolution of key parameters of the SCV. Profiles of vorticity, diabatic heating effect (DHE) and PM2.5 concentration across the center of the SCV (top panels) with its corresponding 3D structure (lower panels) at different periods. Results are shown for the formation (**A**), maturation (**B**) and dissipation (**C**) period of the SCV. For the 3D plot, the colors represent the LPV, and the vertical and horizontal cross sections were taken at the maximum LPV point; the PM_2.5_ concentration (in micrograms per cubic meter) is shown by the white contour lines and the DHE (10^−10^ s^−2^) by black contour lines (solid lines: positive; dashed lines: negative). Wind vectors are also shown. See figs. S18 and S19 for more details.

Figure 3 shows the evolution of the SCV, from formation to maturation and dissipation. In the formation period, a positive PV anomaly overlapped with the smoke plume, the center of which was slightly lower than that of the PV anomaly (Fig. 3A). This positive PV anomaly could be traced back to a PV anomaly that already existed at 00:00 UTC on 30 December, several hours before the smoke was injected to that height (fig. S10). According to Eq. 1, the reduced BC concentration and heating *J*_*BC*_ from the center toward the top of the plume resulted in a positive DHE and an increase of *q* at the upper part of the plume. Because the anomaly was located at the upper part of the plume, the increased *q* will enhance the positive vorticity there (Fig. 3A). The enhanced vorticity concentrates the ambient smoke toward the center (fig. S11), which thus yields a more localized BC distribution. In other words, the enhanced vorticity confines the BC heating, preventing dissipation and dilution, which maintains the positive DHE and vorticity, forming a positive feedback mechanism. Under the current conditions, a single-scattering albedo (SSA) of 0.85 to 0.90 generated sufficient heating of the smoke plume to trigger the SCV formation (section S2.5).

After the plume developed into an ellipsoidal aerosol bubble on 10 January (with a maximum diameter of ~2000 km; fig. S11), it entered the maturation period. The ellipsoidal aerosol bubble continued to rise, driven by the positive feedback discussed above. Smoke aerosols that detrained from the vortex gradually diluted and stopped rising, resulting in another distinct smoke plume as detected by satellite (lower branch in Fig. 2B and fig. S12) and ground-based Lidar observations (*26*). As shown in Fig. 3B, the DHE was positive above the center of the aerosol bubble and negative in the lower part. The negative DHE dampened the lower part of the vortex allowing mixing and dilution with surrounding air (like a plume leakage, in the shape of an aerosol tail in Fig. 3B and section S2.6). The positive DHE enhanced the vortex which confined and carried part of the aerosols, maintaining the plume lofting. Without the heating effect of smoke aerosols and the positive feedback, even a preexisting SCV such as that on 10 January would not last long (only ~10 days; section S2.4).

The dissipation period started when the aerosol bubble entered the middle stratosphere in mid-February, which agrees with the observed time series of SCV properties (*24*). There, it moved from a region of calm zonal wind to that with strong easterlies (fig. S8B) associated with strong latitudinal wind shear (fig. S13B). In the region of easterlies, the wind shear stretched the SCV into a slant strip (Fig. 3C and fig. S14) and separated the center of aerosol DHE and the vortex (fig. S13B). This interrupted the positive feedback and led to dissipation of the vortex (fig. S13A) and diluted the plume across the ESH. At an altitude above 25 km, the gradient of ozone heating may also reduce the positive DHE (increase negative DHE) and suppress the vortex (fig. S8A).

### Impact on aerosol loading

**Fig. 4.**
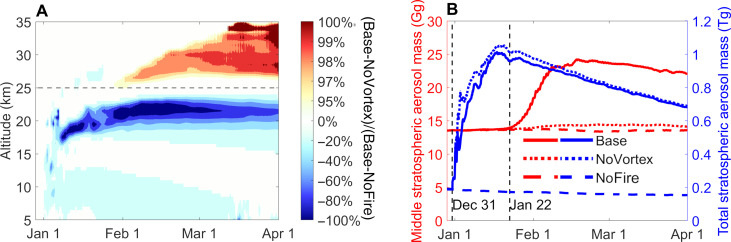
Impact of SCV and BB on stratospheric aerosol concentrations. (**A**) The colors mark the contribution of SCV formation to PM_2.5_ in the ESH during the 2019-2020 Australian wildfire events. The contribution of the SCV is calculated by the fraction of changes of PM_2.5_ caused by SCV (Base-NoVortex) in the total changes caused by the wildfires (Base-NoFire). See Materials and Methods and section S1.5 for details about the simulation scenarios. (**B**) Temporal evolution of total PM_2.5_ mass in the ESH middle stratosphere and the entire stratosphere. The two vertical dashed lines mark the onsets of increasing total stratospheric and middle stratospheric aerosol masses.

To quantify the impact of the SCV, we performed additional model simulations without SCV and without wildfire emissions for comparison (see Materials and Methods). As shown in Fig. 4A, the SCV dominated the transport of smoke to the middle stratosphere (above 25 km) with a contribution of >90%, while the self-lofting can only transport the smoke to 15- to 25-km altitude (figs. S9D and S15B). Compared to the case with only self-lofting, the SCV, in principle, led to a redistribution of aerosols by carrying them from the lower to the middle stratosphere (Fig. 4A). In total, around 11-Gg smoke aerosols were transported to the middle stratosphere, which almost doubled the overall aerosol mass of the middle stratosphere in the ESH during late February (Fig. 4B). Note that compared to CALIPSO observations, on average, the simulated light extinction of the “aerosol bubble” may have been underestimated by up to ~50% (section S2.3), and thus, the enhancement of aerosol loading in the middle stratosphere due to SCVs may even be conservatively estimated.

### Impact on stratospheric ozone chemistry

**Fig. 5.**
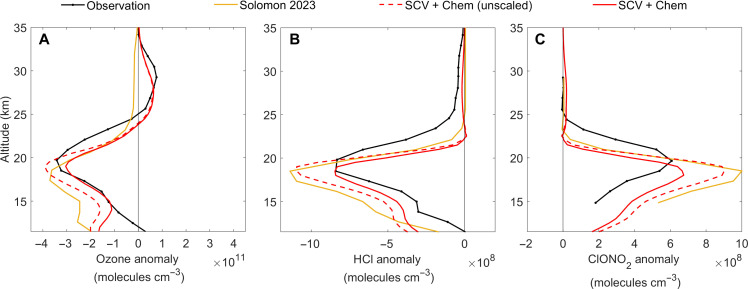
Impact of SCV and heterogeneous reactions on stratospheric ozone, HCl and ClONO_2_ from 30°S to 50°S in June–July 2020. Results are shown for ozone (**A**), HCl (**B**) and ClONO_2_ (**C**).Simulated anomalies are calculated as the difference between a specific case and the no wildfire control case (NoFire). The simulation “SCV + Chem (unscaled)” is the same as “SCV + Chem” except that the solubility of HCl was not scaled and adopted the same value as in (*27*) (see Materials and Methods). “Solomon 2023” represents the anomaly profile taken directly from Solomon *et al*. (*27*). Observed O_3_ anomalies come from Microwave Limb Sounder (MLS), while HCl and ClONO_2_ anomalies are from Atmospheric Chemistry Experiment Fourier transform spectrometer (ACE-FTS), which represent the average of June and July 2020 minus that of 2005–2019.

By changing the abundance and distribution of stratospheric aerosols, SCVs can strongly influence stratospheric chemistry and the ozone layer. To investigate this aspect, we included the heterogeneous reactions involving wildfire aerosols in our model simulations in analogy to Solomon *et al*. (*27*), where wildfire aerosols were treated as a mixture of oxidized organics and sulfate with increased hydrochloric acid (HCl) solubility and associated heterogeneous reaction rates (see Materials and Methods). Besides, to further improve the representation of the vertical distribution of aerosol loading in the middle and lower stratosphere, we constrain the aerosol concentration by SAGE satellite observations (see Materials and Methods). As shown in Fig. 5A, after considering reactions on aerosols transported by SCV, our model simulations can well reproduce the observed change of stratospheric ozone. Besides ozone, the vertical profile anomaly profiles of chlorine species such as HCl and ClONO_2_ were also well captured for June and July 2020 (Fig. 5, B and C).

**Fig. 6.**
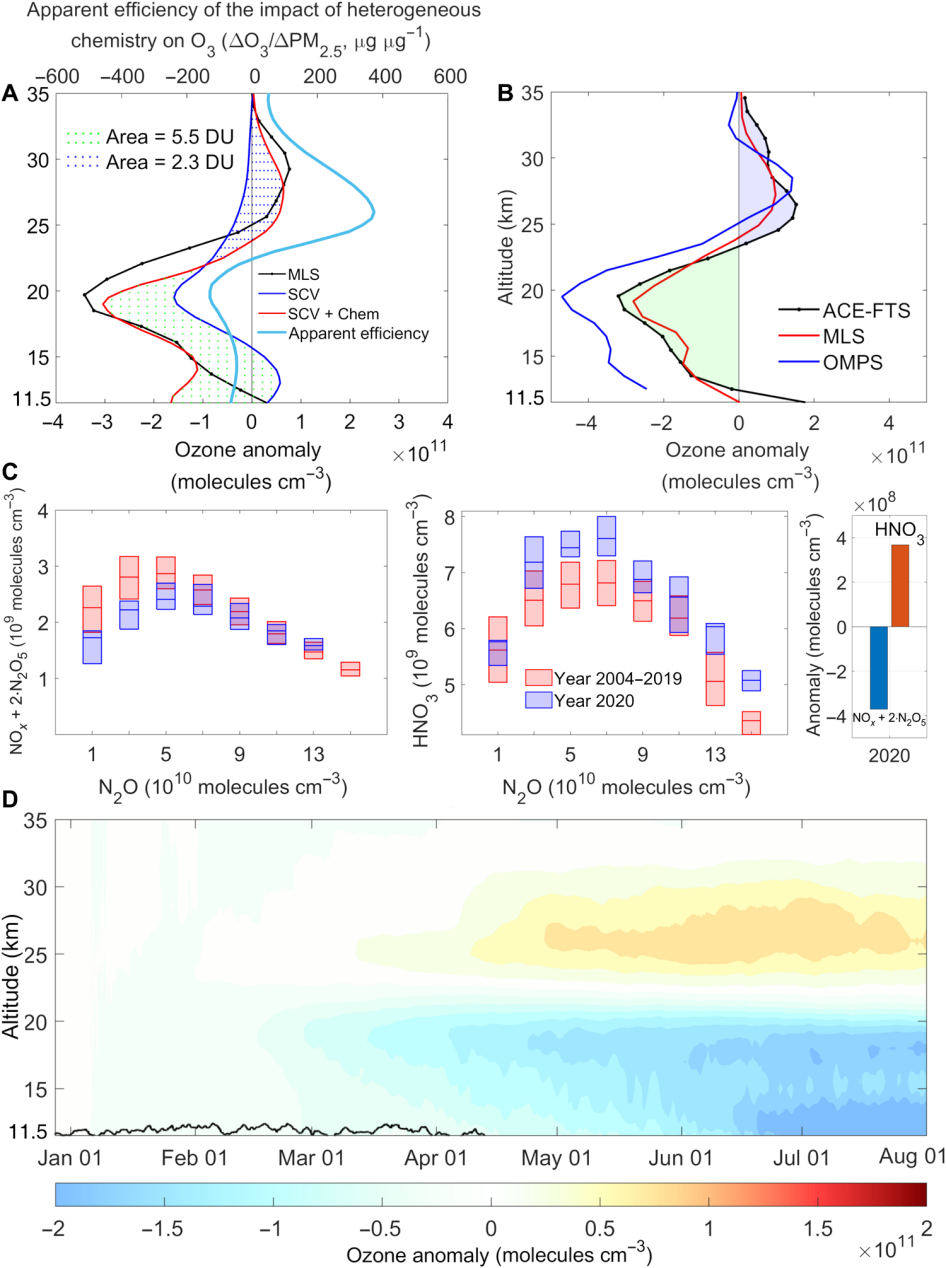
Impact of SCV and BB on stratospheric ozone. (**A**) The impact of aerosols on the O_3_ profile at latitudes from 30°S to 50°S in June–July 2020. Simulated anomalies are calculated as the difference between a specific case and the no wildfire control case (NoFire). Two specific cases are here: “SCV” represents simulations with SCV, satellite-constrained BB aerosol, but without associated heterogeneous reactions; “SCV + Chem” represents simulations with SCV, BB aerosol, and associated heterogeneous reactions. The apparent efficiency of heterogeneous reactions on ozone depletion/production is also shown in (A) (cyan line). The efficiency is calculated as the change of O_3_ due to heterogeneous reactions normalized by the change in PM_2.5_. Positive efficiencies represent O_3_ production, while negative values represent O_3_ depletion. (**B**) O_3_ anomalies from ACE-FTS, MLS, and OMPS observations averaged over 45°S to 60°S. The anomalies represent the monthly average of July 2020 minus the July of 2005–2019 for ACE-FTS and MLS while 2020 minus 2012–2019 for OMPS [ACE data source: figure 2B in (*2*)]. (**C**) ACE observed a change of NO*_x_* plus its temporary reservoir N_2_O_5_ and HNO_3_ concentrations at 26.5-km altitude over 45°S to 60°S during June and July. The left and middle panels are the concentrations of NO*_x_* + 2∙N_2_O_5_ and HNO_3_ plotted against their corresponding N_2_O concentrations, respectively (whisker boxes from 2 × 10^10^ molecules cm^−3^ bins of N_2_O). The right panel shows the ACE observed NO*_x_* + 2∙N_2_O_5_ and HNO_3_ anomaly in the year 2020 compared with their climatology average from 2004 to 2019; both anomalies are significant at a 99% significance level. (**D**) Modeled O_3_ anomalies exclusively caused by heterogeneous reactions of wildfire aerosols (the nonchemical effects have been excluded as detailed in section S2.7). The black line indicates the average tropopause position.

The important role of the SCV in regulating the wildfire effects on stratospheric chemistry is better illustrated in fig. S16A. Without the SCV, the model shows a monotonic ozone loss both in the lower and middle stratosphere (blue dashed curve in figs. S16A and S17D), which differs from observations showing ozone increase in the middle stratosphere. After accounting for the SCV, the model shows a different picture: the rising smoke and its associated heterogeneous reactions result in a net loss of ozone below ~23 km and a net increase at higher altitudes. The distinct ozone anomalies between the middle and lower stratosphere are consistent with observations from three independent satellites [the Atmospheric Chemistry Experiment (ACE), Ozone Mapping and Profiler Suite (OMPS), and Microwave Limb Sounder (MLS)]. As shown in Fig. 6 (A and B), the satellite-observed ozone anomalies in July 2020 show an ozone depletion (negative values) in the 13- to 23-km altitude range and an ozone increase (positive values) above 23-km altitude, thus changing sign at this altitude over the mid-latitude Southern Hemisphere (*2*). This pattern of ozone anomalies can only be reproduced by model simulations with heterogeneous reactions that account for SCV effects (red line in Fig. 6A), supporting the importance of such vortices in reproducing wildfire impacts on stratospheric aerosols, dynamics, and ozone chemistry.

The distinct impact of wildfire aerosols on ozone between the lower and the middle stratosphere can be explained by the unique chemical regimes in each layer. In the stratosphere, the nighttime heterogeneous uptake of N_2_O_5_ on sulfate (or sulfate-coated) particles produces HNO_3_ and reduces the gas phase nitrogen oxide (NO*_x_*) concentrations. This, in turn, inhibits the reaction of NO_2_ with ClO and HO*_x_* radicals, leading to an increasing availability of ozone-destroying ClO and HO*_x_* (*2*, *28*–*30*). In the lower stratosphere, ozone destruction is dominated by halogen chemistry, while in the middle stratosphere, NO*_x_*-catalyzed destruction plays a major role (*31*). Therefore, the NO*_x_* loss and reactive halogen increase due to the smoke-induced heterogeneous reactions leads to ozone loss in the lower stratosphere but increases ozone in the middle stratosphere.

Although the amount of wildfire aerosols transported to the mid-stratosphere was only ~3% of that reaching the lower stratosphere, the particles are much more efficient in changing ozone in the middle stratosphere than in the lower stratosphere (cyan curve in Fig. 6A). Note that besides chemical effects of the wildfire smoke plume, ozone depletion was also caused by nonchemical effects, i.e., injection ozone-poor air and changes in heating and dynamics (*18*). For example, the injection of ozone-poor air will lead to ozone decrease both in the lower and middle stratosphere. Without accounting for nonchemical effects, one will underestimate the chemical enhancement of ozone in the middle stratosphere and overestimate the chemical depletion of ozone in the lower stratosphere. To disentangle the chemical effects from nonchemical effects, we performed additional model simulations and determined the chemical effect as the difference between cases with and without heterogeneous chemistry (difference between the red and blue lines in Fig. 6A and section S2.7). Note that heterogeneous chemistry here includes both N_2_O_5_ hydrolysis and Cl-based het chemistry.

As shown in Fig. 6A, the chemical effect increased the ozone depletion by 5.5 DU in the lower stratosphere but decreased it by 2.3 DU in the middle stratosphere. This means that by lifting wildfire smoke into the middle stratosphere, SCV has largely compensated for the chemical ozone depletion in the lower stratosphere, i.e., by 70% at 30°S and by 44% at 30°S to 50°S in June 2020. Note that the amount of wildfire aerosols transported to the mid-stratosphere was only a trivial fraction of that reaching the lower stratosphere, which already largely compensated the ozone depletion in the lower stratosphere for several months (Fig. 6D). Given a stronger SCV with more smoke transported, it could potentially counteract ozone depletion, or even result in an enhancement of the ozone levels. Besides wildfire-induced chemical effects, natural climate variability like quasi-biennial oscillation (QBO) may also contribute to the observed O_3_ anomalies in the middle stratosphere, but its influence is estimated to be much less than the change due to the chemical effect (section S2.8). Moreover, the minor impact of QBO anomalies compared to that of heterogeneous chemistry is also evident from the observed change of reactive nitrogen species. In a QBO-dominated scenario, consistent changes in NO*_x_*, N_2_O_5_, and HNO_3_ were expected on the basis of their climatological correlations (section S2.8) (*32*, *33*), which is in contrast to observations showing an anticorrelation between HNO_3_ and NO*_x_* (or N_2_O_5_) in Fig. 6C. This anticorrelation aligns well with the influence of the SCV-affected heterogeneous chemistry, i.e., reaction $2{\text{NO}}_{2}⇌N_{2}O_{5}\overset{\text{Aerosol}}{\to }2{\text{HNO}}_{3}$.

## DISCUSSION

In this study, we explored the mechanisms responsible for the unexpected ascent of the Australian wildfire plume to the middle stratosphere. Our findings indicate that pyro-convection and self-lofting mechanisms are insufficient for raising the plume beyond the lower stratosphere. Instead, an SCV, driven and sustained by the absorption of aerosols, was crucial for elevating pollutants from the lower to the middle stratosphere (approximately 35 km). We demonstrate that the SCV almost doubled the aerosol load in the middle stratosphere of the Southern Hemisphere and caused a redistribution of stratospheric aerosols. This redistribution boosted heterogeneous chemical reactions in the middle stratosphere, leading to increased ozone production which offset up to 70% of the ozone depletion in the lower stratosphere.

The frequency of SCVs can be expected to increase due to global warming, as the occurrence of two essential requirements for the formation of SCVs, namely, large biomass burning (BB) events and convection, has been estimated to increase by 5% (*34*) and 3% per decade (*35*), respectively, given the current rate of climate change; in addition, the extratropical PV anomalies, which may facilitate SCV formation, will also increase by 3% per decade (sections S2.9 and S2.10) (*36*). Thus, SCVs could become an important factor for future stratospheric chemistry and climate, which will need to be accounted for in Earth system models. Moreover, SCVs could markedly increase the impacts of major natural disasters that have happened on geological timescales, e.g., leading to mass extinctions, as well as human-induced catastrophes such as firestorms ignited by nuclear conflicts (*14*, *37*–*39*).

## MATERIALS AND METHODS

### Identification of smoke-charged vortices from MERRA2

The MERRA2 (*23*) is used to provide data for nudging the Community Atmosphere Model with Chemistry (CAM-chem) model toward realistic meteorological conditions and for locating the SCV. MERRA2 assimilates a variety of modern satellite observations like atmospheric motion vectors and hyperspectral infrared radiances which enables accurate replication of the real atmospheric thermal and dynamic structure up to the mesosphere with high vertical resolution. These characteristics make MERRA2 particularly suitable for tracking the PV anomalies induced by SCVs.

From the MERRA2 reanalysis data, we identified three SCVs from the ANYSO (*5*), including two produced by 30 December and one produced by 4 January pyrocumulonimbus outbreaks (details in section S1.1). Here, we only focused on the strongest one that rose to 35 km and had a substantial impact on the chemistry of the middle stratosphere. Throughout the main text and the Supplementary Material, unless specified otherwise, the discussion on the impact of “SCV” refers to this strongest one.

### WRF-Chem/DART

WRF-Chem/DART is an atmospheric chemistry simulation and data assimilation system developed by coupling the Weather Research and Forecasting Model with chemistry (WRF-Chem) with the Data Assimilation Research Testbed (DART) (*40*, *41*). WRF-Chem is an online three-dimensional, Eulerian chemical transport model that simulates atmospheric chemistry and meteorology (*42*). DART is an open-source community facility developed by the National Center for Atmospheric Research (*43*) for ensemble-based data assimilation research. DART has a well-documented and flexible coding structure that makes it straightforward to couple with different models and apply them in various research areas (*44*, *45*). WRF-Chem/DART can assimilate atmospheric meteorological and chemical observations from a variety of in situ and remote observation platforms and has proven to perform well in producing accurate chemical reanalysis (*46*, *47*).

To assimilate AI data, an observational operator that converts the model simulation to AI was adapted from the Santa Barbara DISORT Atmospheric Radiative Transfer (SBDART) model (*48*) and added to the default WRF-Chem/DART. The SBDART has been shown to produce realistic AI values (*49*). The conversion process, as delineated by Buchard *et al*. (*50*) and Hammer *et al*. (*51*), assumes Lambertian surface albedo. Only AI values over the ocean within ±2 hours of the analysis times were assimilated, given the comparatively simpler nature of ocean surface albedo (*52*). The assimilation of AI provides important information for the final chemistry reanalysis by constraining the aerosol concentration and distribution in the smoke plume. More information about WRF-Chem/DART is available in section S1.2.

### Satellite data (SAGE III-ISS, CALIOP, MODIS, TROPOMI, and ACE)

The SAGE III (*53*) instrument is mounted on the ISS and measures the attenuation of solar radiation by atmospheric constituents during sunset or sunrise. This allows the instrument to retrieve accurate but sparse aerosol extinction coefficient vertical profiles up to 45-km altitude at different wavelengths. SAGE III-ISS data used here are the aerosol extinction coefficient at 1020 nm from level 2 solar event version 5.10.

The CALIOP data of level 2 version 4.2 were adopted to provide 532-nm aerosol extinction coefficient profiles and feature classification types. Note that the product tends to misclassify thick aerosol layers as clouds and layers blocked by strong extinction above as clean air (*54*). Therefore, the data could only indicate a rough outline of the smoke plume during the very early stage and was not assimilated by WRF-Chem/DART. In our study, CALIOP data are used to check whether the vortex identified from the MERRA2 reanalysis contains smoke aerosols.

Moderate Resolution Imaging Spectroradiometer (MODIS) AOD level 2 retrievals from both Terra and Aqua were used to constrain the model simulation by data assimilation. During the simulations, WRF-Chem/DART only assimilated the Dark Target (DT) dataset in Collection 6.1 at 550 nm with a QA flag of 3 and cloud fraction of less than 5% [see, e.g., (*55*, *56*) for more details]. MODIS AOD within ±2 hours of the analysis times was assigned eligible for assimilation. Observational errors for data assimilation were assigned following Hyer *et al*. (*57*). Because the retrieval method for MODIS AOD also fails when encountering too thick smoke layers, the assimilation of AOD is expected to only trim the smoke at the edge.

Unlike the observations mentioned above that are usually unable to retrieve thick smoke, the TROPOspheric Monitoring Instrument (TROPOMI) AI provides ideal observations for monitoring BB smoke irrespective of its thickness. TROPOMI is a nadir-viewing shortwave spectrometer on board the Sentinel-5 Precursor mission. The instrument has spectral bands in the ultraviolet (UV; 270 to 500 nm) which are exploited to calculate the AI based on the spectral contrast between the 340- to 380-nm wavelengths (*58*). Positive AI indicates the presence of UV-absorbing aerosol, and the AI is dependent upon aerosol layer characteristics such as the aerosol optical thickness, the aerosol SSA, the aerosol layer height, and the underlying surface albedo (*59*).

The ACE Fourier transform spectrometer version 4.0 data are publicly available. Here, we used them to analyze the vertical profiles and relevant chemistry of NO, NO_2_, N_2_O_5_, N_2_O, and HNO_3_ during June and July. Unlike the version 4.1 ACE data (restricted access currently), version 4.0 data have a low number of successful retrievals below 25 km (*60*) and therefore are used here only for the middle stratosphere analysis. The ozone profile data in Fig. 6B come from Bernath *et al*. (*2*), while chlorine species are from Solomon *et al*. (*27*) where the version 4.1/4.2 data were used. More details can be found in section S1.3.

### CAM-chem scenarios

CAM-chem is an active atmosphere component of the Community Earth System Model (CESM) (*61*) in which a finite volume dynamical core (*62*) was coupled with the Model for Ozone and Related chemical Tracers with tropospheric and stratospheric chemistry (MOZART-TS1) (*63*) and the four-mode version of the Modal Aerosol Module (MAM4) (*64*). CAM-chem comprehensively represents different processes that affect aerosol properties. Aerosol properties are online coupled with the model radiation and dynamics by the Rapid Radiative Transfer Model for General circulation models (RRTMG) (*65*) which makes it possible for the simulation of SCV.

#### Initial condition of the smoke plume from WRF-Chem/DART

In this study, DART was applied to assimilate TROPOMI AI and MODIS AOD observations which constrain the WRF-Chem simulations of BC and organic carbon (OC). The resultant regional reanalysis could provide a relatively accurate distribution of the smoke plume around Australia, which then serves as part of the initial aerosol condition input for CAM-Chem. We conducted continuous 6-hour cycling with WRF-Chem/DART using the 20-member ensemble to update the aerosol concentrations. The simulation was initiated on 27 December 2019. The assimilation of observations spanned from 02:00 UTC on 29 December 2019 to 6 January 2020, encompassing the period covers the most intense pyro-convection events (*12*). Detailed configurations of WRF-Chem and DART can be found in section S1.2. After injection by pyro-convection and early-stage spreading, the BB smoke from WRF-Chem/DART reanalysis was transferred into the CAM-chem, v.6.3, to simulate the subsequent transport and evolution.

#### Base scenario

We designed the “Base” scenario to investigate the transport of smoke aerosols through self-lofting and SCVs in the Australian wildfires.

We have taken the following measures to better represent the initial injection of the smoke plume. The CAM-chem model simulations started in October 2019. From 29 December 2019, OC and BC concentration fields from WRF-Chem/DART reanalysis were re-gridded and interpolated to replace corresponding CAM-chem grids. The replacement was executed daily at 02 UTC directly after the assimilation until 6 January 2020. The reanalysis introduced a smoke plume with an SSA of 0.85 to 0.90 into CAM-Chem. After 6 January 2020, CAM-chem simulations proceeded without further input from WRF-Chem/DART. Additional configurations for CAM-chem can be found in section S1.4.

We have taken the following measures to allow a free evolution of SCV while assimilating reanalysis data. During the CAM-Chem simulations, the model worked on a “specified dynamic” mode in which the model meteorology (including horizontal winds, surface pressure, and temperature) was nudged toward MERRA2 reanalysis by replacing the model calculated meteorological field by a weighted average of reanalysis and model calculations at each time step. In this scenario, the nudging process was performed in a way that model meteorological fields were not influenced by the reanalysis near the SCV. This is to guarantee that the SCV was freely simulated, while the background dynamics was close to reality.

#### NoVortex scenario

To better illustrate the impact of the SCVs, we designed the NoVortex scenario as a control case. In this scenario, the vortex formation was suppressed by replacing the near-SCV meteorological fields with the moving average from MERRA2 reanalysis in the nudging process. The moving average is conducted by meridionally averaging the zonal wind of the original reanalysis over a 40-grid moving window and the same process is applied to meridional winds and temperature fields except averaging zonally. As a result, all three SCVs from the ANYSO event were suppressed, and NoVortex simulations provided the results with the self-lofting effect but without the SCV effect. The difference between Base and NoVortex then represents the contribution of SCV effects.

#### NoFire scenario

To better illustrate the impact of wildfires, we designed the NoFire scenario as a control case. In this scenario, the 2019-2020 Australian wildfire events were removed from the model simulations. This is achieved by removing the wildfire emissions and by stopping incorporating the WRF-Chem/DART aerosol reanalysis. The difference between the Base and NoFire scenarios represents the overall impact of the ANYSO event during the 2019-2020 Australian mega-bushfire.

#### SCV + Chem and SCV scenarios

We designed the SCV + Chem scenario to evaluate the influence of BB aerosols on stratospheric ozone chemistry. Here, CAM-Chem was modified to include the heterogeneous reactions of stratospheric sulfate and organic aerosols (OA) in analogy to Solomon *et al*. (*27*). The change of HCl solubility caused by OA was considered in the same way as Solomon *et al*. (*27*) except that the calculated HCl solubility is scaled by 25% to achieve better agreement with observed anomalies (Fig. 5). The surface area density was derived from simulated OA mass concentrations and prescribed size distribution (*66*). To further improve the representation of aerosol loading and associated chemistry in the middle and lower stratosphere, we constrain the aerosol concentration in these simulations by nudging the simulated aerosol concentration toward the vertical aerosol extinction profile observed by the SAGE satellite from March to July. In detail, at monthly intervals during the model simulation, the averaged difference between the simulated and SAGE-observed aerosol profiles is computed. The model’s aerosol concentration is then adjusted according to this difference.

The difference between the SCV + Chem scenario and the NoFire scenario represents the combined effect of heterogeneous chemistry and the injection of ozone-poor air and related changes in heating and dynamics. By turning off the heterogeneous chemistry from wildfire aerosols, we then have the SCV scenario. The difference between the SCV scenario and the NoFire scenario represents the effect of the injection of ozone-poor air and related changes in heating and dynamics. Besides, we have also performed simulations without constraining aerosols from SAGE. For these unconstraint simulations, we marked them by an asterisk (*) as “SCV + Chem*” and “SCV*” to avoid confusion. More details about the model simulations can be found in section S1.4.

## Supplementary Materials

### This PDF file includes:

Supplementary Text Figs. S1 to S26 Tables S1 to S3 References
