# Supplementary material for: Smoke-charged vortex doubles hemispheric aerosol in the middle stratosphere and buffers ozone depletion
Source: Sci Adv. 2024 Jul 12;10(28):eadn3657. doi: 10.1126/sciadv.adn3657 (PMC11244531; doi:10.1126/sciadv.adn3657)
Supplement: Supplementary file 1 — Supplementary Text Figs. S1 to S26 Tables S1 to S3 References [file sciadv.adn3657_sm.pdf]

Science Advances
AAAS

Supplementary Materials for

Smoke-charged vortex doubles hemispheric aerosol in the middle stratosphere and buffers ozone depletion

Chaoqun Ma *et al.*

Corresponding author: Yafang Cheng, yafang.cheng@mpic.de; Hang Su, suhang@mail.iap.ac.cn

Sci. Adv. **10**, eadn3657 (2024)
DOI: 10.1126/sciadv.adn3657

This PDF file includes:

Supplementary Text
Figs. S1 to S26
Tables S1 to S3
References

Supplementary Text

1. Supplementary information for observations and model configurations

1.1 Three smoke-charged vortices identified from Reanalysis data

As mentioned in the main text, MERRA2 was used to identify smoke-charged vortices (SCVs) by checking the potential vorticity (PV) field, searching for consecutive vortex-like PV anomalies (compact positive anomaly in the southern and negative in the northern hemisphere with size around 1000 km) in the extratropical stratosphere. CALIOP aerosol extinction observations will also be used to check if the vortex identified from the MERRA2 reanalysis contains smoke aerosols.

The MERRA2 reanalysis data tells a detailed story about 3 SCVs from the Australian New Year Super Outbreak (ANYSO) event (*5*).The SCV that rose to 35 km and strongly influenced the middle stratosphere, as discussed in the main text, is the strongest one associated with the 30 December 2019 pyrocumulonimbus (PyroCb) outbreaks (named "P1" by Kablick III et al. (*12*) or "Koobor" by Lestrelin et al. (*22*)). It could be clearly and consecutively identified from 4 January 2020 at around 139 hPa above South Pacific Ocean to around 8 March 2020 at 12 hPa above Chile by tracking the PV field. It then disappeared in the reanalysis because of shear-induced breaking (*11*) but reformed after 13 March. Another two SCVs are much weaker but could also be identified from the MERRA2 reanalysis, also showing that such conditions are not rare anomalies. One also originated from the 30 December PyroCbs and meandered above the Antarctic (named "P2" by Kablick III et al. (*12*) or "2nd Vortex" by Lestrelin et al. (*22*)). The other originated from the 04 January PyroCbs and travelled eastward across the Atlantic (named "P3" by Kablick III et al. (*12*) or "3rd Vortex" by Lestrelin et al. (*22*)). In fact, all the three SCVs appeared in our simulation, although the formation P2 (Fig. S2A) and P3 (Fig. S9C) are more likely to be a result of meteorological nudging since the nudging window only took care of P1 in Base.

To track the stratospheric ozone (O_3) anomaly, which is another feature of SCVs (*11*), we used the ERA5 reanalysis (*67*) which is the last-generation global atmospheric reanalysis from the European Centre for Medium-Range Weather Forecasts (ECMWF). The data is based on the ECMWF Integrated Forecast System (IFS) which includes a simplified representation of O_3 chemistry (through including the chemistry that causes the O_3 hole). Together with the help of assimilating O_3-related satellite observations, ERA5 exhibits a relatively realistic spatial distribution of stratospheric O_3. For our study, the re-gridded 0.25° x 0.25° version of ERA5 hourly data was extracted whenever a candidate was identified from MERRA2 the Ertel PV field and checked to find a corresponding O_3 minimum.

1.2 Detailed configurations for WRF-Chem/DART

a. WRF-Chem configurations

WRF-Chem Version 4.1.1 was implemented with a domain that covers southeastern Australia and New Zealand (Fig. S20A). The horizontal resolution is 15 km, and the number of vertical layers was increased to 43 by adding more layers near the tropopause. The model top is around 29 km altitude. Apart from anthropogenic and biogenic emissions, the model also simulates biomass burning (BB) emissions from the Global Fire Emissions Database (GFED4s) (*68*). To include the pyro-convection process, a 1-D smoke plume rise model was applied at each model grid which calculates the plume injection height from the background meteorological profile and fire intensity (*69, 70*).

Considering that the BB emissions appeared underestimated by a factor of 3 to 4 over Australia (*71, 72*), and aerosol amount in high altitude might be underestimated because only a fixed

empirical ratio of 50% BB emission is lifted by pyro-convection in WRF-Chem, the predicted amount of BB smoke carried up by pyro-convection might be underestimated by a factor of 6 to 8 in the worst case. To account for this potential underestimation, the GFED4s source was multiplied by a factor of 7 before input into the WRF-Chem model.

The mass absorption cross section (MAC) (*73*) after several days following the BB emissions, defined here as the total light absorption at 550 nm per mass of black carbon (BC), is lower in WRF-Chem (around 8.9 m^2 g^{-1} at 550 nm) than CAM-Chem (around 14.6 m^2 g^{-1}). This means the same mass of BC absorbs less light in WRF-Chem than in CAM-Chem, although both models are assuming internal mixture of different aerosol compositions in a certain size range (*47, 64*). The discrepancy might be caused by different mass and size distribution of BC and other aerosol compositions in the two models. To be consistent with CAM-chem, the mass of BC was scaled by 1.64 in WRF-Chem only when calculating optical properties.

b. Ensemble Generation

A 20-member ensemble of WRF-Chem runs were generated by randomly perturbed the inputs and model parameters described below. At first, BB emissions were randomly perturbed with a 50% standard deviation to generate the emission ensemble. In addition, the emission factors for BC from temperate forest fires were perturbed to range from 0.56 to 0.75 g kg^{-1} for different ensemble members, estimated by Akagi et al. (*74*). To prevent deviation from the real meteorological condition (WRF-Chem /DART did not assimilate meteorological observations) and to represent the uncertainty from meteorological fields, an ensemble of meteorological fields was generated by perturbing the FNL reanalysis with WRFDA tool and overwrote the meteorological variables of WRF-Chem simulations every 6 hours at the beginning of each data assimilation cycle.

Another important uncertainty which needs to be accounted comes from the injection height predicted by the model. According to Paugam et al. (*75*), pyro-convection systems are often weakly represented in large-scale atmospheric models which do not yet provide quantitative agreement with observed injection heights. In our case, such disagreement appeared to be constant underestimation of injection height for the most important pyro-convections on 30 December 2019. Although many parameters in the plume rise model could contain error to cause the underestimation, we accounted for the uncertainty by simply perturbing the entrainment intensity in different ensemble members. Entrainment intensity was chosen only for its simplicity and effectiveness and a perturbation magnitude of 60% was chosen by try-and-error to make the ensemble maximum injection height agree with the observations (https://pyrocb.ssec.wisc.edu/). Fortunately, the assimilation of aerosol index observations to some extent corrects the smoke vertical distribution because smoke at different levels would experience different transport paths and appear at different pixels of the satellite image. For perfect solution concerning the injection height, accurate model for plume rise (*16, 76, 77*) deserves more attention in future studies.

c. Data Assimilation Configuration

In this study, DART was applied to assimilate TROPOMI aerosol index (AI) and MODIS aerosol optical depth (AOD) observations which could constrain the WRF-Chem simulations of BC and organic carbon (OC) to provide a relatively realistic smoke aerosol distribution in the early stage. The configuration of DART is similar to Ma et al. (*47*). Localization using Gaspari and Cohn function was applied with localization half width 0.05 radians (318.6 km) at horizontal and a 0.075 scale height (approximately 0.65 km at 298 K) at vertical. Covariance inflation was disabled because the smoke plumes usually leave the WRF-Chem domain within two days during which assimilated observations are unable to cause filter divergence. DART did not assimilate

meteorological variables. We conducted continuous 6-h cycling with WRF-Chem/DART using the 20-member ensemble to update the aerosol concentrations. The cycle times were 02:00, 08:00, 14:00 and 20:00 UTC, because 02:00 UTC is the local noon when most sun-synchronous satellites pass the domain.

1.3 More information concerning satellite data (TROPOMI, MLS, OMPS)

When assimilating TROPOMI AI, we only assimilated data points above ocean. Because of the difficulties in calculating the surface albedo, AI values over land were not assimilated, which is expected to have little impact given that the model domain is mainly oceanic. Observational errors for data assimilation were extracted from the uncertainty indicated with the AI dataset. The assimilation of AI provides important information for the final chemistry reanalysis by constraining the aerosol concentration and distribution in the smoke plume.

We used the Microwave Limb Sounder (MLS) Level 2, version 5 O_3 product to analyze the O_3 anomalies in Fig. 6 and Sect. S2.8. Because the original MLS O_3 data is in volume mixing ratio unit and at pressure level, similar MLS products for geopotential height and temperature were also downloaded to convert the O_3 mixing ratio into number density unit which were then interpolated to altitude levels.

The Ozone Mapping and Profiling Suite (OMPS) on board Suomi NPP satellite measures the global distribution ozone and aerosol extinction vertical profiles on a daily basis. We downloaded the V2.5 data from the Limb Profiler VIS algorithm to get the O3 density profiles from cloud top to 37.5 km. We also downloaded the V2.1 data from the Limb Profiler for 675 nm aerosol extinction coefficient profiles from the lower stratosphere (10-15 km) to the upper stratosphere (55 km). OMPS O_3 profiles have been shown to agree well correlative MLS and ACE-FTS data although limited biases exist for different sampling and retrieval methods adopted by the 3 instruments (*78*).

1.4 CAM-chem simulation scenarios

a. Supplementary information for CAM-chem configurations

After injection by pyro-convection and early-stage spreading, the BB smoke from WRF-Chem/DART reanalysis was transferred into the CAM-chem to simulate the subsequent transport and evolution. The CAM-chem model was started in October 2019. From 29 December 2019, OC and BC concentration fields from WRF-Chem/DART reanalysis at 15 km x 15 km horizontal resolution were re-gridded to 105 km x 105 km and interpolated to replace corresponding CAM-chem grids which have a horizontal resolution of 0.94° x 1.25° with 56 vertical levels up to ~45 km altitude.

Noted that, BB also emits species other than BC and OC (*79-84*). For example, halogen species emitted by BB may influence stratospheric chemistry. However, the amount of halogens from BB that could reach the stratosphere is estimated to be much smaller than the background concentrations and are thus not considered. For the same reason, direct emissions of halogen species from BB were not included in previous, related studies (*18, 19, 85*).

To be consistent with the WRF-Chem model, the light absorption by OC in CAM-chem was removed which means brown carbon was not taken into account considering its short lifetime (*86-88*). Apart from the BC and OC inherited from WRF-Chem/DART, CAM-Chem also included anthropogenic emission from the Community Emissions Data System (CEDS) (*89*) and BB emissions provided by GFED4s. BB emission was set to zero for grids corresponding to the WRF-Chem domain between 00 UTC on 29 December 2019 and 6 January 2020 to avoid double-counting. The two inventories both emitted pollutants into the first model layer without considering

plume rise. The model lower boundary conditions of ocean and sea ice were prescribed with the merged Hadley-OI sea surface temperature and sea ice concentration data (*90*).

b. Nudge the CAM-chem towards MERRA2 reanalysis

As described in the Materials and Methods, the model meteorology (including horizontal winds, surface pressure, and temperature) was nudged towards MERRA2 reanalysis in a way different than the default. The simulated temperature was not adjusted towards the reanalysis data at model layers above 312 hPa after 29 December 2019. By constraining temperature field in troposphere only, this measure could not only prevent the reanalysis from influencing the BC heating (*21*) effect in stratosphere, but also maintain a thermal structure close to reality across all model levels during long time integration.

Because the simulated SCV cannot coincide identically with the reanalysis, the simulated vortex would be weakened, and an anomalous vortex would be forced without any smoke inside at the position of the vortex if the model dynamics would be nudged everywhere. Therefore, for the "Base" simulation, simulated winds at grids within two circular windows were not nudged to reanalyses after 3 January 2020 (when the SCV could be identified in the MERRA2 reanalysis Ertel PV field). The center of the first window tracks the center of the simulated SCV and the second is centered at the reanalysis SCV (the SCV center is defined as the grid with maximum Lait PV (LPV) among a compact LPV anomaly). The radius of the windows is around 3000 km. Fig. S2C shows the distribution of nudging weights on 17 January 2020 as an example. To guarantee that the simulated SCV drifted along the same horizontal track as in the reanalysis, simulated winds within the windows were weakly nudged to the reanalysis data with weights linearly decreasing to zero from the edge to the center after 18 January and before 6 March. Fig. S2H shows the distribution of nudging weights on 29 January 2020 as an example. After 6 March, the nudging was again set zero near the SCV to make sure that the dissipation process was not forced by the reanalysis. All the measures above only focus on the SCV P1 while the other two (P2 and P3) were implicitly considered in the nudging process by simply following the reanalysis. This "Base" scenario has both self-lofting and the SCV effects and provides most results for the main text.

To simulate a scenario with only self-lofting and no SCVs (NoVortex), simulated winds were strictly nudged towards a modified MERRA2 reanalysis field for model grids near SCVs. In this modified MERRA2 reanalysis, the SCVs (from P1 to P3) were forced to disappear between 4 and 31 January 2020 by replacing zonal and meridional winds inside a circular window tracking the center of reanalysis SCVs with moving-averaged winds. The moving-average is conducted by meridionally averaging the zonal wind of the original reanalysis over a 40-grid moving window and the same process applied to meridional winds and temperature fields except averaging zonally. Fig. S2A and S2D show an example of the Ertel PV fields before and after modification and Fig. S2E shows the distribution of nudging weights on 17 January 2020. With the help of the modified reanalysis wind and the nudging process, the simulated vortex is constantly damped to prevent its formation. After 31 January 2020 the nudging weight was again set zero near the SCV. Readers should also be careful that the modifications of reanalysis not only influence the SCVs but also slightly change the transport of the rest part of plume. This caused the differences in total stratospheric aerosol mass between NoVortex and Base in Fig. 4B but the differences are fortunately limited considering the maximum mass entering the stratosphere. The differences between Base and NoVortex could be approximately considered as the contribution by SCV effects.

c. Other simulation scenarios

To disentangle the impacts of pyro-convection at 30 December 2019 and 4 January 2020 (*5, 91*), another two simulations were carried out similar to Base. One had no WRF-Chem/DART replacement before 4 January 2020 (Base_Only04) and the other had no WRF-Chem/DART replacement after 4 January 2020 (Base_Only30). To understand the effect of BC heating in the formation of SCV and the self-lofting of the plume, a simulation was implemented like Base except that the BC absorption of sunlight was switched off (NoAbs). To show the role of BC heating in maintaining the SCV, a simulation (NoAbs_After10) followed exactly the same as Base run before 10 January but BC did not absorb solar energy after 10 January when the SCV matured.

2. Supplementary Discussions

2.1 Reproducing a SCV in the model

Model simulations of the Australian fire event (*18, 19*) have not captured the SCV and hence greatly underestimated the plume rise (only up to ~25 km).

Difficulties of reproducing SCVs in model simulations also appeared in studies on the 2017 Canadian wildfires (*14, 21*). The CALIOP profile in Fig. S1 clearly crosses a negative vortex-like PV anomaly. This SCV was named "Vortex A" by Lestrelin et al. (*22*) and was distinctly documented to be associated with the 2017 Canadian wildfires. However, Das et al. (*21*) simulated the plume top to be at around 20.5 km altitude at the same time and position (shown in their Figure 7) and Yu et al. (*14*) located it below 19 km altitude in their best-estimate simulation (shown in their Fig. S2A).

Noted that, the plume top height has been defined rather arbitrarily in publications, which might lead to differences in the reported results. For example, Yu et al. (*14*) defined the plume top as the maximum height with aerosol backscatter coefficient at 532 nm greater than $3x10^{-4}$ km^{-1} sr^{-1}, while Das et al. (*21*) used the maximum level at which the mean aerosol extinction is greater than an unknown "background extinction". In our results, the plume top height is defined as the maximum level at which the $PM_{2.5}$ concentration is 50 times higher than the $PM_{2.5}$ vertical profile averaged over the extratropical south hemisphere (ESH, which is defined between 25°S and 90°S here) from the NoFire simulation.

These issues also played a role in Doglioni et al. (*20*) who implemented the Goddard Earth Observing System (GEOS) model (*92*) in a free running mode with 0.3 Tg carbonaceous aerosol injected at times and locations according to observations. Although an SCV was successfully generated, it differed from the observations (*22*) in the following aspects. (i) Only one SCV was simulated while the observations showed one SCV split into several offspring. (ii) The track of the simulated SCV did not follow observed SCV or its offspring: all the observed ones remained north of 30 °N while the simulated one spent most of its life cycle south of 30 °N. (iii) The simulated SCV only lasted for 25 days while the observed SCV and offspring lasted a total of two months. (iv) The simulated SCV was larger than in the observations, with aradius of 700 km in contrast to the observed 350 km (main SCV). (v) The simulated SCV ascended too rapidly; it reached the 570 K potential temperature level almost 30 days earlier than in the observations.

The discrepancy between the simulations and observations, especially the SCV track and size, hinders the understanding of the lifecycle of such events and their impact on the aerosol budget and O_3 layer in the middle stratosphere.

By comparing our simulation setup with previous studies, and according to our experience, the difficulty of reproducing a SCV in a model is mainly related to: (a) How to inject the correct smoke

amount into the model at the observed position. The initial state of the smoke not only determine the rise potential for the whole plume (*93, 94*), but also the formation of the SCV. Because in our case the SCV was formed by the part of the plume that encountered a preexisting PV anomaly (Sect. S2.9), the initial state of the smoke injected determines the plume encounter of the anomaly, the entrained amount of aerosol and the intensity of the formed SCV. We solved this by assimilating satellite observations (See Materials and Methods and Sect. S1.2). (b) How to balance the reanalysis data nudging with the model dynamics. Because nudging from reanalysis dampens the SCV forced by BC heating in the model, we preferred to refrain from nudging as also noticed by Doglioni et al. (*20*). However, reanalysis data nudging is also necessary for the model to realistically simulate the background meteorology, which determines the appearance of the preexisting PV anomaly as well as the trajectory and lifetime of the SCV. Our proposed solution is to set up a window tracking in the SCV with low nudging within and normal nudging outside (See Materials and Methods and Sect. S1.4).

2.2 Simulated smoke plume vs. multiple observations

The WRF-Chem/DART reanalysis has been compared with TROPOMI AI (Fig. S20A and S20B) and CALIOP aerosol extinction coefficient profiles (Fig. S20C and S20D). The reanalyzed AI at 1 January is similar to the observation with the same horizontal position, shape and maximum AI value around 25 which indicates that the observed aerosol absorption is reproduced well (details in Sect. S2.5). Because TROMOPI AI was assimilated, such similarity is expected. The aerosol extinction coefficient curtains indicate the vertical position of the smoke. Although the main body of the smoke plume is misclassified by the CALIOP product as "cloud" and no signal (blank data pixels) for too high extinction (Fig. S20D), the data are nevertheless helpful to estimate the vertical position of the smoke which extends to around 17 km altitude, being reproduced by the simulation.

The main smoke plume from the Base simulation has been compared with corresponding observations from CALIOP (Fig. S3), a ground-based lidar (*26*) (Fig. S4A) and a spectral sun photometer of the AErosol RObotic NETwork (AERONET) (*95*) (Fig. S4B). The simulated aerosol has the same spatial distribution as the CALIOP observations, and the magnitude of the extinction coefficient is also mostly similar between the two. In some instances (21:03 UTC Jan 10 for example), the simulated extinction is lower than the observed one by around 50%. A similar underestimation appeared from the comparison with ground-based observations. Although the simulated aerosol extinction reaches a maximum at the same altitude (at 15 km) as measured by the ground-based lidar, the simulated maximum value is only 0.03 km^{-1} while the observed is around 0.06 km^{-1}. Similarly, the AOD from both the AERONET observation and simulation reached a maximum on 9 January and dropped quickly on 10 January, but the observed maximum is around 0.3 at 550 nm, higher than the simulated 0.2. The above underestimation does not necessarily mean that the aerosol mass was simulated too low but might rather be caused by the coarse spatial resolution of CAM-Chem that could not resolve the strong concentration gradient. This is supported by the fact that the simulated extinction profile does not underestimate the maximum value if sampled 300 km north of the ground-based lidar (red line in Fig. S4A).

It is worth noting that the CALIOP track at 21:03 UTC Jan 10 shows two smoke plume fragments at similar altitude with one in the stratosphere and the other in the troposphere. This track is in the "far-field" which is assumed to not contain tropospheric smoke when estimating the stratospheric smoke aerosol mass in Hirsch et al. (*96*). Therefore, they might have overestimated the stratospheric mass by including part of the tropospheric mass, which explains why their final estimation of 2.1±1.0 Tg is higher than ours. For our estimation, as shown in Fig. S15A, the Base case simulated a maximum increase of 0.8 Tg for the total stratospheric mass compared to the NoFire case in the middle of January.

Vertical profiles of the aerosol extinction coefficient from SAGE III-ISS observations have been integrated between 10 to 30 km altitude to represent the AOD contributed by the main smoke plume. By comparing the AODs sampled over the ESH between observations and the corresponding Base simulations, it is possible to reduce the influence of the limited model resolution and spatial pattern mismatch and verify if the total simulated smoke amount is biased. In general, the comparison (Fig. S5) indicates little bias between the simulations and observations during the first 3 months. The spread of AODs from both observations and simulations is relatively large in January and February because the smoke had not mixed across the ESH yet. After April, the simulated AOD was generally somewhat lower than in the observations, indicating that the model removed the stratospheric aerosol more rapidly than in reality.

2.3 Simulated SCV vs. CALIOP observations

Next to the main smoke plume, the simulated aerosol extinction within the SCV has also been compared with available CALIOP observations, sampled across the SCV. Because the track of the SCV is not fully identical between simulations and observations, the model results were sampled at the same time but at a position slightly displaced to cross the point where the maximum $PM_{2.5}$ concentration was reached. For all 11 cases from January to February shown in Fig. S12 and S21, the observed aerosol bubble resided at a similar altitude as in the model. The CALIOP observations suggests higher extinction than the simulations, but the signal is rather noisy with an irregular distribution, indicating high uncertainty from the retrieval process. By averaging the extinction curtain along the sampling track, the simulated extinction is within the uncertainty range for most cases. When considering the 11 cases together (Fig. S22), the observed average extinction was 1-2 times that in the simulation, up to a factor of 3 in extreme cases. Considering the similar magnitude of the temperature gradient shown in Fig. S7, the thermal properties of the SCV are simulated realistically. However, the above-mentioned underestimation could still introduce error when evaluating the aerosol impact on middle-stratospheric O_3, which justifies the adjustment of aerosol applied in simulating the O_3-related heterogeneous reactions (See Materials and Methods and Sect. S2.7).

2.4 The QGPV equation and different kinds of potential vorticity

Under quasi-geostrophic condition, the behavior of the atmosphere could be described by the Quasi-geostrophic PV (QGPV) equation (*25*),

$$\frac{D_g}{Dt}q = \frac{D_g}{Dt}\left[\frac{1}{f_0}\nabla^2\Phi + f + \frac{\partial}{\partial p}\left(\frac{f_0}{\sigma}\frac{\partial\Phi}{\partial p}\right)\right] = -f_0\frac{\partial}{\partial p}\left(\frac{\kappa J}{\sigma p}\right). \quad (1)$$

q is the QGPV which is the sum of geostrophic vorticity ($\frac{1}{f_0}\nabla^2\Phi$), Coriolis parameter or planetary vorticity (f) and temperature stratification ($\frac{\partial}{\partial p}\left(\frac{f_0}{\sigma}\frac{\partial\Phi}{\partial p}\right) \approx -\frac{Rf_0}{p\sigma}\frac{\partial T}{\partial p}$). In the QGPV equation Φ is the geopotential, p is the air pressure and J denotes the diabatic heating rate that mainly comes from absorption of solar radiation and radiative cooling in our study. κ is defined as $\frac{R}{c_p}$ where R is the gas constant and c_p represents the specific heat of dry air at constant pressure. σ denotes $-\frac{RT_0}{p}\frac{d\theta_0}{dp}$ in which T_0 and θ_0 are the basic state temperature and potential temperature respectively. Both σ and κ are positive. f is the Coriolis parameter whose linearized approximation is f_0 and they are positive (negative) in the northern (southern) hemisphere. $\frac{D_g}{Dt} = \frac{\partial}{\partial t} + u_g\frac{\partial}{\partial x} + v_g$ and means the change rate following the geostrophic wind (u_g and v_g).

For the southern hemisphere, a positive diabatic heating effect (DHE) ($-f_0 \frac{\partial}{\partial p}\left(\frac{\kappa J}{\sigma p}\right)$)) happens when J increases with p (J decreases with increasing altitude), and means an increase of q which leads to a positive anomaly of $\frac{1}{f_0}\nabla^2\Phi$ and $-\frac{Rf_0}{p\sigma}\frac{\partial T}{\partial p}$. In the southern hemisphere, positive relative vorticity means counterclockwise or anticyclonic vorticity. In simulations in which BC did not absorb solar energy (NoAbs) (Fig. S9E and Fig. S15D), no signal of the SCV was captured and much less stratospheric aerosol remained. BC heating is therefore necessary in generating the SCV besides maintaining the high aerosol loading in the lower stratosphere.

The absence of diabatic heating means the conservation of QGPV along geostrophic wind under quasi-geostrophic conditions. It does not mean that a SCV, once formed, could be maintained forever even without the BC heating. This is because the QGPV equation does not consider ageostrophic dissipation processes. In a simulation in which BC did not absorb solar energy after the SCV became mature (NoAbs_After10) (Fig. S23), the SCV rapidly disappeared in the next 10 days.

Further, the temperature needs to decrease with increasing altitude to make $-\frac{Rf_0}{p\sigma}\frac{\partial T}{\partial p}$ positive. The temperature perturbation is also replicated by the Base simulation as shown in Fig. S7, which corresponds well with the observations shown in Fig. 5d of Khaykin et al. (*11*) (reprinted as Fig. S7B here).

Without the limitation of quasi-geostrophic conditions, a more general form of PV is the Ertel PV defined as

$$Ertel\ PV = \frac{\overrightarrow{\boldsymbol{\omega}_a}}{\rho}\cdot\nabla\theta, \tag{2}$$

in which $\overrightarrow{\boldsymbol{\omega}_a}$ is the absolute vorticity vector, which is the sum of the relative and planetary vorticity, ρ is the air density and θ is the potential temperature. Ertel PV is also conserved in frictionless air without diabatic heating.

LPV is defined as $(Ertel\ PV)\frac{\theta}{420K}^{-4}$ following Lestrelin et al. (*22*) to reduce the background vertical gradient of Ertel PV.

The QGPV equation is chosen instead of the often-used Ertel PV because it is more straightforward to illustrate the mechanisms. In fact, QGPV, LPV and Ertel PV are approximately equivalent in our study case. QGPV is proportional to a linearized form of Ertel PV (*25*) and LPV is calculated directly from Ertel PV.

To be consistent throughout the paper, if not specifically noted, the center of the aerosol bubble (or plume), SCV, and vortex are defined respectively as the grid cell with maximum $PM_{2.5}$ concentration, LPV, and relative vorticity among a compact anomaly. The compact anomaly is usually easy to identify in the map with a clear boundary distinguish itself from the background (Fig. S2). In most cases, however, the grid with maximum LPV also has the maximum relative vorticity because of the close relationship between LPV and relative vorticity.

2.5 Single scattering albedo and black carbon ratio of the smoke

The single scattering albedo (SSA) measures the absorption efficiency of smoke aerosol and therefore is critical to the rise of BB aerosol particles. In our Base simulation, SSA at 550 nm of the stratospheric smoke plume changes from 0.866 (±0.013) at beginning of January to about 0.898 (±0.009) in the middle of January and then keeps the same value during the remaining simulation period. The increase of SSA results from the change in microphysical properties when BC and

organic aerosol (OA) from BB transfer from the primary into the accumulation mode in the model (*64*).

The SSA estimated from simulations is relatively reliable especially at the beginning of January for the following two reasons. Firstly, the aerosol concentration of the CAM-Chem model comes from the WRF-Chem/DART reanalysis during the early stage. The reanalysis is constrained by assimilating AI observations which depend on the total amount of aerosol and is extremely sensitive to the SSA. Given that the reanalysis AI is close to the observations (Fig. S20A and 20B) and the simulated AOD of smoke shows little bias (Fig. S5), it is reasonable to assume that the simulated SSA is close to reality. Secondly, the SSA of stratospheric BB smoke could be retrieved from remote sensing observations. For the 2017 Canadian wildfire event, smoke across the Atlantic was observed by several ground-based lidars which gave estimations of 532 nm SSA as 0.75-0.85 from Haarig et al. (*97*), 0.82-0.89 and 0.86-0.90 from Hu et al. (*98*). For the 2019/2020 Australian case, SSA was estimated to be 0.74-0.84 at 532 nm according to ground-based lidar by Ohneiser et al. (*99*) and 0.25-0.7 at visible wavelength according to AOD and radiative flux observation by Hirsch et al. (*96*). The estimation process of Hirsch et al. (*96*) contains an error by substituting a positive number as the radiative forcing which in fact should be negative. After correction, the estimate should be 0.33-0.92. Our simulation (0.853-0.909) seems realistic when compared with the observational estimations.

The SSA depends on the relative amounts of the aerosol components (BC and OA in our case) and their microphysical properties (size distribution, morphology and mixing state etc.). In our simulation, BC accounts for around 3% of the total smoke aerosol mass. However, the BC ratio estimated here is ambiguous considering that the microphysical properties of BC could be different from reality and between different models. Large uncertainty concerning the BC microphysical properties appeared from the differences of MAC between WRF-Chem and CAM-Chem. Besides remote sensing (*100*), more in-situ sampling and analysis of stratospheric BB aerosol particles (*101*) will be needed to improve the representation of BB processes in models.

2.6 Mechanism in the absence of cyclonic vortex

During the maturation period, both negative and positive DHE existed. The positive DHE corresponds to the observed anticyclonic vortex, while no cyclonic vortex corresponds to the negative DHE, which may be confusing when trying to explain the SCV with QGPV theory in previous work (*11*). With highly detailed simulation results, we offer a possible explanation.

As shown in Fig. S18, because of the larger gradient of aerosols near the upper part of the plume, the positive DHE is stronger than the negative DHE, which leads to a net enhancement of the anticyclonic vortex. The different gradient is caused by a higher aerosol concentration and a corresponding larger buoyance at the plume center (grid with maximum $PM_{2.5}$ concentration) than at the upper and lower edge. Air masses thus rise more at the concentration center, which compresses the upper part of the plume and expands the lower part.

The difference of rising speed within the bubble may also be the reason why the negative DHE did not form a corresponding cyclonic vortex. Because the center of the anticyclonic vortex (grid with maximum relative vorticity) is located above the concentration center, it tends to move downward relative to the concentration center. Consequently, the negative DHE works to damp the preexisting positive vorticity from above and is unable to form another cyclonic vortex.

2.7 The impact of Smoke-charged vortex on ozone chemistry in the middle stratosphere

Recently, Strahan et al. (*85*) indicated that the reactions happening on BB aerosols might be different from those on sulfate, given that the repartitioning of chlorine between HCl and $ClONO_2$ were observed (*33*) but could not be modeled and explained by simply treating BB OA as sulfate. According to Solomon et al. (*27*), it might be caused by some heterogeneous reactions, which are ignorable on sulfate at midlatitude, are facilitated on smoke OA due to the higher solubility of HCl on OA than sulfate. We also considered this mechanism and implemented it in our simulations in the same way as Solomon et al. (*27*). Instead of considering solubility of HCl in organic aerosols the same as in hexanoic acid like Solomon et al. (*27*), we found that scaling HCl solubility by 25% can resolve the issue with overestimation of simulated anomalies for both O_3 and chlorine species (see "SCV+Chem (unscaled)" in Fig. 5, and Fig. 3 in Solomon et al. (*27*)). The reactions facilitated by OA could increase the amount of ClO and therefore yield more chemical O_3 depletion in the lowermost stratosphere than that the reactions on sulfate aerosols (*27*). However, this difference between OA and sulfate has limited impact in the middle stratosphere. This is because, in the middle stratosphere, catalytic reactions with NO_x rather than reactive chlorine dominate O_3 depletion (*85*).

It has to be noted that the O_3 difference shown in "SCV+Chem" does not only originate from the heterogeneous reactions mentioned above, but also from non-chemical effects like heating and dynamical changes induced by the smoke (*18*). The impact of non-chemical effects can be seen in Fig. 6A as the "SCV" and were ruled out by only considering the difference between "SCV+Chem" and "SCV". In Fig. 6A, we show the effects from heterogeneous reactions only as the dotted area, and we calculated the column O_3 changes (DU numbers in legend) by integrating the area between anomaly profiles of "SCV+Chem" and "SCV" above the tropopause.

Although in the correct direction, the modeled O_3 anomaly of "SCV+Chem*" without SAGE constraint (Fig. S16A) in middle stratosphere are underestimated compared to the observations. This mainly caused by the underestimation of aerosol amount inside SCV (discussed in Sect. S2.3) which makes the aerosol increase in middle stratosphere is low from the perspective of both amount and altitude. By continuously constraining the averaged hemispheric aerosol amount or nudging the aerosol amount exclusively around the SCV at the genesis of the SCV according to satellite observations (Fig. S16B), "SCV+Chem" in Fig. 6A or "SCV_nudge+Chem" in Fig. S16A could yield satisfactory reproduction of the O_3 increase in the middle stratosphere.

Since O_3 is also a heat source in the stratosphere mostly by absorbing solar radiation, an increase of O_3 in the middle stratosphere induces additional warming (Fig. S24) besides the direct BC heating, which deserves further study considering the complex relationship between atmospheric circulation, temperature and O_3 layer (*102, 103*). Noted that, this work focuses on the midlatitude and the situation for Antarctic O_3 was more complex in 2020 probably because of the formation of polar stratospheric cloud (*99, 104, 105*).

2.8 The contribution from latitudinal transport to observed ozone anomaly

One of the key arguments of this work is that the observed O_3 increase in middle stratosphere over south hemispheric midlatitudes in June and July is caused by the chemical reactions on the BB aerosols carried by SCV to the middle stratosphere. This conclusion is supported by similar pattern between the observed O_3 anomaly (data of year 2020 minus the average of data from 2005 to 2019) and O_3 difference between simulations with and without BB aerosols ("SCV+Chem" at year 2020). Such an idea has a deficiency that model simulations only consider the influence of BB aerosols while ignoring other natural variabilities that could cause the observed departure of O_3 from its climatological average. One of the most important natural variabilities of O_3 in mid-latitudes stratosphere comes from the latitudinal transport of the tropical O_3 variability caused by quasi-

biennial oscillation (QBO). Such a process has been noticed early back to the research of Pinatubo volcano (*106-108*). Therefore, we need to exclude the possibility that BB aerosol introduce no chemical effects and the observed positive O_3 anomaly in 2020 is only a result of natural variability caused by transportation.

a. QBO effect is not enough to explain the observed O_3 increase in middle stratosphere

According to the nature of QBO influence, its effects on O_3 should originate from tropical and then propagate towards south and north hemispheres. This means the signal of QBO should be approximately symmetrical about the equator. As shown at the altitude of 24.4 km (Fig. S25B), the impact of QBO could be easily identified from the deseasonalized O_3 anomaly as a positive anomaly starting at April within the tropical and a negative anomaly within tropics before March which then expanded towards mid-latitudes of both hemispheres after March. Similarly, at altitude 25.6 km, positive anomalies which are possible QBO signals are like the two positive anomalies at 20°S and 20°N before March as well as two weak positive anomalies at 10°S and 10°N in July. The positive O_3 anomaly in June and July over 30-60°S, which is of our interest, has neither north-hemispheric counterpart nor a propagation pathway from the equator, which makes it less like a result of QBO.

Diallo et al. (*109*) did more detailed research to quantify the impact of QBO to O_3 in 2020 by a multi-linear regression approach. Their Figures 3d and S2d tell a similar story as our analysis that QBO effect only contributes a very small part of the total O_3 anomaly above 24 km at around 50°S. We reorganized their data into a vertical profile in Fig. S26 which clearly shows the trivial contribution from QBO.

b. Observational evidence of abnormal conversion from NO_x to HNO_3 in the middle stratosphere

Although the influence of QBO is trivial, we still need more observational evidence to support that the heterogeneous reactions did happen as proposed in the main text.

At first, we checked how N_2O is correlated with NO_x+2·N_2O_5 (ozone depleting NO_x plus its temporary reservoir) and HNO_3 (long lifetime NO_x reservoir). As shown by the 2004-2019 climatology (Fig. 6C), NO_x+2·N_2O_5 and HNO_3 share the same correlation pattern with N_2O in which NO_x+2·N_2O_5 and HNO_3 increase with the increase of N_2O at N_2O lower than 5×10^{10} molecules cm^{-3} and decrease with the increase of N_2O at N_2O higher than that. Because N_2O is a rather inert trace gas in stratosphere, its variation could indicate the influence of transportation. The same correlation pattern thus means NO_x+2·N_2O_5 and HNO_3 should simultaneously increase or decrease if influenced by abnormal transportation. However, the fact of 2020 is that NO_x+2·N_2O_5 significantly decreased while HNO_3 significantly increased when compared with the climatology (Fig. 6C), which thus undermines the role of abnormal transportation in middle stratosphere.

The coincident increase (decrease) of HNO_3 (NO_x+2·N_2O_5) has a similar amount of $\sim4\times10^8$ molecules cm^{-3} (Fig. 6C). This phenomenon indicates an abnormal chemical conversion from NO_x+2·N_2O_5 to HNO_3 at 2020, which is most likely to be the result of the proposed BB aerosol heterogeneous reaction that convert N_2O_5 (which is reversibly converted from NO_2) to HNO_3 analogous to volcanic sulfate aerosol (*2*).

c. The role of chemical and transportation effects in ozone anomaly of lower stratosphere

Although it is not the key point of this study, another fact, the negative anomaly of O_3 in year 2020 over south mid-latitudes in lower stratosphere, has been widely discussed in previous

research. The decrease of O_3 was first attributed to the heterogeneous reactions on BB aerosols (*18, 19*) but later more researches claimed transportation effects played the major role(*33, 85*). We tend to think that transportation and chemistry on BB aerosols synergically contributed to the low O_3 in lower stratosphere while transportation effects might be slightly more important. The reasons are:

(1) By comparing the Figures 3d and S2d in Diallo et al. (*109*), QBO explained most of the observed O_3 anomaly in lower stratosphere (the total percent anomaly is >10% while QBO caused a percent anomaly of >7%). That is similar to our analysis in Fig. S26A in which the negative O_3 anomaly over south mid-latitudes has a counterpart in north mid-latitudes at 19.6 km altitude, indicating possible influence of QBO. However, it needs to be noted that about 1/3 of O_3 anomaly remains unexplained and might be the result of chemical effects. Our results in Fig. 6A also indicate chemical effects ("SCV+Chem" - "SCV") does not contribute all the O_3 depletion in lower stratosphere.

(2) The observed repartition between HCl and $ClONO_2$ has been undisputedly attributed to the chemical reactions on BB aerosols (*27, 33, 85*). According to Solomon et al. (*27*) who tried to simulate the repartition, the shift in chlorine chemistry itself could cause nontrivial O_3 depletion in addition to the impact from traditional N_2O_5 hydrolysis in lower stratosphere. So, the participation of chemical effects from BB aerosols could not be excluded.

(3) Santee et al. (*33*) presents several analysis and detects no signal of chemical O_3 loss. However, their methods are essentially not sensitive enough to detect the expected magnitude of signal from chemical effects on O_3.

2.9 Conditions for the occurrence of smoke-charged vortices

This section presents a hypothesis about the preconditions required to form an SCV. To form a SCV, a large amount of BB aerosol needs to be carried into the lower stratosphere, which requires strong pyro-convection (extratropical cyclone was also suspected to help during the Australian fire (*110*) and needs more research). Also, we assume that a preexisting PV anomaly (positive anomaly in the southern hemisphere and negative in the northern hemisphere) may play a role in the formation of SCVs because it could provide aerosol the initial vorticity that limits the dilution of BC and thus facilitating the feedback in the formation period of the SCV. In the extratropical lowermost stratosphere the initial PV anomaly could come from the intrusion of tropical tropospheric air (*111*) which happens to have lower (higher) PV than air at the same altitude of the extratropical northern (southern) hemisphere. Also, it could come from the air of lower altitude brought aloft by the pyro-convection (*112*).

Fig. S10 shows the simulated evolution of the PV anomaly and the smoke plume at the beginning of this event. At 118 hPa on 00:00 UTC, 30 December, no BB plume existed yet but an isolated high PV anomaly appeared between Australia and New Zealand and kept moving eastward in both Base and NoFire simulations. This anomaly might be similar to the isolated PV patches observed during subtropical wave breaking (*113*). For the Base case on 1 January, the BB plume encountered this PV anomaly when it rose to this altitude over New Zealand. After that, this PV anomaly might act as the "seed" to provide the initial vorticity that was subsequently enhanced (the right column in Fig. S10 from 2 January to 5 January) by the DHE due to BC heating, thus accelerating the feedback to form a SCV. For the NoFire case, however, this PV anomaly is weaker than that in the Base case after 3 January and finally almost disappeared on 5 January, highlighting the importance of BB aerosol in forming the SCV. It should be noted that, a preexisting PV anomaly helps the formation of the SCV but the SCV could still be formed without it in a longer time and less axisymmetric form (*112*).

2.10 Frequency of SCV events under climate change

To detect SCVs, we checked the historical MERRA2 reanalysis Ertel PV data, searching for consecutive vortex-like PV anomalies (compact positive anomaly in the southern and negative in the northern hemisphere) in the extratropical stratosphere. As shown in Table S3, 15 cases were identified after the year 2009. By checking neighboring CALIOP profiles, only 5 of them contained aerosol anomalies and might have been SCVs. Except the typical SCVs in 2017 and 2020, the case in 2009 happened at low latitudes where geostrophic effects are weak and therefore might not form a mature SCV; the interruption of CALIOP data also hindered the further tracking of this case. The aerosol signal from CALIOP for the July 2015 case was classified as a mixture of smoke, dust and stratospheric clouds; considering the short lifetime and the absence of lofting potential, it is uncertain whether the aerosols inside were from BB and contained enough BC (*114*).

The remainder of the cases in Table S3 are likely to be the isolated PV patches described in Sect. S2.9 that used to be part of a tropical air intrusion. The evidence suggests that those PV patches also appear to be negative O_3 anomalies which indicates they came from the tropics where O_3 concentrations are lower than in the extratropics. These PV patches usually have a shorter lifetime (1-3 weeks) than SCVs (2-8 weeks) and could not rise similarly.

If the theory in Sect. S2.9 is valid, the frequency of such SCVs is expected to increase in the future as a response to global warming. A tentative estimate about the trend of SCV occurrence probability in the future may be obtained by assuming the incidence of deep convection, BB events (pyro-convection was decomposed as BB event and deep convection), and PV seeds from tropical air intrusions that do not affect each other (independent events).

If the current climate warming trend persists, the incidence of deep convection is projected to increase by around 3% per decade both from the perspective of mean convective available potential energy (*35*) and precipitation extremes (*115*). It has to be noted that meteorological conditions favoring pyro-convection, although share many similarities, is not the same as normal moist convection (*116*), so the trend for normal convection here might not be accurate for pyro-convection and more investigations are needed.

Then, the intrusion of tropical air into the exratropics in the upper troposphere and the lower stratosphere (UTLS) could be interpreted as the shallow and transition branch of the Brewer-Dobson circulation or the weakening of the subtropical jet stream. The shallow and transition branch is expected to increase by ~3% per decade under anthropogenic forcing (*36*). A similar trend was detected as the subtropical jet stream weakened by about 3% per decade in the past 50 years (*117*), thus becoming increasingly conducive to intrusions.

Finally, the trend of BB activities is neither clear nor uniform in the past decades because fire activity not only depends on the climate, but is also controlled by uncertain human management (*34*). If viewed from the climate perspective, both past observations (*118-121*) and future projections (*122, 123*) indicate a trend with climate change favoring more wildfires. Although the observed fire activity decreased globally (*124*), it significantly increased in the western US (*119, 120*) during the past decades. Model predictions are also limited by model uncertainties and yield trend projections around -5% to 5% per decade globally (*34*) and -15% to 15% per decade in North America (*122*).

In the worst case, the occurrence probability of SCV could increase by about 8% per decade assuming incidence probability of deep convection and BB events increasing by 5% and 3% per decade, respectively. This means that by the end of this century, the number could double for similar SCV events and much more BB aerosol could enter the middle stratosphere. BC carried into the mid-stratosphere could reside about 1-2 years and might introduce climate impacts (*125, 126*)

considering that a BC layer at 30 km altitude causes twice the surface cooling of one at 12 km (*1, 127*). Given that most previous researches(*128-131*) calculate the radiative forcing for the 2019/20 Australian fire by taking the stratospheric smoke as a whole, it would be interesting to calculate the relative contribution from smoke in lower and middle stratosphere.

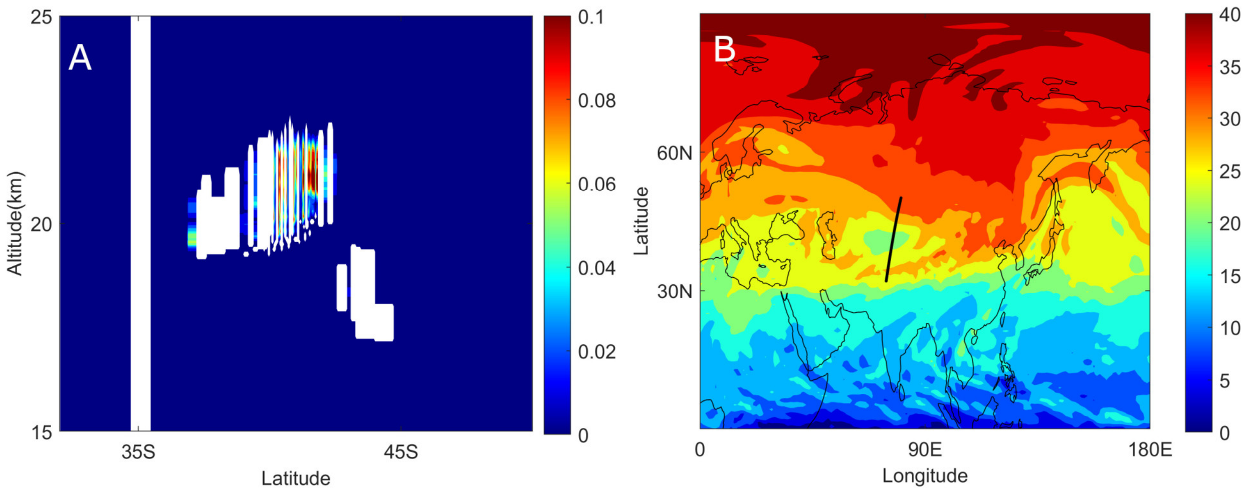

Fig. S1. Position of an SCV during the 2017 Canadian wildfire event. (**A**) Aerosol extinction coefficient (km^{-1}) curtains at 532 nm from a CALIOP track near 03 September 2017 21:00 UTC. An aerosol bubble with top higher than 22 km altitude could be identified. (**B**) Ertel PV map (PVU) at 52 hPa (~21 km altitude) from MERRA2 reanalysis of 03 September 2017 21:00 UTC. The black line denotes the CALIOP sampling trajectory of panel (**A**).

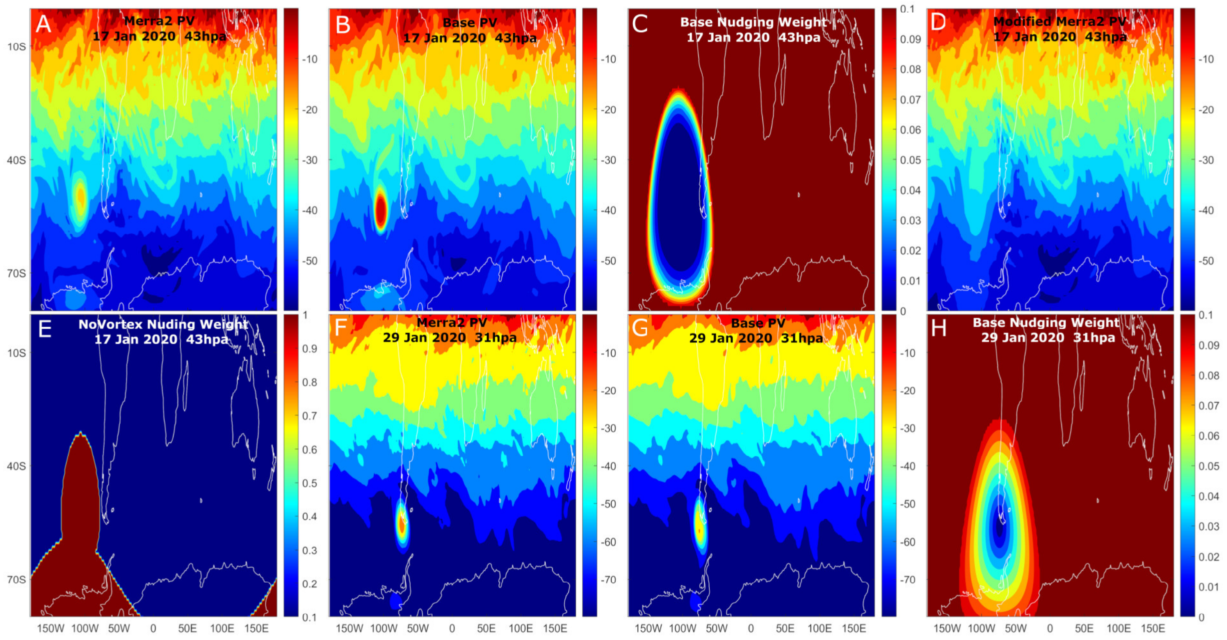

Fig. S2. Distribution of Ertel PV (PVU) and nudging weight. (**A**) Ertel PV from MERRA2 reanalysis at 43 hPa level and 00 UTC 17 January 2020; the isolated high PV patches at ~100°W above the south Pacific and Antarctic are SCV P1 and SCV P2 respectively. (**B**) Simulated Ertel PV map from Base. (**C**) Nudging weight assigned to reanalysis for Base run at 00 UTC 17 January 2020. (**D**) Ertel PV of the modified MERRA2 reanalysis used in NoVortex simulation. SCVs were zeroed out by moving average. (**E**) Nudging weight like panel (**C**) except for the NoVortex run. (**F**) Ertel PV from MERRA2 reanalysis at 31 hPa level and 00UTC 29 January 2020. (**G**) Ertel PV map like panel (**F**) but from Base simulation. (**H**) Nudging weight like panel (**C**) but at 00UTC 29 January 2020. Note that panel (**E**) uses a color scale different from (**C**) and (**H**) in that the weight outside the red region in panel (**E**) is uniformly 0.1.

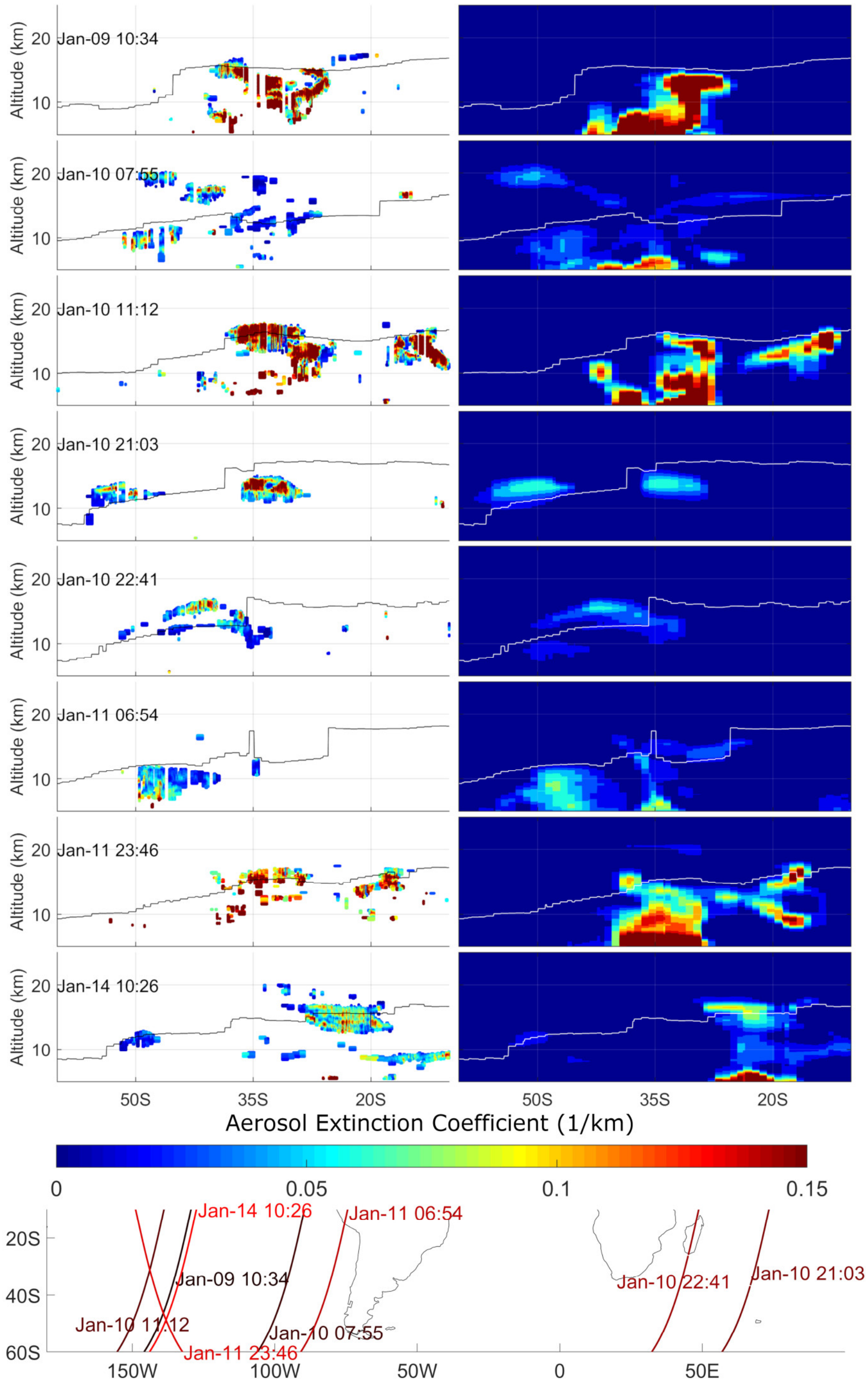

Fig. S3. Comparison of the aerosol extinction coefficient (km^{-1}) between CALIOP observation and Base simulation for plumes outside the SCV. Aerosol extinction coefficient curtains at 532 nm observed by CALIOP were plotted at the left column while corresponding data from the Base simulation at 550 nm are at the right column. Pixels classified as "Clean Air" by CALIOP were plotted as blank as other non-aerosol pixels in the left column. The black (white) lines in the left (right) column are simulated tropopause. Sampling trajectories of the CALIOP were plotted at the bottom panel.

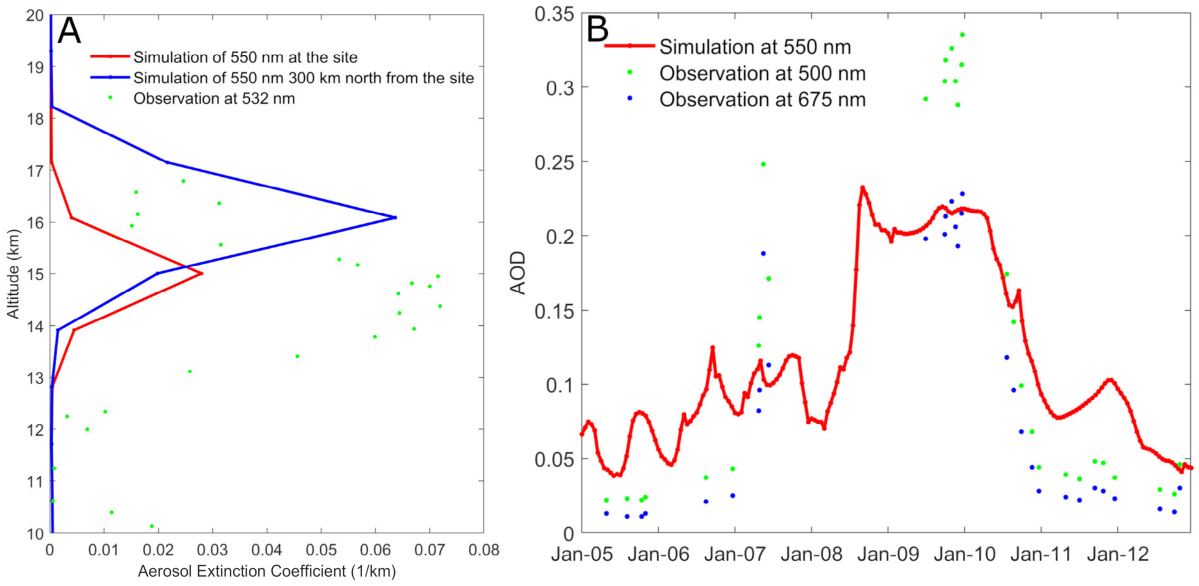

Fig. S4. Comparison of modeled and observed vertical distribution and total aerosol loading. (**A**) Comparison of aerosol extinction coefficient profile (averaged of 03:56–05:40 UTC in 9 January 2020) between a ground-based lidar observation and simulation. The ground-based lidar is located at Punta Arenas (53.2°S, 70.9°W) and data were imported into our plot by measuring pixels of Fig. 3 in Ohneiser et al. (*26*). (**B**) Comparison of AOD between observation from an AERONET station and simulation. The AERONET station is at Marambio (64.240°S, 56.625°W) of which data were imported by measuring pixels of Fig. 2 in González et al. (*95*).

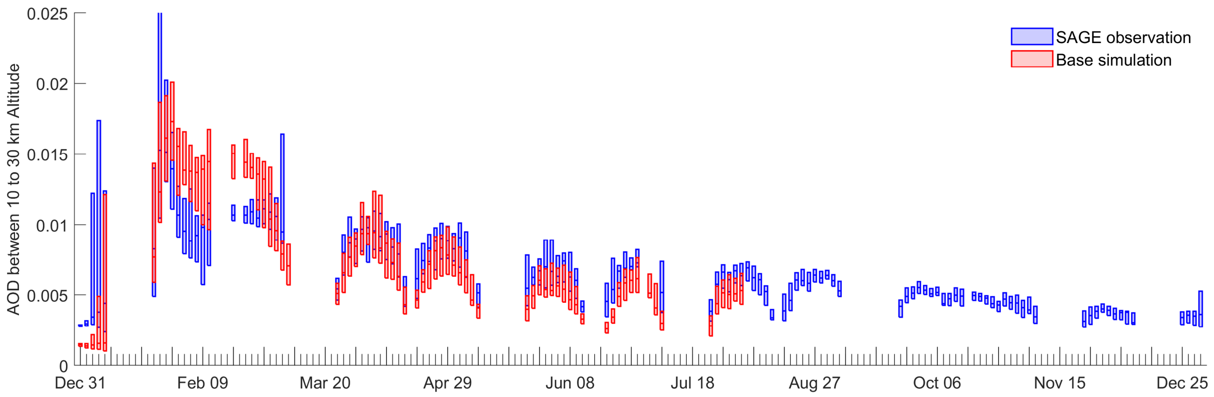

Fig. S5. Comparison of AOD at 1020 nm between SAGE III-ISS satellite observations and model simulations (Base Scenario) in the extratropical Southern hemisphere. Same as Fig. 1**A** except for an extended period.

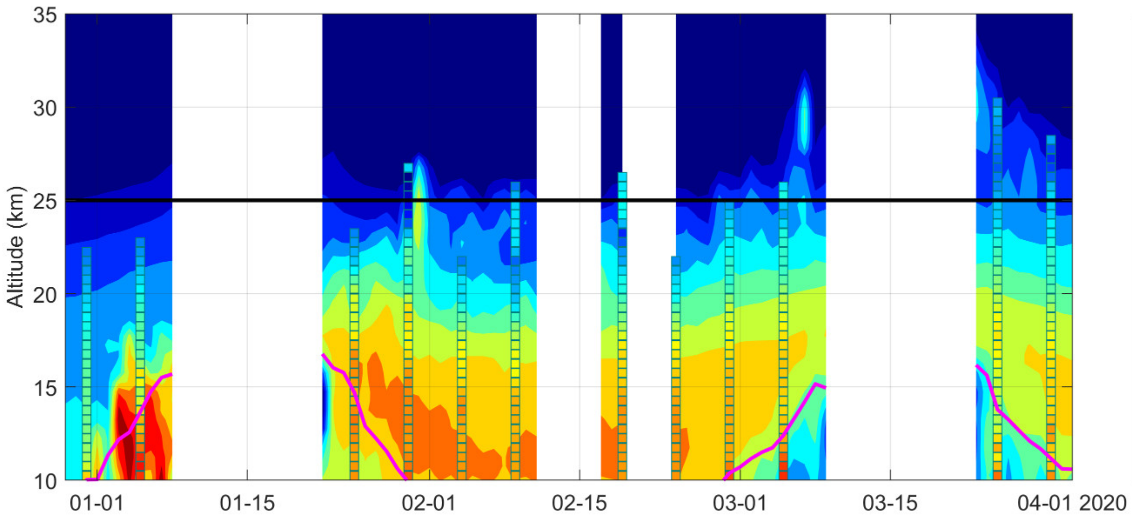

Fig. S6. Comparison of aerosol extinction coefficient at 1020 nm (Mm^{-1}) between SAGE III-ISS satellite observations (colored rectangles) and model simulations (color contour). SAGE observations were averaged every 5-days and data less than 5×10^{-2} Mm^{-1} at high altitude were not plotted. The simulation results were sampled at the same time and position of SAGE observations and daily averaged. Only profiles sampled within ESH were used here.

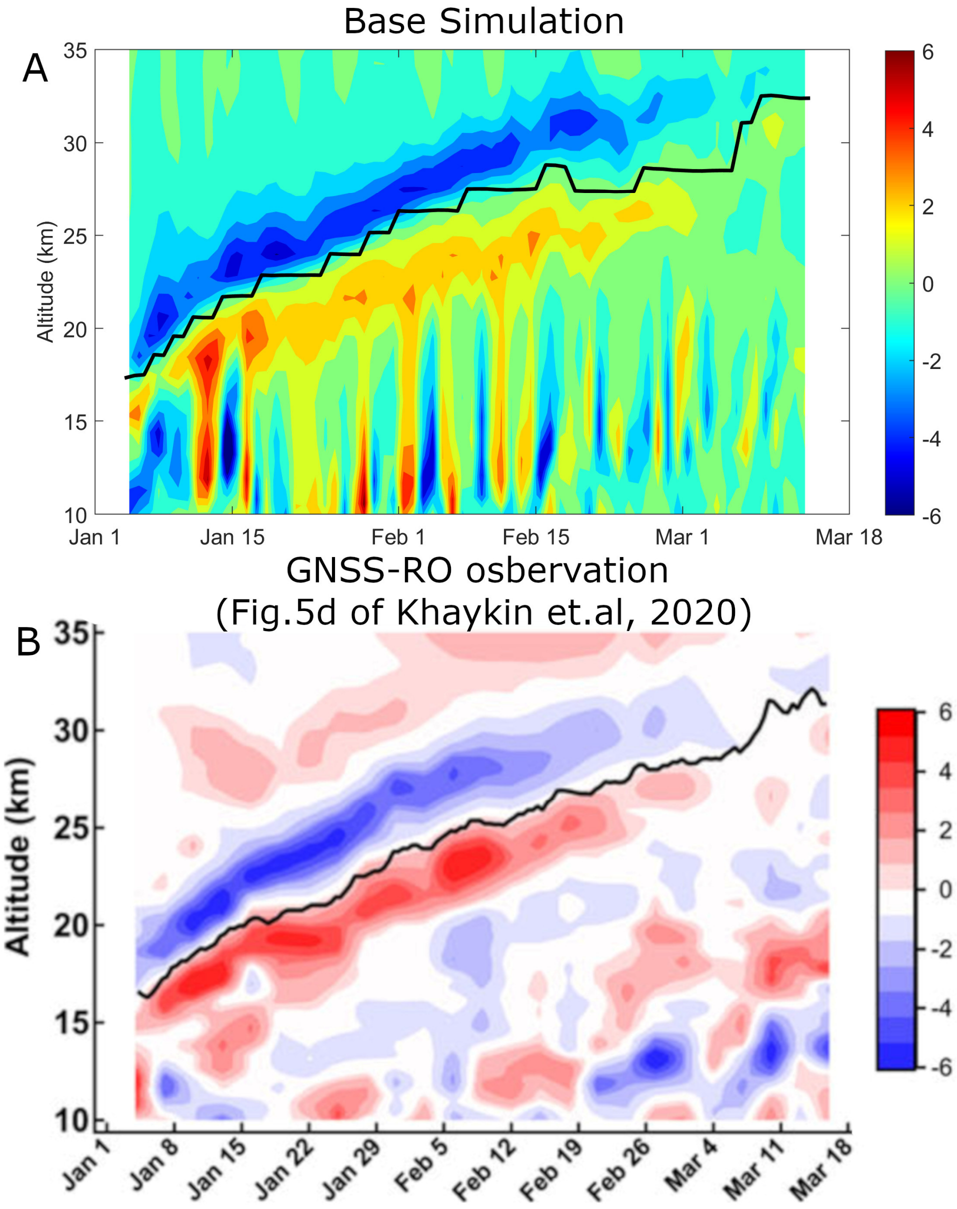

Fig. S7. Composite temperature perturbation (K) within the SCV. (**A**) Perturbation from Base simulation. The black line shows the SCV center simulated by Base. The perturbation was computed in a similar way as the Fig. 5d in Khaykin et al. (*11*). It is the departure of hourly temperature profiles collocated with the SCV center (Maximum LPV point) from a mean temperature profile within the corresponding spatiotemporal bin (3-day, 3° latitude, 40° longitude). The resultant hourly profiles were then lumped into daily profiles. (**B**) Perturbation from observations of Metop Global Navigation Satellite System (GNSS) radio occultation (RO) temperature profiles. The figure is a reprint of Fig. 5d in Khaykin et al. (*11*). The black line shows the center of vortex identified from ECMWF relative vorticity.

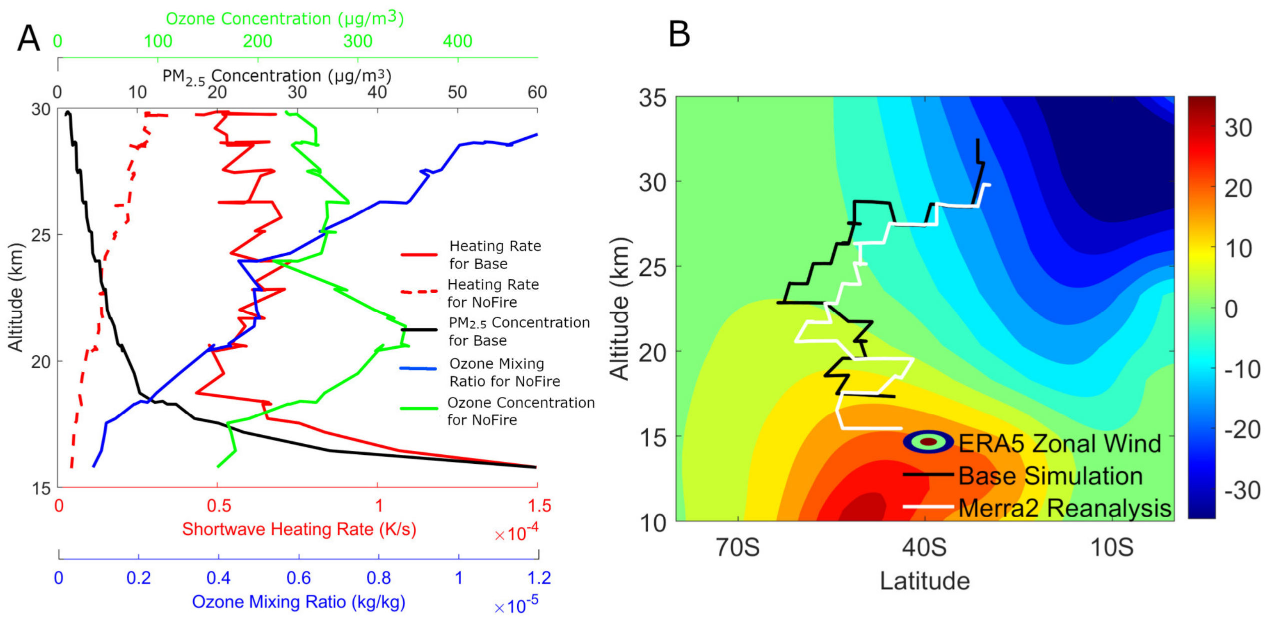

Fig. S8. Change of the SCV and its background with ascent. (**A**) Daily averaged properties sampled at the aerosol bubble center (in terms of maximum $PM_{2.5}$ concentration) of SCV. Shortwave heating rate (red Solid, K s^{-1}) and $PM_{2.5}$ concentration (black, µg m^{-3}) were retrieved from the Base simulation to represent the change of the SCV while the shortwave heating rate (red dashed), O_3 mixing ratio (blue, kg kg^{-1}) and concentration (green, µg m^{-3}) were retrieved at the same location but from NoFire to represent the change of the background. (**B**) Latitudinal and vertical distribution of zonal wind (color, m s^{-1}, positive value means westerlies) averaged longitudinally and from January to March of 2020. Black (white) line denotes the simulated (reanalysis) SCV trajectory in terms of maximum LPV points.

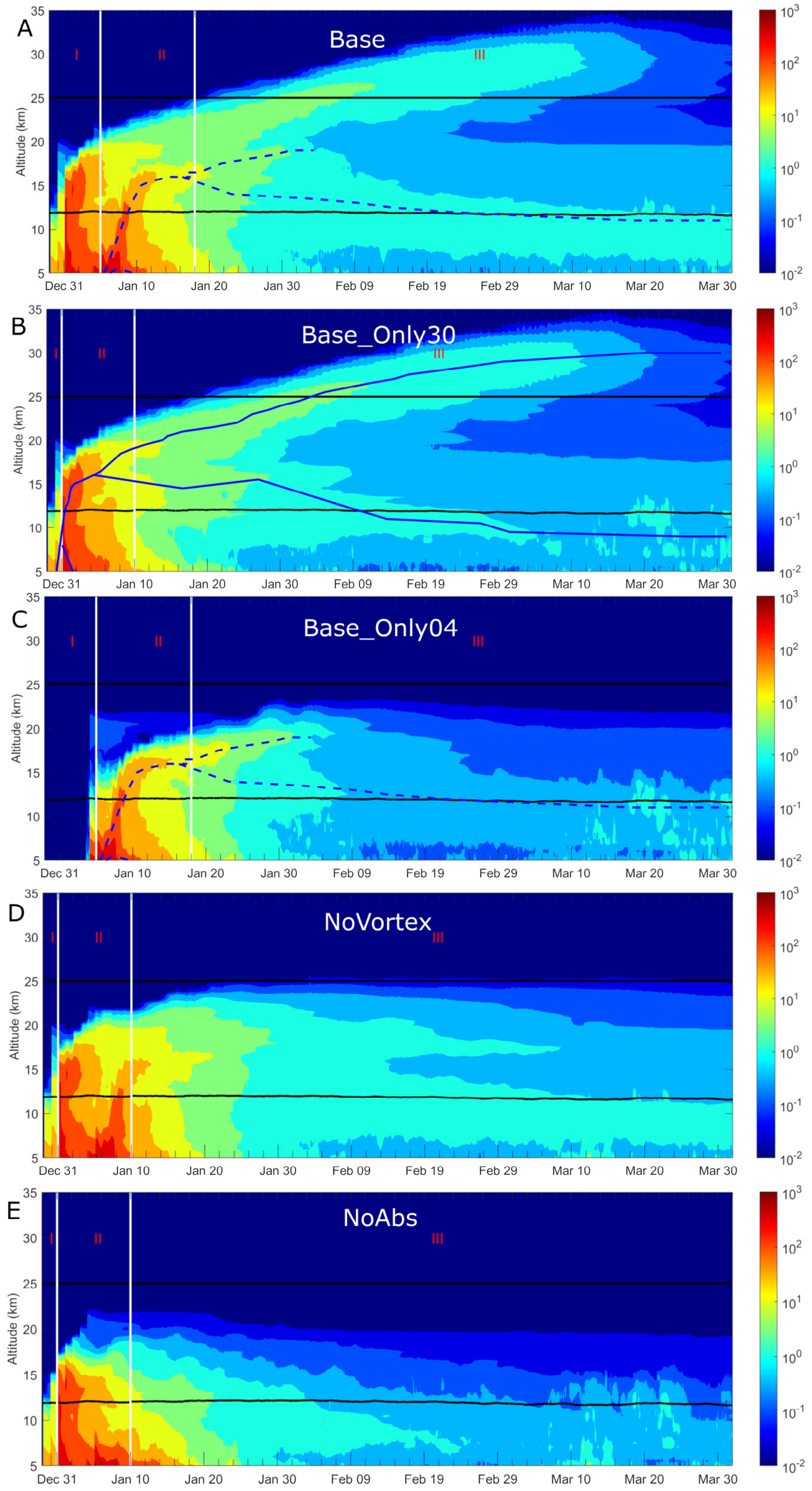

Fig. S9. Maximum $PM_{2.5}$ concentration (μg m^{-3}) in ESH, as Fig. 2B but from various simulations. Panel (**A**)-(**E**) represents results from Base, Base_Only30, Base_Only04, NoVortex, NoAbs simulations. The dashed blue lines shown in panel (**A**) and (**C**) were drawn by connecting local maxima of each vertical profiles from Base_Only04 which shows smoke from pyro-convection on 4 January 2020 and also ascends while forming the SCV P3 and therefore also experienced three stages. The solid blue lines in panel (**B**) were drawn by connecting local maxima of each vertical profiles from Base_Only30. Stage I and II are separated at the beginning

of 31 December 2019 (5 January 2020) for smoke released by pyro-convection on 30 December 2019 (4 January 2020) because the strongest pyro-cumulonimbi were both documented (http://pyrocb.ssec.wisc.edu/archives) and simulated by WRF-Chem on 30 December (4 January 2020). After that, pyro-convection is assumed to contribute little to the rise of BB plume and smoke stopped to rise as shown by (**E**). (Features of smoke rise near 20 km altitude before 6 January in (**E**) was artificially generated by data assimilation.) Stage II and III are separated on 10 January (18 January) when SCV P1 (SCV P3) matured (when a compact ellipsoidal aerosol bubble was formed) for smoke released by pyro-convection on 30 December 2019 (4 January 2020).

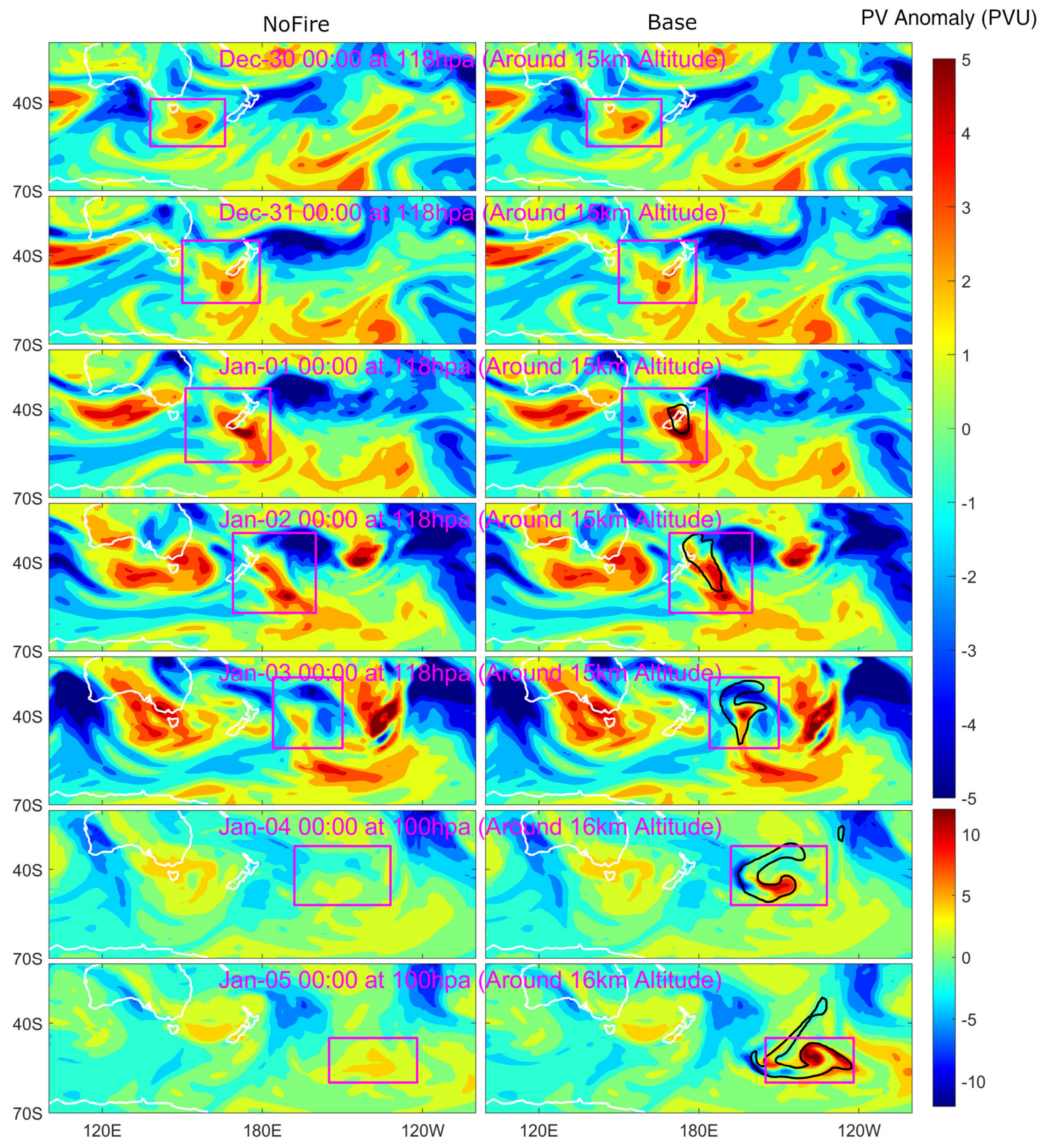

Fig. S10. Temporal evolution of Ertel PV (color, PVU) anomalies and $PM_{2.5}$ concentrations (black contours) of the SCV during formation. Magenta rectangles show the regions of interest. $PM_{2.5}$ concentrations shown in black contours indicate a $PM_{2.5}$ concentration of 10 μg m^{-3}. The left column shows the result from NoFire and the right is from Base. The PV anomaly is the difference between the Ertel PV and its zonal average. Note the change of altitude and color scale from 03 January to 04 January.

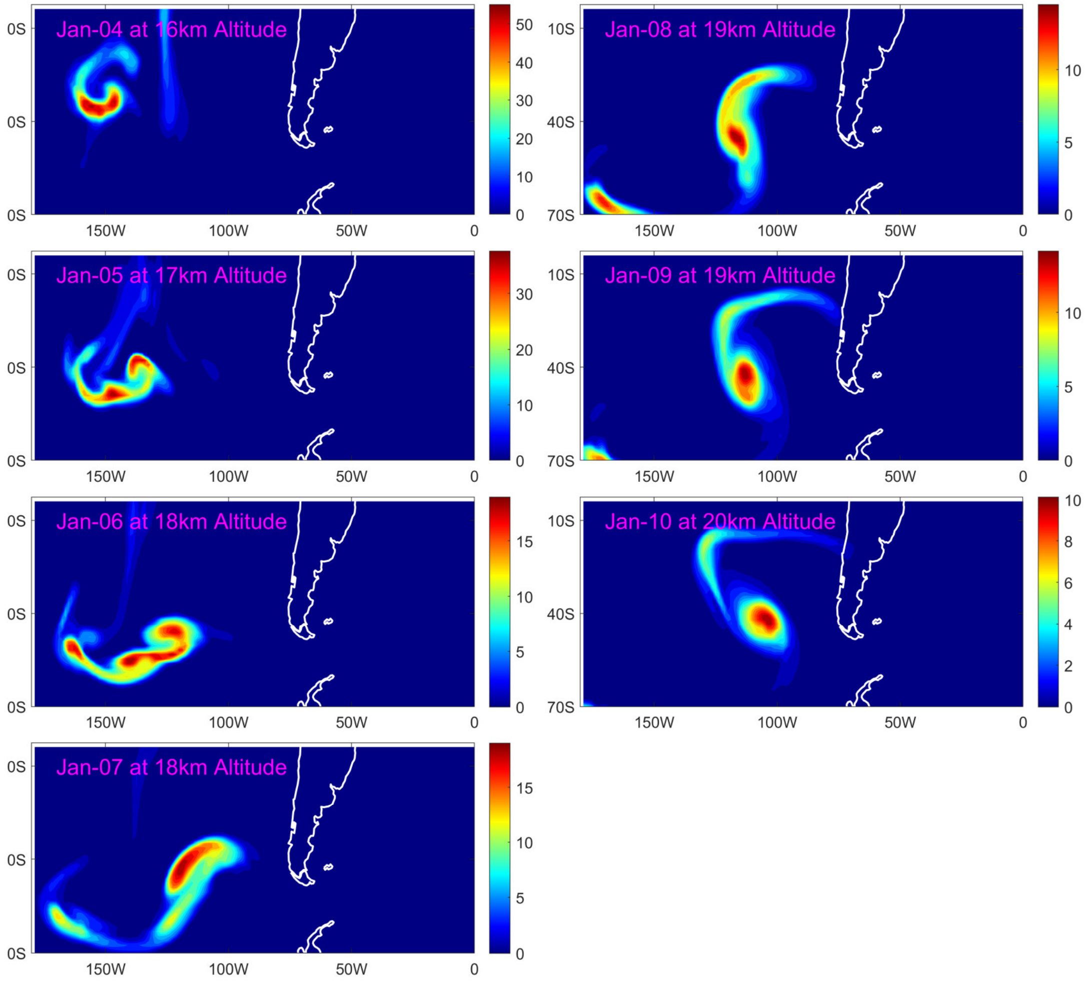

Fig. S11. Simulated $PM_{2.5}$ concentrations (μg m^{-3}) tracking the evolution of the SCV into maturation. The horizontal distribution is from Base_Only30. Note the change of color and altitude at different snapshots in time.

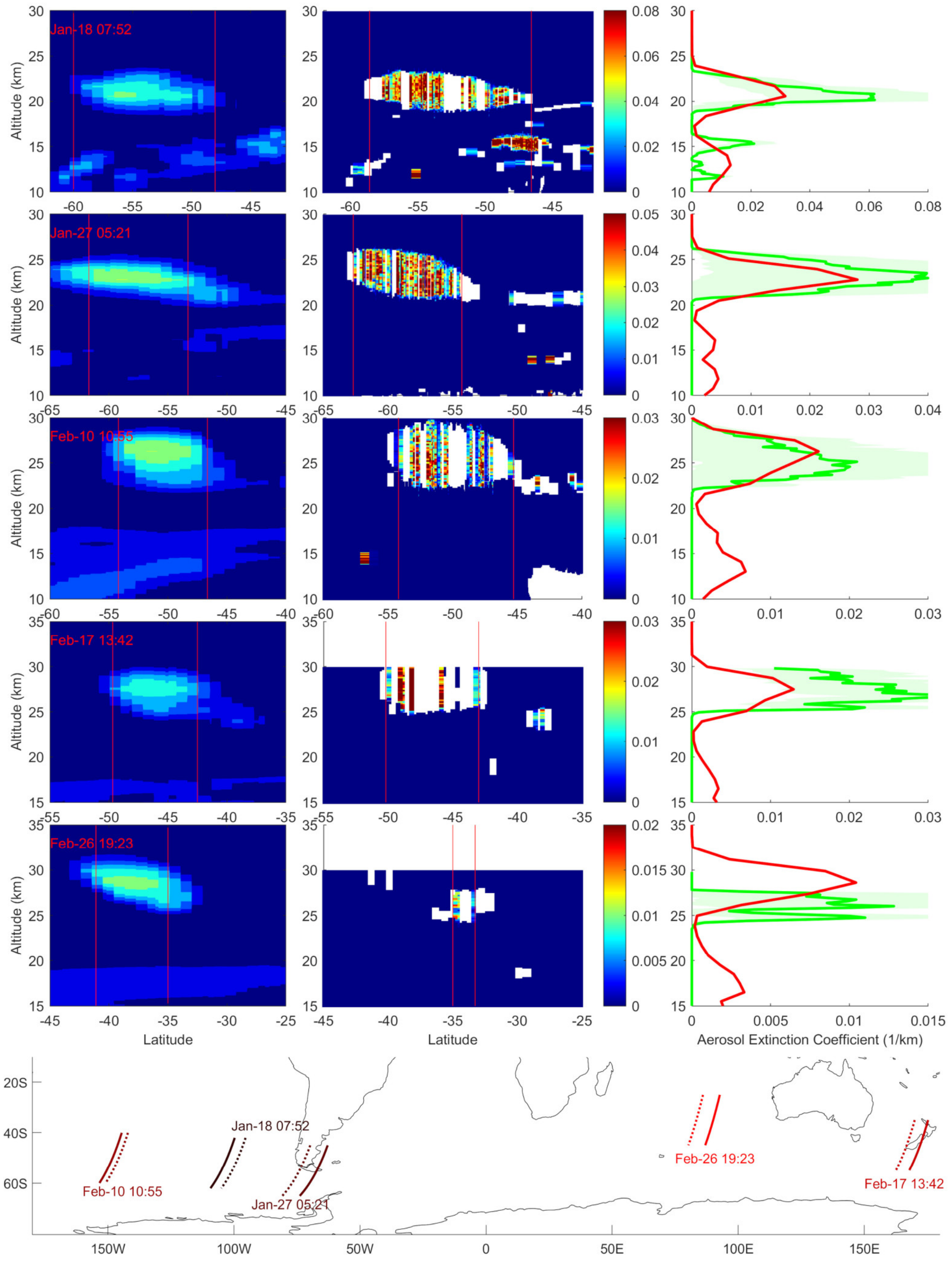

Fig. S12. Comparison of the aerosol extinction coefficient (km^{-1}) inside the SCV between CALIOP observations and Base simulation. The middle column shows the observed aerosol extinction coefficient curtain at 532 nm sampled by CALIOP crossing the SCV while the corresponding Base simulations at 550 nm are in the left column. Simulations were retrieved by displacing the CALIOP track a bit to cross the concentration center of the simulated SCV. Extinction profiles averaged horizontally between the two red vertical lines were plotted in the right column with red as the simulation and green as the observation (green shade represents the maximum uncertainty). Observed pixels classified as "Clean Air" were assumed to have zero aerosol extinction while other non-aerosol pixels were plotted blank and excluded from calculating the average. The bottom panel shows the tracks of the curtains from the observation (solid lines) and simulation (dashed lines).

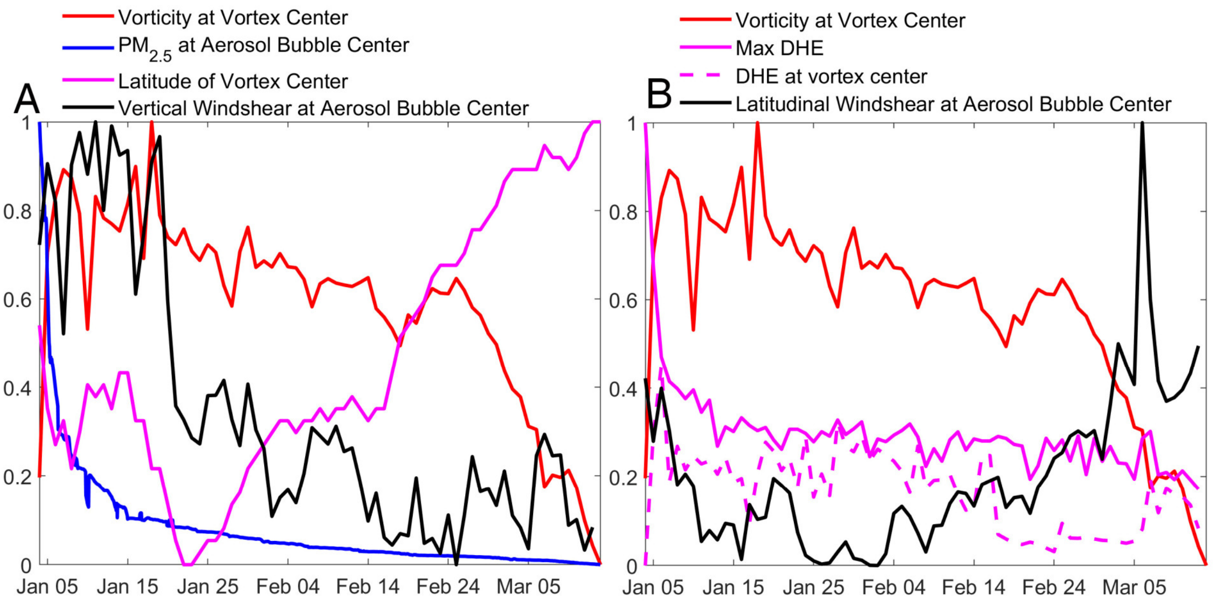

Fig. S13. Temporal evolution of daily averaged properties sampled along the simulated SCV. (**A**) Temporal evolution of vorticity at the vortex center, $PM_{2.5}$ concentration at the aerosol bubble center, the latitude of the vortex center and the vertical windshear at the aerosol bubble center. The vortex center refers to the point where the maximum relative vorticity is reached around the SCV and similarly the aerosol bubble center is where maximum $PM_{2.5}$ concentration is reached. All values were scaled to [0,1] to show the relative temporal change. (**B**) Similar to (**A**) but for vorticity at the vortex center, Max DHE (the DHE at the point where it reaches its maximum inside the aerosol bubble), DHE at the vortex center and the latitudinal windshear at the aerosol bubble center. Max DHE and DHE at the vortex center were scaled together. Both vertical and latitudinal windshear only consider the zonal wind. As can be seen from panel (**B**) the sudden decrease of relative vorticity from mid-February was accompanied by the sudden deviation of DHE at vortex center from maximum DHE and the increase of latitudinal wind shear. Meanwhile $PM_{2.5}$ concentration, vertical wind shear and maximum DHE only changed slowly.

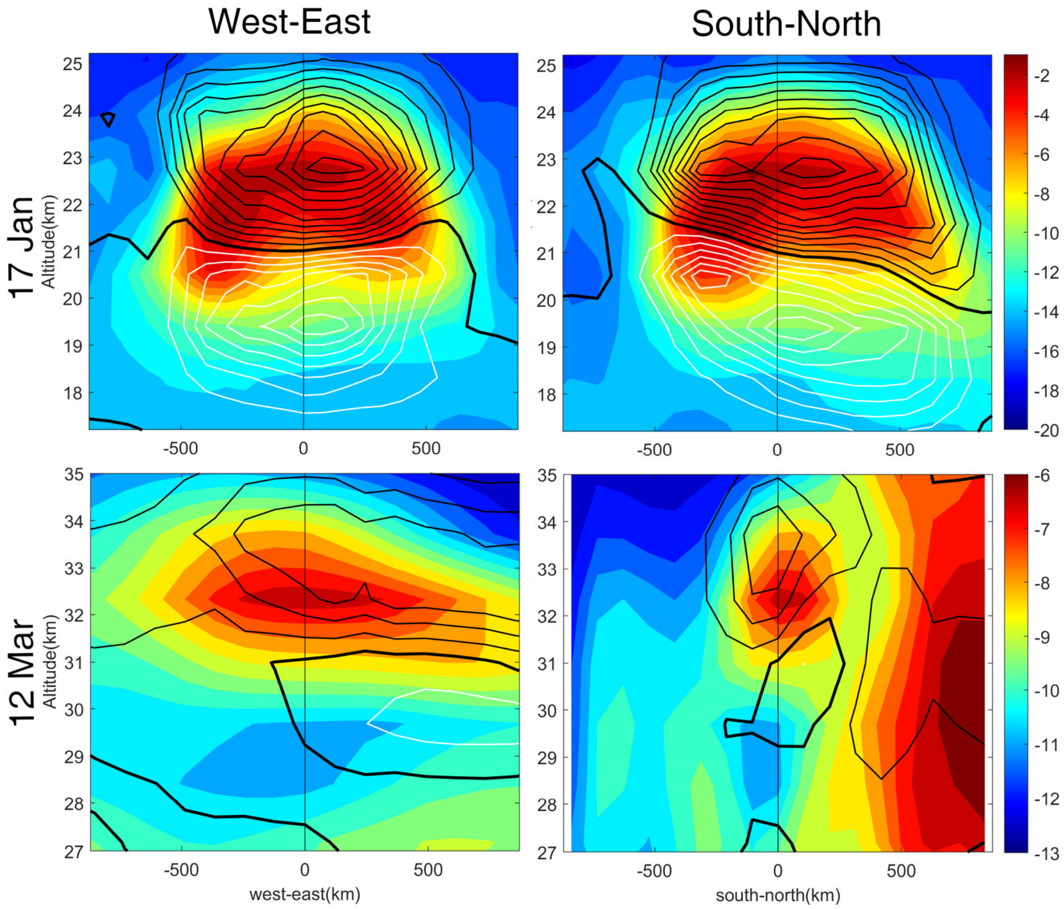

Fig. S14. Vertical cross sections of simulated LPV (color, PVU) and DHE (black contour) fields taken zonally (left) or meridionally (right) cross the center of the SCV. The upper and bottom rows correspond to the maturation and dissipation periods respectively. Contours denote the DHE with interval of $4^{-11}s^{-2}$ and black (white) lines as positive (negative). By comparing the top (maturation) and bottom (dissipation) row, the shape of SCV turned into a narrow feature before dissipation.

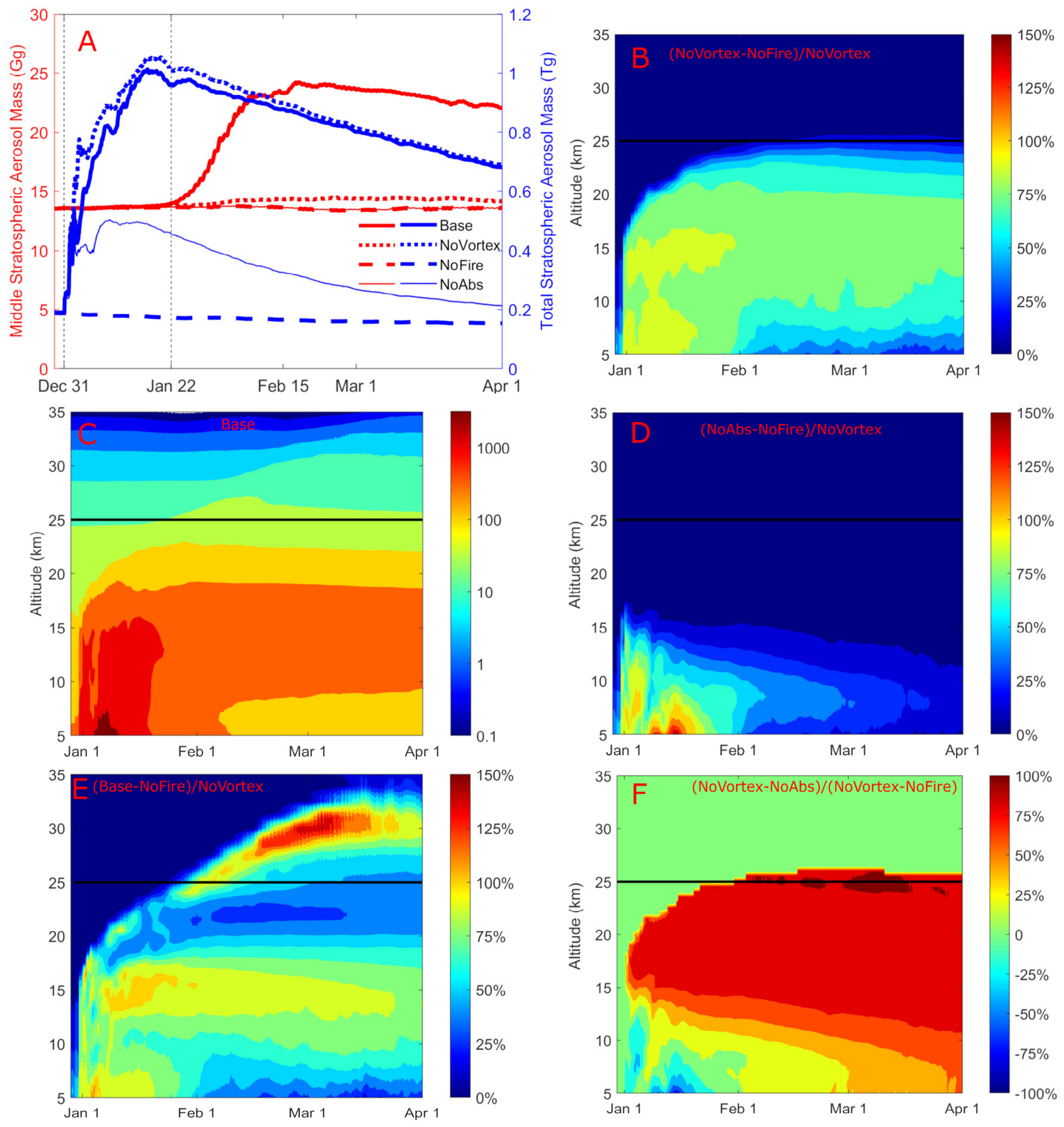

Fig. S15. Impact of SCV and BB on stratospheric aerosol concentration. (**A**) Temporal evolution of total $PM_{2.5}$ mass like Fig. 4B except including the results of NoAbs. (**B**) Temporal and vertical distribution of the difference between $PM_{2.5}$ concentrations (averaged over ESH) from NoVortex and NoFire normalized by NoVortex. (**C**) $PM_{2.5}$ concentration (ng m^{-3}) averaged over ESH from Base simulation. (**D**) The same as (**B**) expect difference are between NoAbs and NoFire. (**E**) The same as (**B**) expect difference are between Base and NoFire. (**F**) The contribution of BC heating to $PM_{2.5}$ in the ESH during the 2019/20 Australian wildfire events estimated from NoVortex and NoAbs.

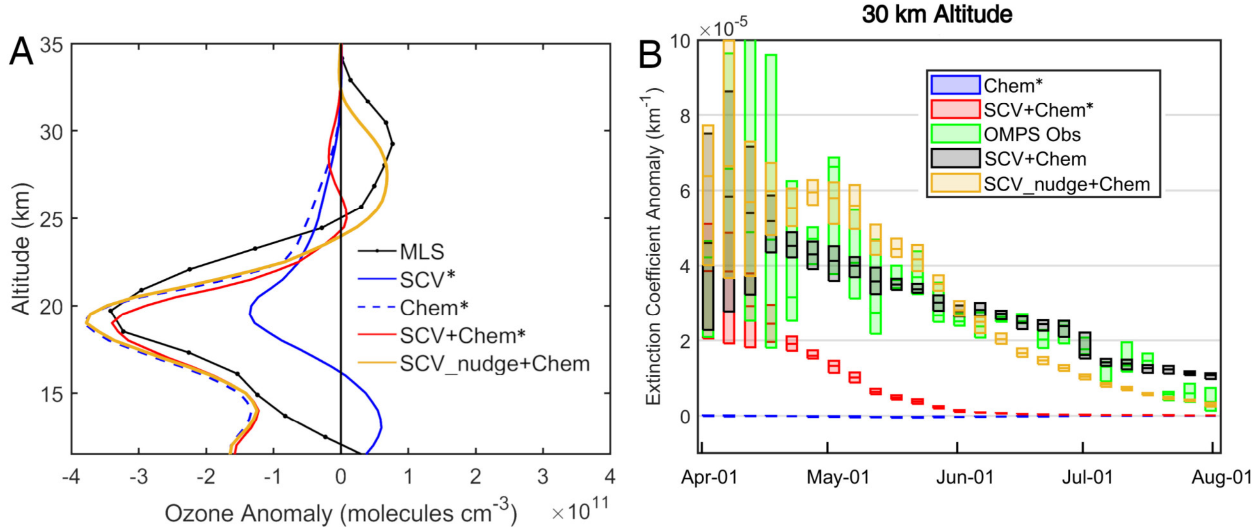

Fig. S16. Impact of SCV and BB on stratospheric ozone and aerosol. (**A**) The same to Fig. 6A except from simulations with aerosol not constrained by SAGE satellite observations, marked with asterisk (*). The "Chem*" represents the anomaly of the simulation with BB aerosol and additional heterogeneous reactions but without SCV formed. The new SCV_nudge+Chem simulation nudged aerosols amount exclusively around the SCV, and exclusively in the genesis period of SCV, to explain the differences between SCV+Chem* and observation and to exclude the influence of SCV-unrelated aerosols. In SCV_nudge+Chem, we nudged the concentration of aerosols inside the SCV according to CALIOP aerosol extinction observations right after its first formation on 17 January for model grids in SCV, i.e., those with BC mixing ratio over $2x10^{-10}$ kg kg^{-1}. After that on 13 March, the aerosols were redistributed around the SCV making them concentrated at the position of SCV according to reanalysis, leading to the reformation of SCV on 13 March after its shear-induced breaking near 10 March (*11*). As shown here, the new SCV_nudge+Chem results in similar O_3 results as simulations with continuous aerosol constraining (red line in Fig. 6A) anomaly based on SAGE observation, indicating again the O_3 anomaly in middle stratosphere is mainly driven by SCV induced aerosol heterogeneous reactions. (**B**) Aerosol extinction coefficient anomaly from OMPS satellite observations and model simulations. Anomalies from OMPS observations are calculated as the difference of 675 nm aerosol extinction coefficient between 2020 and the average of 2012-2019. Simulated anomalies are calculated as the difference between a specific case and the no wildfire control case (NoFire). The upper, middle, and lower lines in the Whisker plots represent the 25th, 50th, and 75th percentiles of the sampled 5-d data at the 30 km altitude between 30°S and 50°S.

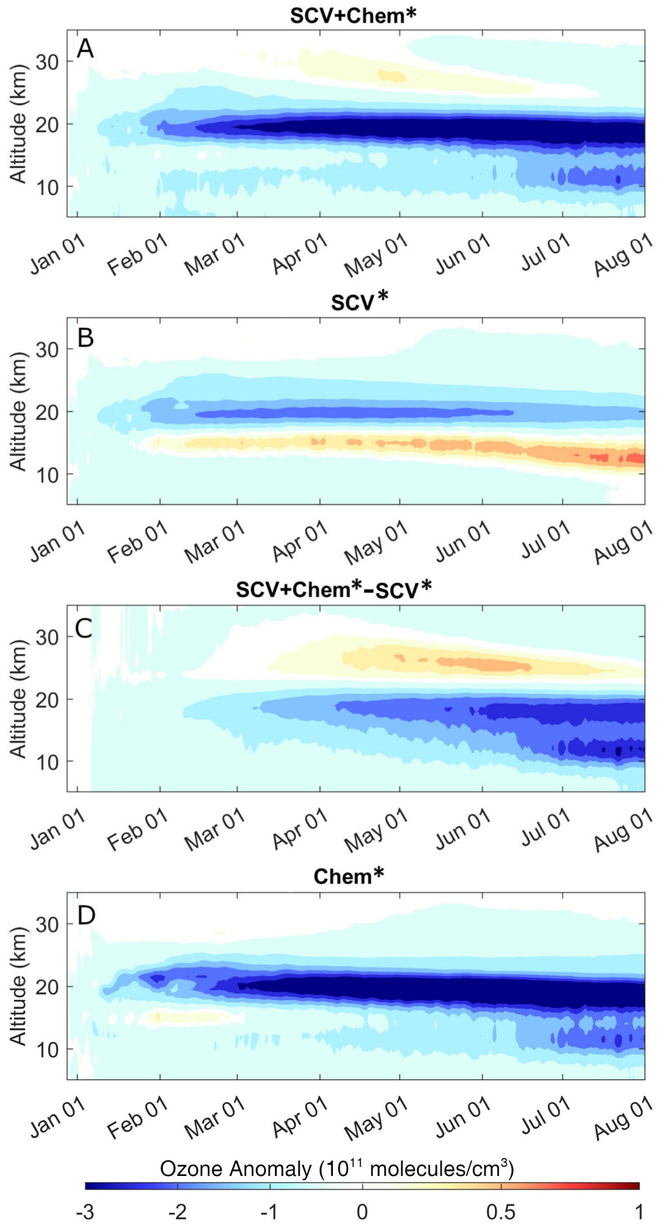

Fig. S17. Simulated temporal and vertical distribution of ozone anomaly caused by BB aerosols. (**A**) The contribution from both non-chemistry processes and newly introduced heterogeneous reactions. (**B**) Impact solely from non-chemical processes. (**C**) The same as Fig. 6D denoting the contribution from heterogeneous reactions only. (**D**) Impact from both non-chemical processes and newly introduced heterogeneous reactions but without the formation of an SCV. Like Fig. 6D, the O_3 anomaly was calculated as O_3 concentration averaged over 30-50°S from simulations with Australian fire events minus the NoFire scenario. Unlike Fig. 6D, simulations are from model runs without SAGE constrained aerosol (the same as Fig.S16A).

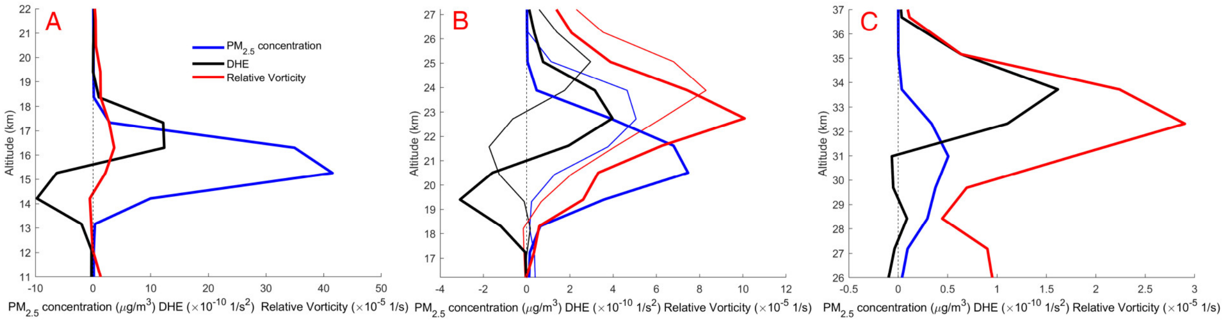

Fig. S18. Profiles of vorticity, DHE and $PM_{2.5}$ concentration cross the center of the SCV at different stages of plume rise in simulations. (**A**) 23:00 UTC of 03 January in the formation period. (**B**) Thick lines are from the simulation at 19:00 UTC of 17 January and thin lines are from 17:00 UTC of 25 January. Both periods represent the maturation stage. (**C**) 19:00 UTC of 12 March in the dissipation period. Unlike Fig. 3, the scale for x-axis changes from (**A**) to (**C**).

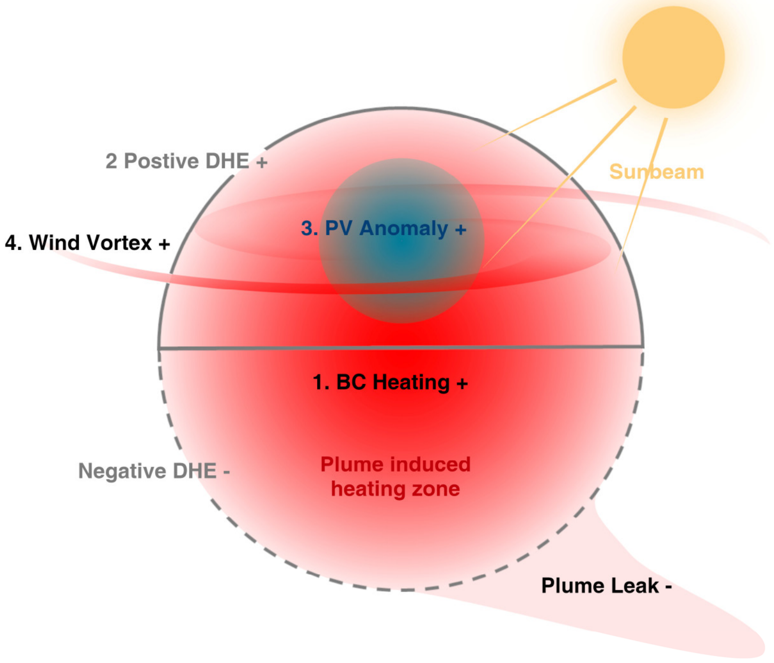

Fig. S19. Schematic illustration explaining the main processes involved during the maturation period of the SCV.

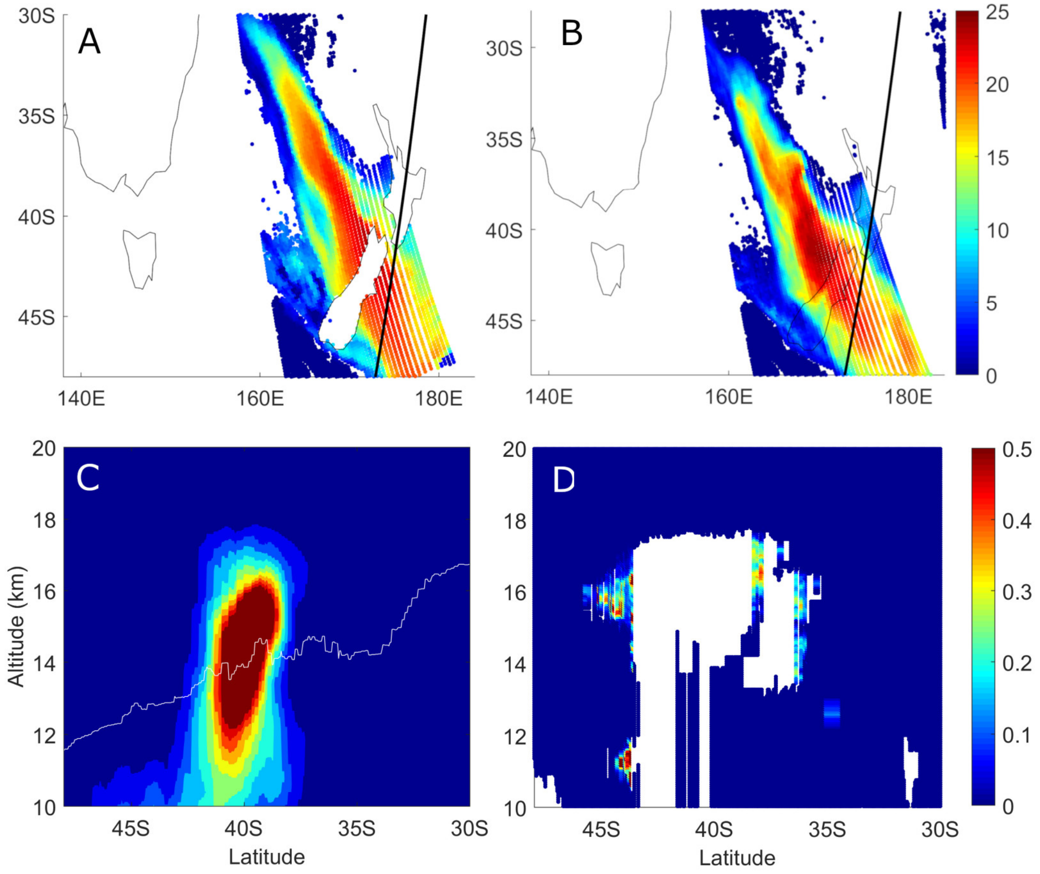

Fig. S20. Comparison of AI and aerosol extinction coefficient between WRF-Chem/DART reanalysis and observations. (**A**) Reanalysis AI distribution at 02:00 UTC of 1 January 2020 calculated from the aerosol concentration field of the WRF-Chem/DART reanalysis; the thick black line represents the position of the CALIOP track for panel (**C**) and (**D**). (**B**) AI observations near 02:00 UTC of 1 January 2020 from TROPOMI. (**C**) Reanalysis aerosol extinction coefficient (km^{-1}) at 532 nm of 1 January 2020 14:00 UTC; the white line is the tropopause simulated by WRF-Chem. (**D**) Observed aerosol extinction coefficient (km^{-1}) curtain at 532 nm from CALIOP near 14:00 UTC of 1 January 2020. Pixels classified as "Clean Air" were assumed to have zero aerosol extinction while other non-aerosol pixels were plotted blank.

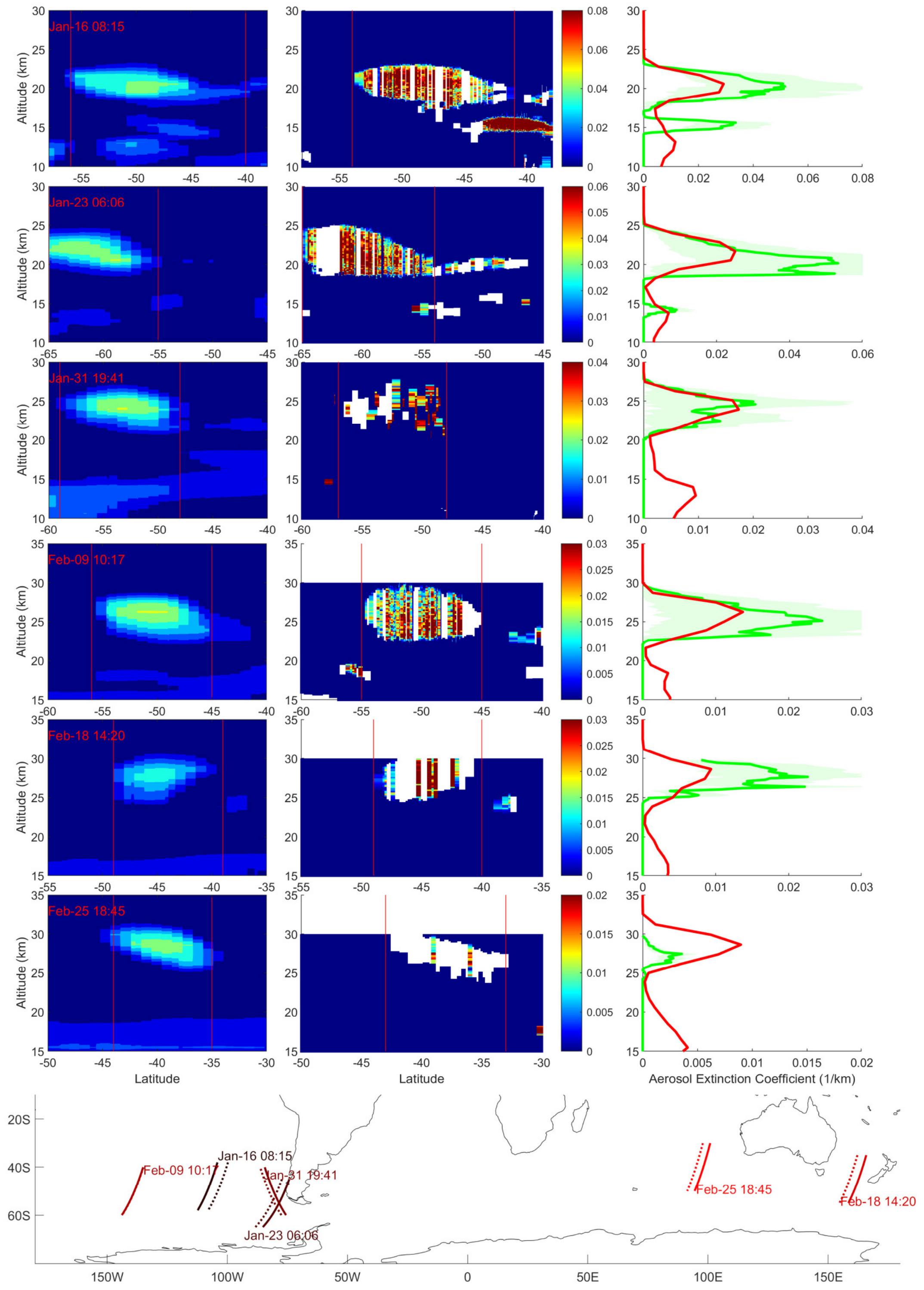

Fig. S21. Comparison of the aerosol extinction coefficient (km[-1]) inside the SCV between CALIOP observations and Base simulation. The same as Fig. S12 except for different time periods.

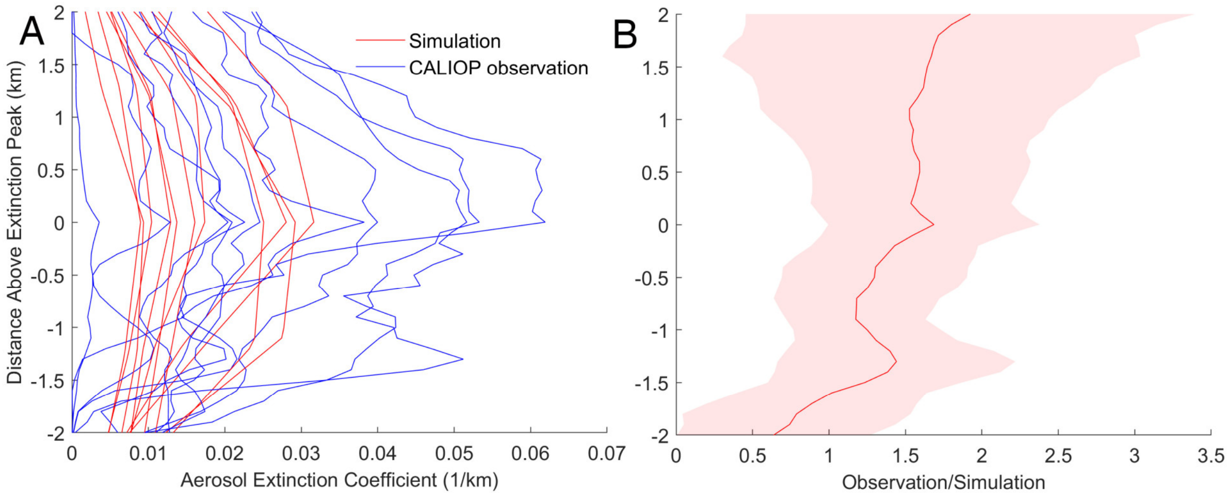

Fig. S22. Difference of aerosol extinction profiles between simulated and observed SCV. (**A**) 11 extinction profiles (averaged along the sampling track) collected from the right column of Fig. S12 and Fig. S21. Vertical profiles from either simulation or CALIOP observation were aligned to the same height coordinate according to where they reach the maximum extinction. (**B**) Ratio of observed profile to corresponding simulation averaged for the 11 comparisons. The ratio (defined as Observation/Simulation) was calculated for each comparison and then averaged to be the red line. The red shading denotes ±1 std across different comparisons.

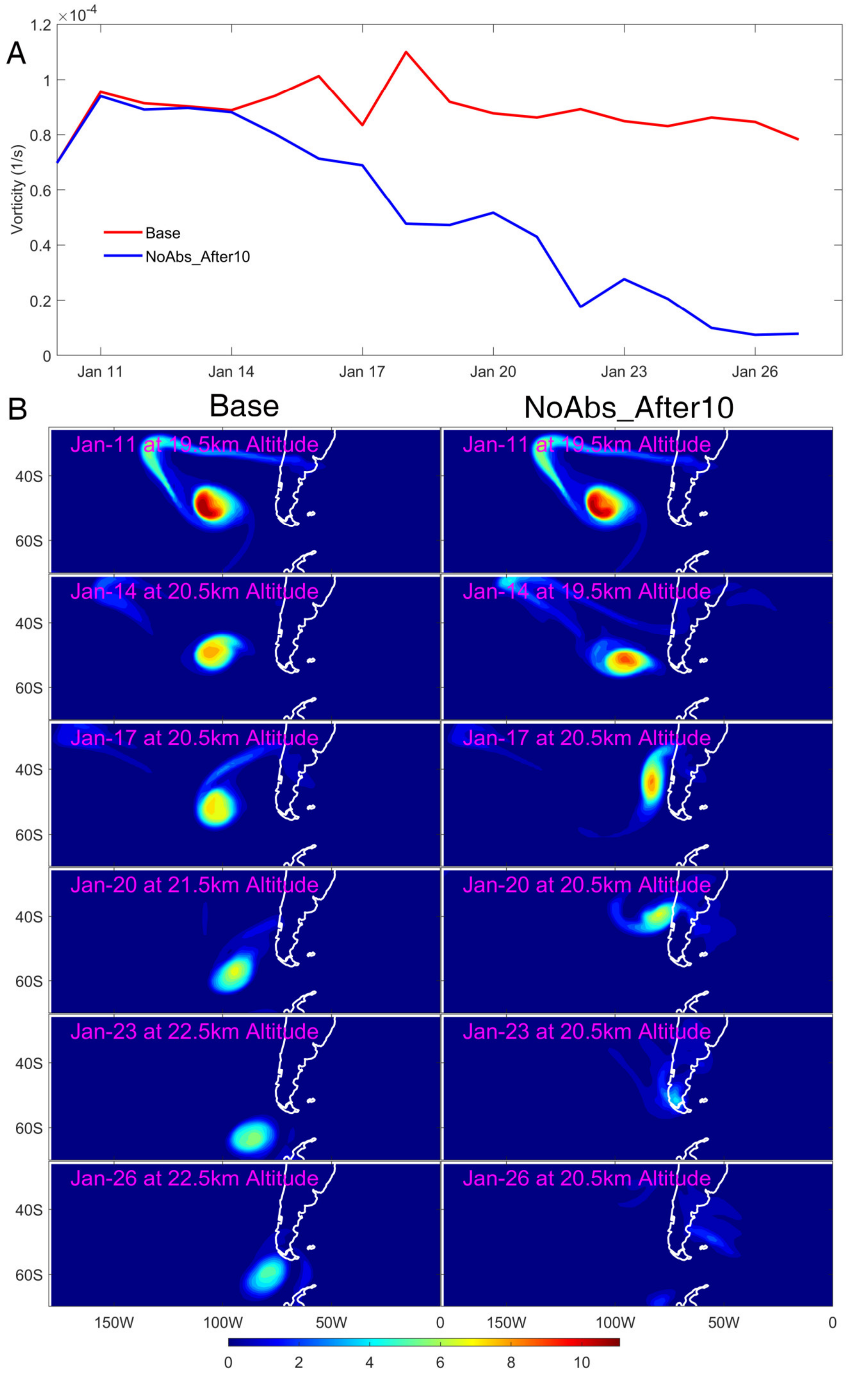

Fig. S23. Comparison of the simulated SCV evolution between Base and NoAbs_After10. **(A)** Evolution of the maximum relative vorticity within the SCV from the Base simulation and the simulation without BC absorption after 10 January (NoAbs_After10). **(B)** The horizontal distribution of $PM_{2.5}$ concentrations (μg m^{-3}) from Base and NoAbs_After10. Note the change of altitude at different time snapshots for different simulations.

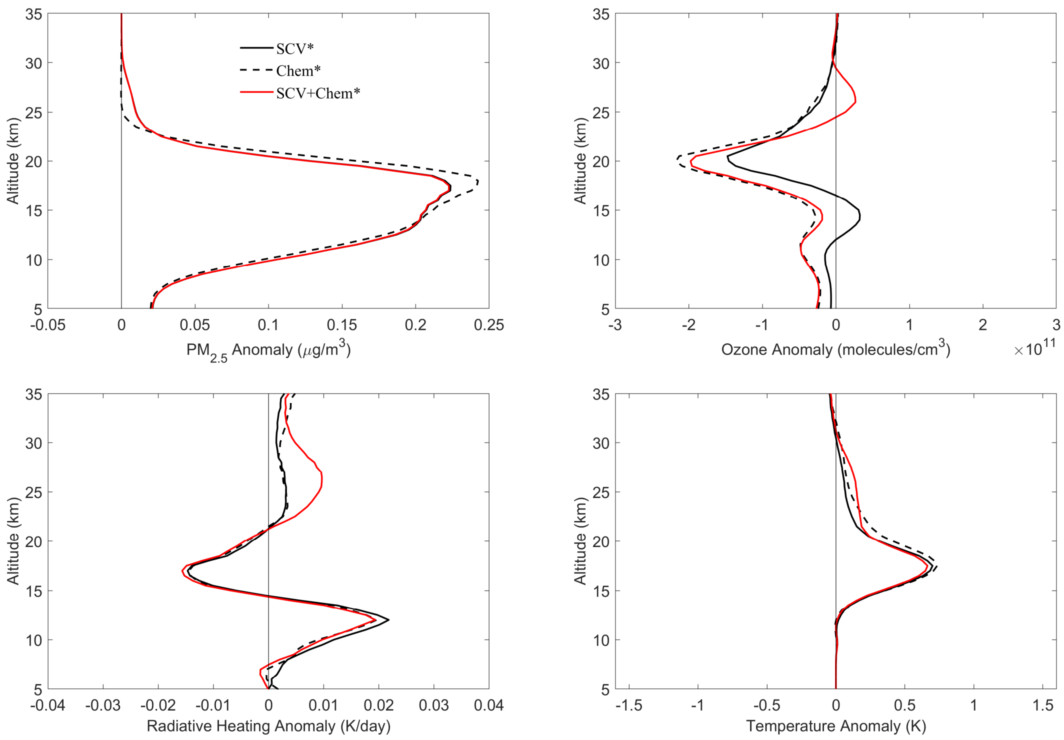

Fig. S24. Impact of SCV and BB on stratospheric ozone and thermal structures. The anomaly of $PM_{2.5}$ (top left), O_3 (top right), radiative heating (bottom left, longwave plus shortwave) and temperature (bottom right) is the averaged difference between a simulation with BB aerosol and NoFire. Simulations are from model run without SAGE constrained aerosol like Fig. S16A. The average was taken between 20°S and 60°S in May 2020. The latitude range is 20-60°S (instead of 30-50°S in Fig. 6) in order to be comparable to reanalysis temperature anomaly from previous research (Yu et al. (*18*), which reported a ~0.5 K temperature anomaly for the same period and latitude range at 17 km altitude).

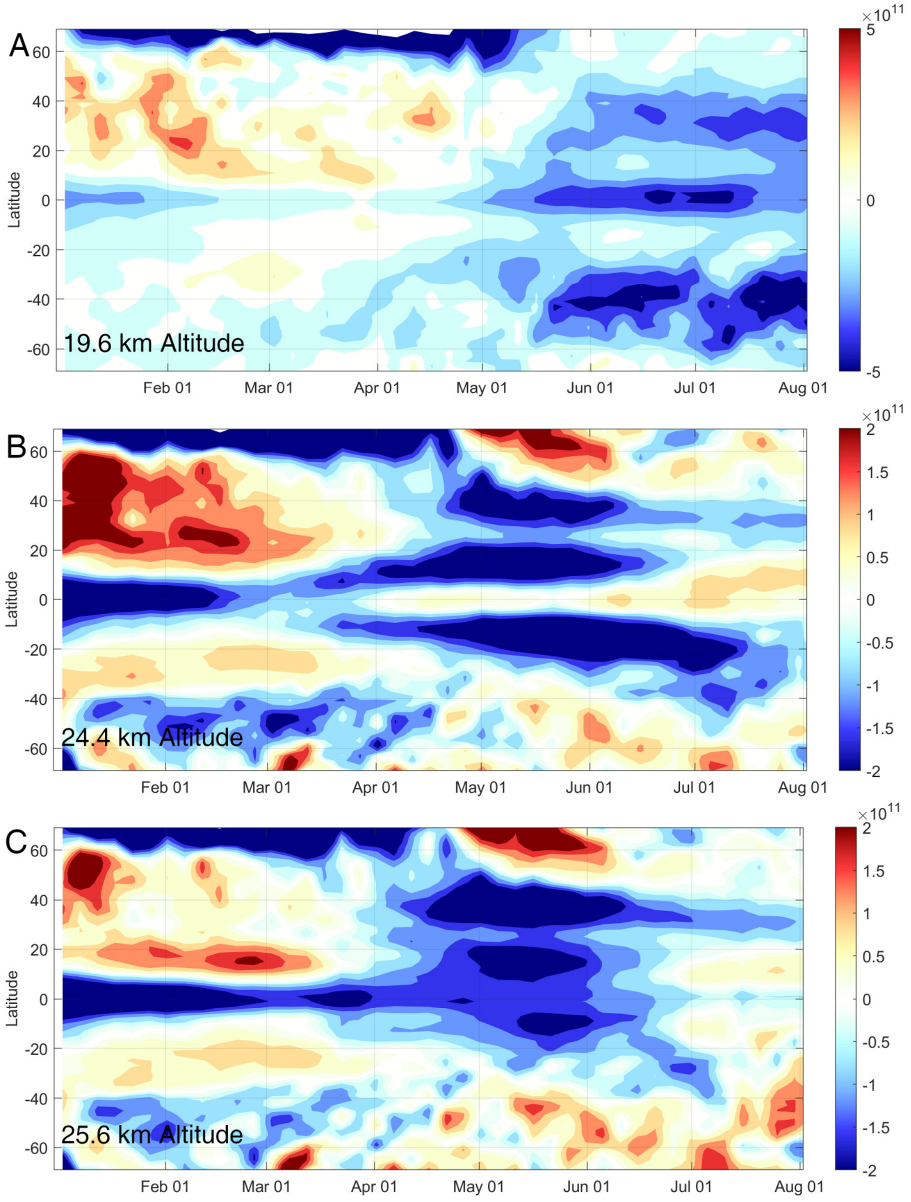

Fig. S25. MLS satellite observations for the deseasonalized ozone anomaly of year 2020 as a function of time and latitude. (A) Horizontal cross section at 19.6 km altitude where is the lower stratosphere. **(B)** At 24.4 km altitude. **(C)** At 25.6 km altitude where is the middle stratosphere. Deseasonalized O_3 anomaly (molecules cm^{-3}) was calculated by averaging observations in each year by 5-day and 2°-latitude bins and then subtracting the mean of year 2005-2019 from 2020 for each bin. Note the color scale of **(A)** is different from that of **(B)** and **(C)**.

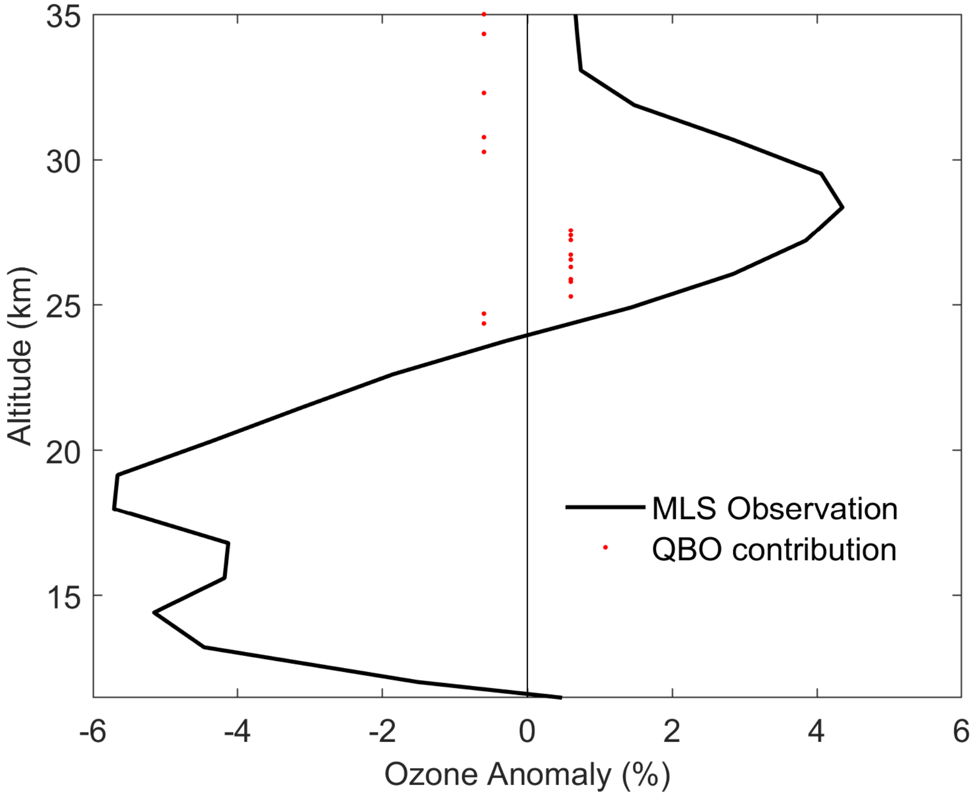

Fig. S26. Observed ozone anomaly and effects of QBO transport in the middle stratosphere. The MLS vertical profile is the percent anomaly of 45-60°S and July average while the QBO contribution is from the Figure 3d of Diallo et al. (*109*). On average, the contribution of QBO was around 0.1% above 24 km, far less than the total anomaly up to 5%.

Table S1.

Summary of the configurations for WRF-Chem/DART.

DART Parameters	Value	WRF-Chem Parameters	Value
Filter type	Ensemble adjustment Kalman filter	Horizontal grid	15 km, covering southeastern Australia and New Zealand
Covariance inflation	False	Vertical grid	43 layers, 29 km model top
Localization type	Gaspari–Cohn	Chemistry scheme	MOZCART
Horizontal localization half-width	318.6 km	Meteorological fields	Overwritten by perturbed FNL reanalysis every 6 hours
Vertical localization half-width	0.075 scale height (approximately 0.65 km at 298 K)	BB emission	GFED4s scaled by 7 and perturbed by 50%. Additional perturbation of BC emission ratio was also introduced.
Ensemble members	20	Plume rise	1-D smoke plume rise model with entrainment intensity perturbed by 60%
Assimilation interval	Every 6h at 02:00, 08:00, 14:00 and 20:00 UTC	BC absorption	Mass of BC was scaled by 1.64 only when calculating optical properties
Observations assimilated	TROPOMI aerosol index, MODIS AOD		
Model variables updated	BC and OC		

Table S2.

Summary of CAM-Chem simulations.

	Reanalysis to nudge	Nudge style	ANYSO aerosol from WRF-Chem/DART reanalysis	BC absorption
Base	Original Merra2	Two loosely-nudge windows following the simulated and observed SCVs (2nd paragraph of Sect. 1.4b)	From 29 December 2019 to 6 January 2020	Considered
NoVortex	Merra2 modified to diminish SCVs	Strictly-nudge windows following the simulated SCVs (3rd paragraph of Sect. 1.4b)	The same to Base	Considered
NoFire	The same to Base	The same to Base	No ANYSO aerosol	Considered
Base_Only30	The same to Base	The same to Base	From 29 December 2019 to 3 January 2020	Considered
Base_Only04	The same to Base	The same to Base	From 4 January 2020 to 6 January 2020	Considered
NoAbs	The same to Base	The same to Base	The same to Base	Not considered
NoAbs_After10	The same to Base	The same to Base	The same to Base	Not considered after 10 January 2020

Table S3.

Summary of candidate vortices in the stratosphere after the year 2009. The cases were identified by searching for isolated vortex-like Ertel PV anomalies in the extratropical stratosphere in the MERRA2 reanalysis data.

Duration	Position example	ERA5 Ozone Anomaly?	CALIOP Aerosol Anomaly?	Reference
2009/02/11 to 2009/03/05	(98°E, 20°S, 52 hPa) 2009/02/25	Yes	**Yes**	Trepte et al. (*132*)
2015/07/09 to 2015/07/16	(18°E, 76°N, 200 hPa) 2015/07/11	Yes	**Yes**	
2015/09/04 to 2015/09/10	(5°E, 64°N, 100 hPa) 2015/09/07	Yes	No	
2016/05/14 to 2016/05/26	(112°E, 76°N, 85 hPa) 2016/05/17	Yes	No	
2016/09/01 to 2016/09/12	(122°W, 77°N, 100 hPa) 2016-09-05	Yes	No	
2016/10/03 to 2016/10/07	(134°W, 77°N, 72 hPa) 2016/10/03	Yes	No	
2017/08/16 to 2017/10/18	(28°E, 61°N, 72 hPa) 2017/09/03	Yes	**Yes**	Lestrelin et al. (*22*)*
2017/09/21 to 2017/10/01	(168°E, 57°N, 118 hPa) 2017/09/24	Yes	No	
2018/05/24 to 2018/05/28	(48°E, 51°N, 100 hPa) 2018/05/27	Yes	No	
2018/07/20 to 2018/08/13	(102°E, 40°S, 43 hPa) 2018/07/29	Yes	No	
2019/03/10 to 2019/03/19	(27°W, 42°N, 61 hPa) 2019/03/14	Yes	No	
2019/03/05 to 2019/03/26	(37°E, 39°N, 61 hPa) 2019/03/14	Yes	No	
2020/01/04 to 2020/03/08	(78°W, 53°S, 31 hPa) 2020/01/31	Yes	**Yes**	Kablick III et al. (*12*) and Khaykin et al. (*11*)
2020/01/08 to 2020/03/03	(70°W, 72°S, 36 hPa) 2020/02/02	Yes	**Yes**	
2020/01/17 to 2020/02/02	(35°E, 47°S, 85 hPa) 2020/01/23	Yes	**Yes**	

* This case consisted of multiple SCVs which however all originated from one SCV ("Vortex O" as named by Lestrelin et al. (*22*)).

REFERENCES AND NOTES

1. G. A. Ban-Weiss, L. Cao, G. Bala, K. Caldeira, Dependence of climate forcing and response on the altitude of black carbon aerosols. *Climate Dynam.* **38**, 897–911 (2012).

2. P. Bernath, C. Boone, J. Crouse, Wildfire smoke destroys stratospheric ozone. *Science* **375**, 1292–1295 (2022).

3. Z. Dai, D. Weisenstein, D. Keith, Tailoring meridional and seasonal radiative forcing by sulfate aerosol solar geoengineering. *Geophys. Res. Lett.* **45**, 1030–1039 (2018).

4. S. Tilmes, J. Richter, B. Kravitz, D. MacMartin, A. Glanville, D. Visioni, D. Kinnison, R. Müller, Sensitivity of total column ozone to stratospheric sulfur injection strategies. *Geophys. Res. Lett.* **48**, e2021GL094058 (2021).

5. D. A. Peterson, M. D. Fromm, R. H. McRae, J. R. Campbell, E. J. Hyer, G. Taha, C. P. Camacho, G. P. Kablick, C. C. Schmidt, M. T. DeLand, Australia's Black Summer pyrocumulonimbus super outbreak reveals potential for increasingly extreme stratospheric smoke events. *npj Clim. Atmos. Sci.* **4**, 38 (2021).

6. J. P. Vernier, L. W. Thomason, J. P. Pommereau, A. Bourassa, J. Pelon, A. Garnier, A. Hauchecorne, L. Blanot, C. Trepte, D. Degenstein, Major influence of tropical volcanic eruptions on the stratospheric aerosol layer during the last decade. *Geophys. Res. Lett.* **38**, L12807 (2011).

7. S. Kremser, L. W. Thomason, M. von Hobe, M. Hermann, T. Deshler, C. Timmreck, M. Toohey, A. Stenke, J. P. Schwarz, R. Weigel, Stratospheric aerosol—Observations, processes, and impact on climate. *Rev. Geophys.* **54**, 278–335 (2016).

8. C. Brühl, J. Lelieveld, P. J. Crutzen, H. Tost, The role of carbonyl sulphide as a source of stratospheric sulphate aerosol and its impact on climate. *Atmos. Chem. Phys.* **12**, 1239–1253 (2012).

9. A. Ciucci, P. Palumbo, R. Brunetto, V. Della Corte, S. De Angelis, A. Rotundi, F. Rietmeijer, E. Zona, L. Colangeli, F. Esposito, DUSTER (dust in the upper stratosphere tracking experiment and retrieval). PRELIMINARY ANALYSIS. *Mem. Soc. Astron. Ital. Suppl.* **16**, 119 (2011).

10. A. R. Klekociuk, P. G. Brown, D. W. Pack, D. O. ReVelle, W. Edwards, R. E. Spalding, E. Tagliaferri, B. B. Yoo, J. Zagari, Meteoritic dust from the atmospheric disintegration of a large meteoroid. *Nature* **436**, 1132–1135 (2005).

11. S. Khaykin, B. Legras, S. Bucci, P. Sellitto, L. Isaksen, F. Tencé, S. Bekki, A. Bourassa, L. Rieger, D. Zawada, J. Jumelet, S. Godin-Beekmann, The 2019/20 Australian wildfires generated a persistent smoke-charged vortex rising up to 35 km altitude. *Commun. Earth Environ.* **1**, 22 (2020).

12. G. P. Kablick III, D. R. Allen, M. D. Fromm, G. E. Nedoluha, Australian PyroCb smoke generates synoptic-scale stratospheric anticyclones. *Geophys. Res. Lett.* **47**, e2020GL088101 (2020).

13. A. De Laat, D. C. Stein Zweers, R. Boers, O. N. Tuinder, A solar escalator: Observational evidence of the self-lifting of smoke and aerosols by absorption of solar radiation in the February 2009 Australian Black Saturday plume. *J. Geophys. Res. Atmos.* **117**, D04204 (2012).

14. P. Yu, O. B. Toon, C. G. Bardeen, Y. Zhu, K. H. Rosenlof, R. W. Portmann, T. D. Thornberry, R.-S. Gao, S. M. Davis, E. T. Wolf, Black carbon lofts wildfire smoke high into the stratosphere to form a persistent plume. *Science* **365**, 587–590 (2019).

15. M. Fromm, D. T. Lindsey, R. Servranckx, G. Yue, T. Trickl, R. Sica, P. Doucet, S. Godin-Beekmann, The untold story of pyrocumulonimbus. *Bull. Amer. Meteorol. Soc.* **91**, 1193–1210 (2010).

16. J. Trentmann, G. Luderer, T. Winterrath, M. Fromm, R. Servranckx, C. Textor, M. Herzog, H.-F. Graf, M. Andreae, Modeling of biomass smoke injection into the lower stratosphere by a large forest fire (Part I): Reference simulation. *Atmos. Chem. Phys.* **6**, 5247–5260 (2006).

17. H. Baars, A. Ansmann, K. Ohneiser, M. Haarig, R. Engelmann, D. Althausen, I. Hanssen, M. Gausa, A. Pietruczuk, A. Szkop, I. S. Stachlewska, D. Wang, J. Reichardt, A. Skupin, I. Mattis, T. Trickl, H. Vogelmann, F. Navas-Guzmán, A. Haefele, K. Acheson, A. A. Ruth, Boyan Tatarov, D. Müller, Q. Hu, T. Podvin, P. Goloub, I. Veselovskii, C. Pietras, M. Haeffelin, P. Fréville, M. Sicard, A. Comerón, A. J. F. García, F. M. Menéndez, C. Córdoba-Jabonero, J. L. Guerrero-Rascado, L. Alados-Arboledas, D. Bortoli, M. J. Costa, D. Dionisi, G. L. Liberti, X. Wang, A. Sannino, N. Papagiannopoulos, A. Boselli, L. Mona, G. D'Amico, S. Romano, M. R. Perrone, L. Belegante, D. Nicolae, I. Grigorov, A. Gialitaki, V. Amiridis, O. Soupiona, A. Papayannis, R.-E. Mamouri, A. Nisantzi, B. Heese, J. Hofer, Y. Y. Schechner, U. Wandinger, G. Pappalardo, The unprecedented 2017–2018 stratospheric smoke event: Decay phase and aerosol properties observed with the EARLINET. *Atmos. Chem. Phys.* **19**, 15183–15198 (2019).

18. P. Yu, S. M. Davis, O. B. Toon, R. W. Portmann, C. G. Bardeen, J. E. Barnes, H. Telg, C. Maloney, K. H. Rosenlof, Persistent stratospheric warming due to 2019–2020 Australian wildfire smoke. *Geophys. Res. Lett.* **48**, e2021GL092609 (2021).

19. S. Solomon, K. Dube, K. Stone, P. Yu, D. Kinnison, O. B. Toon, S. E. Strahan, K. H. Rosenlof, R. Portmann, S. Davis, W. Randel, P. Bernath, C. Boone, C. G. Bardeen, A. Bourassa, D. Zawada, D. Degenstein, On the stratospheric chemistry of midlatitude wildfire smoke. *Proc. Natl. Acad. Sci. U.S.A.* **119**, e2117325119 (2022).

20. G. Doglioni, V. Aquila, S. Das, P. R. Colarco, D. Zardi, Dynamical perturbation of the stratosphere by a pyrocumulonimbus injection of carbonaceous aerosols. *EGUsphere* **22**, 11049–11064 (2022).

21. S. Das, P. R. Colarco, L. D. Oman, G. Taha, O. Torres, The long-term transport and radiative impacts of the 2017 British Columbia pyrocumulonimbus smoke aerosols in the stratosphere. *Atmos. Chem. Phys.* **21**, 12069–12090 (2021).

22. H. Lestrelin, B. Legras, A. Podglajen, M. Salihoglu, Smoke-charged vortices in the stratosphere generated by wildfires and their behaviour in both hemispheres: Comparing Australia 2020 to Canada 2017. *Atmos. Chem. Phys.* **21**, 7113–7134 (2021).

23. R. Gelaro, W. McCarty, M. J. Suárez, R. Todling, A. Molod, L. Takacs, C. A. Randles, A. Darmenov, M. G. Bosilovich, R. Reichle, K. Wargan, L. Coy, R. Cullather, C. Draper, S. Akella, V. Buchard, A. Conaty, A. M. da Silva, W. Gu, G.-K. Kim, R. Koster, R. Lucchesi, D. Merkova, J. E. Nielsen, G. Partyka, S. Pawson, W. Putman, M. Rienecker, S. D. Schubert, M. Sienkiewicz, B. Zhao, The modern-era retrospective analysis for research and applications, version 2 (MERRA-2). *J. Climate* **30**, 5419–5454 (2017).

24. D. R. Allen, M. D. Fromm, G. P. Kablick III, G. E. Nedoluha, Smoke with induced rotation and lofting (SWIRL) in the stratosphere. *J. Atmos. Sci.* **77**, 4297–4316 (2020).

25. J. R. Holton, G. J. Hakim, *An Introduction to Dynamic Meteorology*. (Academic Press, 2013).

26. K. Ohneiser, A. Ansmann, H. Baars, P. Seifert, B. Barja, C. Jimenez, M. Radenz, A. Teisseire, A. Floutsi, M. Haarig, Smoke of extreme Australian bushfires observed in the stratosphere over Punta Arenas, Chile, in January 2020: Optical thickness, lidar ratios, and depolarization ratios at 355 and 532 nm. *Atmos. Chem. Phys.* **20**, 8003–8015 (2020).

27. S. Solomon, K. Stone, P. Yu, D. M. Murphy, D. Kinnison, A. R. Ravishankara, P. Wang, Chlorine activation and enhanced ozone depletion induced by wildfire aerosol. *Nature* **615**, 259–264 (2023).

28. X. Tie, G. P. Brasseur, B. Briegleb, C. Granier, Two-dimensional simulation of Pinatubo aerosol and its effect on stratospheric ozone. *J. Geophys. Res. Atmos.* **99**, 20545–20562 (1994).

29. S. Muthers, F. Arfeuille, C. C. Raible, E. Rozanov, The impacts of volcanic aerosol on stratospheric ozone and the Northern Hemisphere polar vortex: Separating radiative-

dynamical changes from direct effects due to enhanced aerosol heterogeneous chemistry. *Atmos. Chem. Phys.* **15**, 11461–11476 (2015).

30. S. Solomon, R. Portmann, R. Garcia, L. Thomason, L. Poole, M. McCormick, The role of aerosol variations in anthropogenic ozone depletion at northern midlatitudes. *J. Geophys. Res. Atmos.* **101**, 6713–6727 (1996).

31. S. Tilmes, J. H. Richter, M. J. Mills, B. Kravitz, D. G. MacMartin, R. R. Garcia, D. E. Kinnison, J. F. Lamarque, J. Tribbia, F. Vitt, Effects of different stratospheric SO_2 injection altitudes on stratospheric chemistry and dynamics. *J. Geophys. Res. Atmos.* **123**, 4654–4673 (2018).

32. P. Wang, S. Solomon, K. Stone, Stratospheric chlorine processing after the 2020 Australian wildfires derived from satellite data. *Proc. Natl. Acad. Sci. U.S.A.* **120**, e2213910120 (2023).

33. M. Santee, A. Lambert, G. Manney, N. Livesey, L. Froidevaux, J. Neu, M. Schwartz, L. Millán, F. Werner, W. Read, Prolonged and pervasive perturbations in the composition of the southern hemisphere midlatitude lower stratosphere from the australian new year's fires. *Geophys. Res. Lett.* **49**, e2021GL096270 (2022).

34. L. Teckentrup, S. P. Harrison, S. Hantson, A. Heil, J. R. Melton, M. Forrest, F. Li, C. Yue, A. Arneth, T. Hickler, S. Sitch, G. Lasslop, Response of simulated burned area to historical changes in environmental and anthropogenic factors: A comparison of seven fire models. *Biogeosciences* **16**, 3883–3910 (2019).

35. C. G. Gertler, P. A. O'Gorman, Changing available energy for extratropical cyclones and associated convection in Northern Hemisphere summer. *Proc. Natl. Acad. Sci. U.S.A.* **116**, 4105–4110 (2019).

36. P. Lin, Q. Fu, Changes in various branches of the Brewer–Dobson circulation from an ensemble of chemistry climate models. *J. Geophys. Res. Atmos.* **118**, 73–84 (2013).

37. A. Robock, L. Oman, G. Stenchikov, O. Toon, C. Bardeen, R. Turco, Climatic consequences of regional nuclear conflicts. *Atmos. Chem. Phys* **7**, 2003–2012 (2007).

38. D. S. Robertson, W. M. Lewis, P. M. Sheehan, O. B. Toon, K-Pg extinction: Reevaluation of the heat-fire hypothesis. *Eur. J. Vasc. Endovasc. Surg.* **118**, 329–336 (2013).

39. M. J. Mills, O. B. Toon, R. P. Turco, D. E. Kinnison, R. R. Garcia, Massive global ozone loss predicted following regional nuclear conflict. *Proc. Natl. Acad. Sci. U.S.A.* **105**, 5307–5312 (2008).

40. A. P. Mizzi, A. F. Arellano, D. P. Edwards, J. L. Anderson, G. G. Pfister, Assimilating compact phase space retrievals of atmospheric composition with WRF-Chem/DART: A regional chemical transport/ensemble Kalman filter data assimilation system. *Geosci. Model Dev.* **9**, 965–978 (2016).

41. A. P. Mizzi, D. P. Edwards, J. L. Anderson, Assimilating compact phase space retrievals (CPSRs): Comparison with independent observations (MOZAIC in situ and IASI retrievals) and extension to assimilation of truncated retrieval profiles. *Geosci. Model Dev.* **11**, 3727–3745 (2018).

42. G. A. Grell, S. E. Peckham, R. Schmitz, S. A. McKeen, G. Frost, W. C. Skamarock, B. Eder, Fully coupled "online" chemistry within the WRF model. *Atmos. Environ.* **39**, 6957–6975 (2005).

43. J. Anderson, T. Hoar, K. Raeder, H. Liu, N. Collins, R. Torn, A. Avellano, The data assimilation research testbed: A community facility. *Bull. Amer. Meteorol. Soc.* **90**, 1283–1296 (2009).

44. J. I. Rubin, J. S. Reid, J. A. Hansen, J. L. Anderson, B. N. Holben, P. Xian, D. L. Westphal, J. L. Zhang, Assimilation of AERONET and MODIS AOT observations using variational and ensemble data assimilation methods and its impact on aerosol forecasting skill. *J. Geophys. Res. Atmos.* **122**, 4967–4992 (2017).

45. Y. Kwon, Z. L. Yang, L. Zhao, T. J. Hoar, A. M. Toure, M. Rodell, Estimating Snow Water storage in North America using CLM4, DART, and snow radiance data assimilation. *J. Hydrometeorol.* **17**, 2853–2874 (2016).

46. C. Ma, T. Wang, A. P. Mizzi, J. L. Anderson, B. Zhuang, M. Xie, R. Wu, multiconstituent data assimilation with WRF-Chem/DART: Potential for adjusting anthropogenic emissions and improving air quality forecasts over Eastern China. *J. Geophys. Res. Atmos.* **124**, 7393–7412 (2019).

47. C. Ma, T. Wang, Z. Jiang, H. Wu, M. Zhao, B. Zhuang, S. Li, M. Xie, M. Li, J. Liu, R. Wu, Importance of bias correction in data assimilation of multiple observations over Eastern China using WRF-Chem/DART. *J. Geophys. Res.* **125**, e2019JD031465 (2020).

48. P. Ricchiazzi, S. Yang, C. Gautier, D. Sowle, SBDART: A research and teaching software tool for plane-parallel radiative transfer in the Earth's atmosphere. *Bull. Amer. Meteorol. Soc.* **79**, 2101–2114 (1998).

49. D. A. Peterson, J. R. Campbell, E. J. Hyer, M. D. Fromm, G. P. Kablick, J. H. Cossuth, M. T. DeLand, Wildfire-driven thunderstorms cause a volcano-like stratospheric injection of smoke. *NPJ Clim. Atmos. Sci.* **1**, 1–8 (2018).

50. V. Buchard, A. Da Silva, P. Colarco, A. Darmenov, C. Randles, R. Govindaraju, O. Torres, J. Campbell, R. Spurr, Using the OMI aerosol index and absorption aerosol optical depth to evaluate the NASA MERRA aerosol reanalysis. *Atmos. Chem. Phys.* **15**, 5743–5760 (2015).

51. M. S. Hammer, R. V. Martin, A. van Donkelaar, V. Buchard, O. Torres, D. A. Ridley, R. J. Spurr, Interpreting the ultraviolet aerosol index observed with the OMI satellite instrument to understand absorption by organic aerosols: Implications for atmospheric oxidation and direct radiative effects. *Atmos. Chem. Phys.* **16**, 2507–2523 (2016).

52. J. Taylor, J. Edwards, M. Glew, P. Hignett, A. Slingo, Studies with a flexible new radiation code. II: Comparisons with aircraft short-wave observations. *Q. J. Roy. Meteorol. Soc.* **122**, 839–861 (1996).

53. M. Cisewski, J. Zawodny, J. Gasbarre, R. Eckman, N. Topiwala, O. Rodriguez-Alvarez, D. Cheek, S. Hall, *Sensors, Systems, and Next-Generation Satellites XVIII* (International Society for Optics and Photonics, 2014), vol. 9241, p. 924107.

54. Z. Liu, M. Vaughan, D. Winker, C. Kittaka, B. Getzewich, R. Kuehn, A. Omar, K. Powell, C. Trepte, C. Hostetler, The CALIPSO lidar cloud and aerosol discrimination: Version 2 algorithm and initial assessment of performance. *J. Atmos. Oceanic Tech.* **26**, 1198–1213 (2009).

55. R. C. Levy, S. Mattoo, L. A. Munchak, L. A. Remer, A. M. Sayer, F. Patadia, N. C. Hsu, The Collection 6 MODIS aerosol products over land and ocean. *Atmos. Meas. Tech.* **6**, 2989–3034 (2013).

56. A. M. Sayer, L. A. Munchak, N. C. Hsu, R. C. Levy, C. Bettenhausen, M. J. Jeong, MODIS Collection 6 aerosol products: Comparison between Aqua's e-Deep blue, Dark Target, and "merged" data sets, and usage recommendations. *J. Geophys. Res. Atmos.* **119**, 13965–13989 (2014).

57. E. J. Hyer, J. S. Reid, J. Zhang, An over-land aerosol optical depth data set for data assimilation by filtering, correction, and aggregation of MODIS Collection 5 optical depth retrievals. *Atmos. Meas. Tech.* **4**, 379–408 (2011).

58. D. Stein Zweers, TROPOMI ATBD of the UVaerosol index (KNMI, 2018); https://sentinel.esa.int/documents/247904/2476257/Sentinel-5P-TROPOMI-ATBD-UV-Aerosol-Index.pdf.

59. O. Torres, P. Bhartia, J. Herman, Z. Ahmad, J. Gleason, Derivation of aerosol properties from satellite measurements of backscattered ultraviolet radiation: Theoretical basis. *J. Geophys. Res. Atmos.* **103**, 17099–17110 (1998).

60. P. Bernath, J. Crouse, R. Hughes, C. Boone, The atmospheric chemistry experiment Fourier transform spectrometer (ACE-FTS) version 4.1 retrievals: Trends and seasonal distributions. *J. Quant. Spectrosc. Radiat. Transf.* **259**, 107409 (2021).

61. G. Danabasoglu, J.-F. Lamarque, J. Bacmeister, D. A. Bailey, A. K. DuVivier, J. Edwards, L. K. Emmons, J. Fasullo, R. Garcia, A. Gettelman, C. Hannay, M. M. Holland, W. G. Large, P. H. Lauritzen, D. M. Lawrence, J. T. M. Lenaerts, K. Lindsay, W. H. Lipscomb, M. J. Mills, R.

Neale, K. W. Oleson, B. Otto-Bliesner, A. S. Phillips, W. Sacks, S. Tilmes, L. van Kampenhout, M. Vertenstein, A. Bertini, J. Dennis, C. Deser, C. Fischer, B. Fox-Kemper, J. E. Kay, D. Kinnison, P. J. Kushner, V. E. Larson, M. C. Long, S. Mickelson, J. K. Moore, E. Nienhouse, L. Polvani, P. J. Rasch, W. G. Strand, The community earth system model version 2 (CESM2). *J. Adv. Model. Earth Syst.* **12**, e2019MS001916 (2020).

62. S. J. Lin, R. B. Rood, An explicit flux-form semi-Lagrangian shallow-water model on the sphere. *Q. J. Roy. Meteorol. Soc.* **123**, 2477–2498 (1997).

63. L. K. Emmons, R. H. Schwantes, J. J. Orlando, G. Tyndall, D. Kinnison, J. F. Lamarque, D. Marsh, M. J. Mills, S. Tilmes, C. Bardeen, R. R. Buchholz, A. Conley, A. Gettelman, R. Garcia, I. Simpson, D. R. Blake, S. Meinardi, G. Petron, The chemistry mechanism in the community earth system model version 2 (CESM2). *J. Adv. Model. Earth Syst.* **12**, e2019MS001882 (2020).

64. X. Liu, P.-L. Ma, H. Wang, S. Tilmes, B. Singh, R. Easter, S. Ghan, P. Rasch, Description and evaluation of a new four-mode version of the Modal Aerosol Module (MAM4) within version 5.3 of the Community Atmosphere Model. *Geosci. Model Dev.* **9**, 505–522 (2016).

65. M. J. Iacono, J. S. Delamere, E. J. Mlawer, M. W. Shephard, S. A. Clough, W. D. Collins, Radiative forcing by long-lived greenhouse gases: Calculations with the AER radiative transfer models. *J. Geophys. Res. Atmos.* **113**, D13103 (2008).

66. S. M. Burrows, R. Easter, X. Liu, P.-L. Ma, H. Wang, S. M. Elliott, B. Singh, K. Zhang, P. J. Rasch, OCEANFILMS sea-spray organic aerosol emissions–Part 1: Implementation and impacts on clouds. *Atmos. Chem. Phys. Discuss.* 1–27 (2018).

67. H. Hersbach, B. Bell, P. Berrisford, S. Hirahara, A. Horányi, J. Muñoz-Sabater, J. Nicolas, C. Peubey, R. Radu, D. Schepers, The ERA5 global reanalysis. *Q. J. Roy. Meteorol. Soc.* **146**, 1999–2049 (2020).

68. G. R. Van Der Werf, J. T. Randerson, L. Giglio, T. T. Van Leeuwen, Y. Chen, B. M. Rogers, M. Mu, M. J. Van Marle, D. C. Morton, G. J. Collatz, Global fire emissions estimates during 1997-2016. *Earth Syst. Sci. Data* **9**, 697–720 (2017).

69. S. R. Freitas, K. M. Longo, R. Chatfield, D. Latham, M. Silva Dias, M. Andreae, E. Prins, J. Santos, R. Gielow, J. Carvalho Jr, Including the sub-grid scale plume rise of vegetation fires in low resolution atmospheric transport models. *Atmos. Chem. Phys.* **7**, 3385–3398 (2007).

70. S. Freitas, K. Longo, J. Trentmann, D. Latham, Technical note: Sensitivity of 1-D smoke plume rise models to the inclusion of environmental wind drag. *Atmos. Chem. Phys.* **10**, 585–594 (2010).

71. X. H. Pan, C. Ichoku, M. Chin, H. S. Bian, A. Darmenov, P. Colarco, L. Ellison, T. Kucsera, A. da Silva, J. Wang, T. Oda, G. Cui, Six global biomass burning emission datasets: Intercomparison and application in one global aerosol model. *Atmos. Chem. Phys.* **20**, 969–994 (2020).

72. I. R. van der Velde, G. R. van der Werf, S. Houweling, J. D. Maasakkers, T. Borsdorff, J. Landgraf, P. Tol, T. A. van Kempen, R. van Hees, R. Hoogeveen, Vast CO_2 release from Australian fires in 2019–2020 constrained by satellite. *Nature* **597**, 366–369 (2021).

73. T. C. Bond, S. J. Doherty, D. W. Fahey, P. M. Forster, T. Berntsen, B. J. DeAngelo, M. G. Flanner, S. Ghan, B. Kärcher, D. Koch, Bounding the role of black carbon in the climate system: A scientific assessment. *J. Geophys. Res. Atmos.* **118**, 5380–5552 (2013).

74. S. Akagi, R. J. Yokelson, C. Wiedinmyer, M. Alvarado, J. Reid, T. Karl, J. Crounse, P. Wennberg, Emission factors for open and domestic biomass burning for use in atmospheric models. *Atmos. Chem. Phys.* **11**, 4039–4072 (2011).

75. R. Paugam, M. Wooster, S. Freitas, M. Val Martin, A review of approaches to estimate wildfire plume injection height within large-scale atmospheric chemical transport models. *Atmos. Chem. Phys.* **16**, 907–925 (2016).

76. S. Redfern, J. K. Lundquist, O. B. Toon, D. Muñoz-Esparza, C. G. Bardeen, B. Kosović, Upper troposphere smoke injection from large areal fires. *J. Geophys. Res. Atmos.* **126**, e2020JD034332 (2021).

77. G. Luderer, J. Trentmann, K. Hungershöfer, M. Herzog, M. Fromm, M. Andreae, Small-scale mixing processes enhancing troposphere-to-stratosphere transport by pyro-cumulonimbus storms. *Atmos. Chem. Phys.* **7**, 5945–5957 (2007).

78. N. A. Kramarova, P. K. Bhartia, G. Jaross, L. Moy, P. Xu, Z. Chen, M. DeLand, L. Froidevaux, N. Livesey, D. Degenstein, Validation of ozone profile retrievals derived from the OMPS LP version 2.5 algorithm against correlative satellite measurements. *Atmos. Meas. Tech.* **11**, 2837–2861 (2018).

79. W. Tang, J. Llort, J. Weis, M. M. G. Perron, S. Basart, Z. Li, S. Sathyendranath, T. Jackson, E. Sanz Rodriguez, B. C. Proemse, A. R. Bowie, C. Schallenberg, P. G. Strutton, R. Matear, N. Cassar, Widespread phytoplankton blooms triggered by 2019–2020 Australian wildfires. *Nature* **597**, 370–375 (2021).

80. J. Wang, Z. Liu, N. Zeng, F. Jiang, H. Wang, W. Ju, Spaceborne detection of XCO_2 enhancement induced by Australian mega-bushfires. *Environ. Res. Lett.* **15**, 124069 (2020).

81. D. Liu, C. Zhou, J. K. Keesing, O. Serrano, A. Werner, Y. Fang, Y. Chen, P. Masque, J. Kinloch, A. Sadekov, Y. Du, Wildfires enhance phytoplankton production in tropical oceans. *Nat. Commun.* **13**, 1348 (2022).

82. M. Li, F. Shen, X. Sun, 2019–2020 Australian bushfire air particulate pollution and impact on the South Pacific Ocean. *Sci. Rep.* **11**, 12288 (2021).

83. T. Shiraishi, R. Hirata, Estimation of carbon dioxide emissions from the megafires of Australia in 2019–2020. *Sci. Rep.* **11**, 8267 (2021).

84. R. J. Pope, B. J. Kerridge, R. Siddans, B. G. Latter, M. P. Chipperfield, S. R. Arnold, L. J. Ventress, M. A. Pimlott, A. M. Graham, D. S. Knappett, Large enhancements in southern

hemisphere satellite-observed trace gases due to the 2019/2020 Australian wildfires. *J. Geophys. Res. Atmos.* **126**, e2021JD034892 (2021).

85. S. E. Strahan, D. Smale, S. Solomon, G. Taha, M. R. Damon, S. D. Steenrod, N. Jones, B. Liley, R. Querel, J. Robinson, Unexpected repartitioning of stratospheric inorganic chlorine after the 2020 Australian wildfires. *Geophys. Res. Lett.* **49**, e2022GL098290 (2022).

86. H. Forrister, J. Liu, E. Scheuer, J. Dibb, L. Ziemba, K. L. Thornhill, B. Anderson, G. Diskin, A. E. Perring, J. P. Schwarz, P. Campuzano-Jost, D. A. Day, B. B. Palm, J. L. Jimenez, A. Nenes, R. J. Weber, Evolution of brown carbon in wildfire plumes. *Geophys. Res. Lett.* **42**, 4623–4630 (2015).

87. J. Mok, N. A. Krotkov, A. Arola, O. Torres, H. Jethva, M. Andrade, G. Labow, T. F. Eck, Z. Li, R. R. Dickerson, Impacts of brown carbon from biomass burning on surface UV and ozone photochemistry in the Amazon Basin. *Sci. Rep.* **6**, 36940 (2016).

88. K. Jansen, E. Browne, M. Tolbert, in *AGU Fall Meeting Abstracts* (American Geophysical Union, 2021), vol. 2021, p. A55O-1609.

89. R. M. Hoesly, S. J. Smith, L. Feng, Z. Klimont, G. Janssens-Maenhout, T. Pitkanen, J. J. Seibert, L. Vu, R. J. Andres, R. M. Bolt, T. C. Bond, L. Dawidowski, N. Kholod, J. I. Kurokawa, M. Li, L. Liu, Z. Lu, M. C. P. Moura, P. R. O'Rourke, Q. Zhang, Historical (1750–2014) anthropogenic emissions of reactive gases and aerosols from the Community Emissions Data System (CEDS). *Geosci. Model Dev.* **11**, 369–408 (2018).

90. J. W. Hurrell, J. J. Hack, D. Shea, J. M. Caron, J. Rosinski, A new sea surface temperature and sea ice boundary dataset for the Community Atmosphere Model. *J. Climate* **21**, 5145–5153 (2008).

91. M. J. Schwartz, M. L. Santee, H. C. Pumphrey, G. L. Manney, A. Lambert, N. J. Livesey, L. Millán, J. L. Neu, W. G. Read, F. Werner, Australian new year’s pyrocb impact on stratospheric composition. *Geophys. Res. Lett.* **47**, e2020GL090831 (2020).

92. A. Molod, L. Takacs, M. Suarez, J. Bacmeister, Development of the GEOS-5 atmospheric general circulation model: Evolution from MERRA to MERRA2. *Geosci. Model Dev.* **8**, 1339–1356 (2015).

93. K. Ohneiser, A. Ansmann, J. Witthuhn, H. Deneke, A. Chudnovsky, G. Walter, Self-lofting of wildfire smoke in the troposphere and stratosphere caused by radiative heating: Simulations vs space lidar observations. *Atmos. Chem. Phys. Discuss.* **2022**, 1–41 (2022).

94. G. D'Angelo, S. Guimond, J. Reisner, D. A. Peterson, M. Dubey, Contrasting stratospheric smoke mass and lifetime from 2017 Canadian and 2019/2020 Australian megafires: Global simulations and satellite observations. *J. Geophys. Res. Atmos.* **127**, e2021JD036249 (2022).

95. R. González, C. Toledano, R. Román, D. Mateos, E. Asmi, E. Rodríguez, I. C. Lau, J. Ferrara, R. D'Elia, J. C. Antuña-Sánchez, Characterization of stratospheric smoke particles over the Antarctica by remote sensing instruments. *Remote Sens. (Basel)* **12**, 3769 (2020).

96. E. Hirsch, I. Koren, Record-breaking aerosol levels explained by smoke injection into the stratosphere. *Science* **371**, 1269–1274 (2021).

97. M. Haarig, A. Ansmann, H. Baars, C. Jimenez, I. Veselovskii, R. Engelmann, D. Althausen, Depolarization and lidar ratios at 355, 532, and 1064 nm and microphysical properties of aged tropospheric and stratospheric Canadian wildfire smoke. *Atmos. Chem. Phys.* **18**, 11847–11861 (2018).

98. Q. Hu, P. Goloub, I. Veselovskii, J. A. Bravo-Aranda, I. E. Popovici, T. Podvin, M. Haeffelin, A. Lopatin, O. Dubovik, C. Pietras, X. Huang, B. Torres, C. Chen, Long-range-transported Canadian smoke plumes in the lower stratosphere over northern France. *Atmos. Chem. Phys.* **19**, 1173–1193 (2019).

99. K. Ohneiser, A. Ansmann, B. Kaifler, A. Chudnovsky, B. Barja, D. A. Knopf, N. Kaifler, H. Baars, P. Seifert, D. Villanueva, C. Jimenez, M. Radenz, R. Engelmann, I. Veselovskii, F. Zamorano, Australian wildfire smoke in the stratosphere: The decay phase in 2020/2021 and impact on ozone depletion. *Atmos. Chem. Phys.* **22**, 7417–7442 (2022).

100. A. Ansmann, K. Ohneiser, R.-E. Mamouri, D. A. Knopf, I. Veselovskii, H. Baars, R. Engelmann, A. Foth, C. Jimenez, P. Seifert, Tropospheric and stratospheric wildfire smoke profiling with lidar: Mass, surface area, CCN, and INP retrieval. *Atmos. Chem. Phys.* **21**, 9779–9807 (2021).

101. J. Ditas, N. Ma, Y. Zhang, D. Assmann, M. Neumaier, H. Riede, E. Karu, J. Williams, D. Scharffe, Q. Wang, Strong impact of wildfires on the abundance and aging of black carbon in the lowermost stratosphere. *Proc. Natl. Acad. Sci. U.S.A.* **115**, E11595–E11603 (2018).

102. V. Eyring, J. M. Arblaster, I. Cionni, J. Sedláček, J. Perlwitz, P. J. Young, S. Bekki, D. Bergmann, P. Cameron-Smith, W. J. Collins, Long-term ozone changes and associated climate impacts in CMIP5 simulations. *J. Geophys. Res. Atmos.* **118**, 5029–5060 (2013).

103. G. Stenchikov, A. Robock, V. Ramaswamy, M. D. Schwarzkopf, K. Hamilton, S. Ramachandran, Arctic Oscillation response to the 1991 Mount Pinatubo eruption: Effects of volcanic aerosols and ozone depletion. *J. Geophys. Res. Atmos.* **107**, ACL 28-21-ACL 28-16 (2002).

104. F. Tencé, J. Jumelet, S. Bekki, S. Khaykin, A. Sarkissian, P. Keckhut, Australian Black Summer smoke observed by lidar at the French Antarctic station Dumont d'Urville. *J. Geophys. Res. Atmos.* **127**, e2021JD035349 (2022).

105. A. Ansmann, K. Ohneiser, A. Chudnovsky, D. A. Knopf, E. W. Eloranta, D. Villanueva, P. Seifert, M. Radenz, B. Barja, F. Zamorano, C. Jimenez, R. Engelmann, H. Baars, H. Griesche, J. Hofer, D. Althausen, U. Wandinger, Ozone depletion in the Arctic and Antarctic stratosphere induced by wildfire smoke. *Atmos. Chem. Phys. Discuss.* **22**, 11701–11726 (2022).

106. G. Fiocco, D. Fu'a, G. Visconti, *The Mount Pinatubo eruption: Effects on the Atmosphere and Climate* (Springer Science & Business Media, 2013), vol. 42.

107. D. Hofmann, S. Oltmans, W. Komhyr, J. Harris, J. Lathrop, A. Langford, T. Deshler, B. Johnson, A. Torres, W. Matthews, Ozone loss in the lower stratosphere over the United States

in 1992–1993: Evidence for heterogeneous chemistry on the Pinatubo aerosol. *Geophys. Res. Lett.* **21**, 65–68 (1994).

108. D. Hofmann, S. Oltmans, J. Harris, W. Komhyr, J. Lathrop, T. DeFoor, D. Kuniyuki, Ozonesonde measurements at Hilo, Hawaii following the eruption of Pinatubo. *Geophys. Res. Lett.* **20**, 1555–1558 (1993).

109. M. A. Diallo, F. Ploeger, M. I. Hegglin, M. Ern, J. U. Grooß, S. Khaykin, M. Riese, Stratospheric water vapour and ozone response to the quasi-biennial oscillation disruptions in 2016 and 2020. *Atmos. Chem. Phys.* **22**, 14303–14321 (2022).

110. L. Magaritz-Ronen, S. Raveh-Rubin, Wildfire smoke highlights troposphere-to-stratosphere pathway. *Geophys. Res. Lett.* **48**, e2021GL095848 (2021).

111. B. G. Martinsson, J. Friberg, O. S. Sandvik, M. Hermann, P. F. J. van Velthoven, A. Zahn, Formation and composition of the UTLS aerosol. *NPJ Clim. Atmos. Sci.* **2**, 40 (2019).

112. A. Podglajen, B. Legras, G. Lapeyre, R. Plougonven, V. Zeitlin, V. Brémaud, P. Sellitto, Dynamics of diabatically forced anticyclonic plumes in the stratosphere. *Q. J. Roy. Meteorol. Soc.* **150**, 1538–1565 (2023).

113. D. W. Waugh, Subtropical stratospheric mixing linked to disturbances in the polar vortices. *Nature* **365**, 535–537 (1993).

114. A. Ansmann, K. Ohneiser, A. Chudnovsky, H. Baars, R. Engelmann, CALIPSO aerosol-typing scheme misclassified stratospheric fire smoke: Case study from the 2019 Siberian wildfire season. *Front. Environ. Sci.* **9**, 769852 (2021).

115. P. A. O'Gorman, Precipitation extremes under climate change. *Curr. Clim. Change Rep.* **1**, 49–59 (2015).

116. D. A. Peterson, E. J. Hyer, J. R. Campbell, J. E. Solbrig, M. D. Fromm, A conceptual model for development of intense pyrocumulonimbus in western North America. *Mon. Weather Rev.* **145**, 2235–2255 (2017).

117. B. Abish, P. Joseph, O. M. Johannessen, Climate change in the subtropical jetstream during 1950–2009. *Adv. Atmos. Sci.* **32**, 140–148 (2015).

118. W. M. Jolly, M. A. Cochrane, P. H. Freeborn, Z. A. Holden, T. J. Brown, G. J. Williamson, D. M. Bowman, Climate-induced variations in global wildfire danger from 1979 to 2013. *Nat. Commun.* **6**, 7537 (2015).

119. J. T. Abatzoglou, A. P. Williams, Impact of anthropogenic climate change on wildfire across western US forests. *Proc. Natl. Acad. Sci. U.S.A.* **113**, 11770–11775 (2016).

120. A. L. Westerling, H. G. Hidalgo, D. R. Cayan, T. W. Swetnam, Warming and earlier spring increase western U.S. forest wildfire activity. *Science* **313**, 940–943 (2006).

121. N. J. Abram, B. J. Henley, A. S. Gupta, T. J. Lippmann, H. Clarke, A. J. Dowdy, J. J. Sharples, R. H. Nolan, T. Zhang, M. J. Wooster, Connections of climate change and variability to large and extreme forest fires in southeast Australia. *Commun. Earth Environ.* **2**, 1–17 (2021).

122. T. Kitzberger, D. A. Falk, A. L. Westerling, T. W. Swetnam, Direct and indirect climate controls predict heterogeneous early-mid 21st century wildfire burned area across western and boreal North America. *PLOS ONE* **12**, e0188486 (2017).

123. Y. Chen, D. M. Romps, J. T. Seeley, S. Veraverbeke, W. J. Riley, Z. A. Mekonnen, J. T. Randerson, Future increases in Arctic lightning and fire risk for permafrost carbon. *Nat. Clim. Change* **11**, 404–410 (2021).

124. N. Andela, D. C. Morton, L. Giglio, Y. Chen, G. R. van der Werf, P. S. Kasibhatla, R. S. DeFries, G. J. Collatz, S. Hantson, S. Kloster, D. Bachelet, M. Forrest, G. Lasslop, F. Li, S. Mangeon, J. R. Melton, C. Yue, J. T. Randerson, A human-driven decline in global burned area. *Science* **356**, 1356–1362 (2017).

125. J. E. Penner, R. E. Dickinson, C. A. O'Neill, Effects of aerosol from biomass burning on the global radiation budget. *Science* **256**, 1432–1434 (1992).

126. O. B. Toon, C. G. Bardeen, A. Robock, L. Xia, H. Kristensen, M. McKinzie, R. J. Peterson, C. S. Harrison, N. S. Lovenduski, R. P. Turco, Rapidly expanding nuclear arsenals in Pakistan and India portend regional and global catastrophe. *Sci. Adv.* **5**, eaay5478 (2019).

127. A. Robock, B. Zambri, Did smoke from city fires in world war II cause global cooling? *J. Geophys. Res. Atmos.* **123**, 10314–310325 (2018).

128. B. Heinold, H. Baars, B. Barja, M. Christensen, A. Kubin, K. Ohneiser, K. Schepanski, N. Schutgens, F. Senf, R. Schrödner, D. Villanueva, I. Tegen, Important role of stratospheric injection height for the distribution and radiative forcing of smoke aerosol from the 2019–2020 Australian wildfires. *Atmos. Chem. Phys.* **22**, 9969–9985 (2022).

129. P. Sellitto, R. Belhadji, C. Kloss, B. Legras, Radiative impacts of the Australian bushfires 2019–2020 – Part 1: Large-scale radiative forcing. *Atmos. Chem. Phys.* **22**, 9299–9311 (2022).

130. D. Y. Chang, J. Yoon, J. Lelieveld, S. K. Park, S. S. Yum, J. Kim, S. Jeong, Direct radiative forcing of biomass burning aerosols from the extensive Australian wildfires in 2019–2020. *Environ. Res. Lett.* **16**, 044041 (2021).

131. C.-A. Papanikolaou, P. Kokkalis, O. Soupiona, S. Solomos, A. Papayannis, M. Mylonaki, D. Anagnou, R. Foskinis, M. Gidarakou, Australian bushfires (2019–2020): Aerosol optical properties and radiative forcing. *Atmos.* **13**, 867 (2022).

132. C. Trepte, M. Vaughan, S. Kato, S. Young, in *AGU Fall Meeting Abstracts* (American Geophysical Union, 2009), vol. 2009, p. A43E-04.
